# Integrated analysis reveals microRNA networks coordinately expressed with key proteins in breast cancer

**DOI:** 10.1186/s13073-015-0135-5

**Published:** 2015-02-02

**Authors:** Miriam Ragle Aure, Sandra Jernström, Marit Krohn, Hans Kristian Moen Vollan, Eldri U Due, Einar Rødland, Rolf Kåresen, Oslo Breast Cancer Research Consortium (OSBREAC), Prahlad Ram, Yiling Lu, Gordon B Mills, Kristine Kleivi Sahlberg, Anne-Lise Børresen-Dale, Ole Christian Lingjærde, Vessela N Kristensen

**Affiliations:** Department of Genetics, Institute for Cancer Research, Oslo University Hospital, The Norwegian Radium Hospital, Oslo, 0310 Norway; K.G. Jebsen Centre for Breast Cancer Research, Institute for Clinical Medicine, University of Oslo, Oslo, 0316 Norway; Department of Oncology, Division of Surgery, Cancer and Transplantation, Oslo University Hospital, The Norwegian Radium Hospital, Oslo, 0310 Norway; Department of Tumor Biology, Institute for Cancer Research, Oslo University Hospital, The Norwegian Radium Hospital, Oslo, 0310 Norway; Centre for Cancer Biomedicine, University of Oslo, Oslo, 0316 Norway; Department of Computer Science, University of Oslo, Oslo, 0316 Norway; Institute of Clinical Medicine, University of Oslo, Oslo, 0316 Norway; Department of Systems Biology, The University of Texas M.D. Anderson Cancer Center, Houston, TX 77030 USA; Department of Research, Vestre Viken Hospital Trust, Drammen, 3004 Norway; Department of Clinical Molecular Biology and Laboratory Science (EpiGen), Division of Medicine, Akershus University Hospital, Lørenskog, 1478 Norway

## Abstract

**Background:**

The role played by microRNAs in the deregulation of protein expression in breast cancer is only partly understood. To gain insight, the combined effect of microRNA and mRNA expression on protein expression was investigated in three independent data sets.

**Methods:**

Protein expression was modeled as a multilinear function of powers of mRNA and microRNA expression. The model was first applied to mRNA and protein expression for 105 selected cancer-associated genes and to genome-wide microRNA expression from 283 breast tumors. The model considered both the effect of one microRNA at a time and all microRNAs combined. In the latter case the Lasso penalized regression method was applied to detect the simultaneous effect of multiple microRNAs.

**Results:**

An interactome map for breast cancer representing all direct and indirect associations between the expression of microRNAs and proteins was derived. A pattern of extensive coordination between microRNA and protein expression in breast cancer emerges, with multiple clusters of microRNAs being associated with multiple clusters of proteins. Results were subsequently validated in two independent breast cancer data sets. A number of the microRNA-protein associations were functionally validated in a breast cancer cell line.

**Conclusions:**

A comprehensive map is derived for the co-expression in breast cancer of microRNAs and 105 proteins with known roles in cancer, after filtering out the in-*cis* effect of mRNA expression. The analysis suggests that group action by several microRNAs to deregulate the expression of proteins is a common *modus operandi* in breast cancer.

**Electronic supplementary material:**

The online version of this article (doi:10.1186/s13073-015-0135-5) contains supplementary material, which is available to authorized users.

## Background

Since the initial discovery of microRNAs (miRNAs) as post-trancriptional regulators of gene expression, a complex network of coordinate regulatory interactions between miRNAs and mRNAs has been unravelled (see, for example, [1]). Causal links between miRNA dysregulation and tumor development have been established in breast cancer and other types of cancer. In breast cancer, the expression of some miRNAs is related to the molecular subtypes as defined by mRNA expression [2,3], while other miRNAs appear to be expressed independently of gene expression-based subtypes. The two main mechanisms by which miRNAs directly regulate protein expression are through mRNA degradation and translational inhibition [4]. Some studies suggest that miRNAs can upregulate the expression of a subset of proteins in a given RNA sequence context and under certain cellular conditions, such as quiescence, or even induce expression by binding to complementary promoter elements [5]; however, the predominant effect of direct miRNA regulation is a decrease of mRNA target levels [6]. The molecular signature of miRNA regulation depends on the mode of regulatory action, as illustrated in Additional file 1. In particular, miRNA destabilization of an mRNA affects the mRNA level, while the mRNA-protein response curve remains the same. On the other hand, miRNA repression of protein translation does not affect the mRNA level *per se*, but the slope and potentially the shape of the protein response curve will change.

Indirect effects of miRNAs on protein expression are likely to be abundant. For example, interventions by miRNAs can, in principle, occur at any stage of a signaling pathway [7] and may include the involvement of miRNAs in feedback loops and feedforward cascades with transcription factors and signaling molecules [8]. Network focused studies have emphasized the interplay between miRNAs and transcription factors and have revealed how miRNAs play pivotal regulatory roles in disease-specific subnetworks such as those found in breast cancer [9]. Direct and indirect effects of miRNAs can involve joint regulation of multiple genes by a single miRNA species and joint regulation of a single gene by multiple miRNAs. The importance of mapping such relationships is suggested by studies showing that miRNAs can have a relatively weak regulatory effect on individual proteins [10,11], and at the same time a strong effect on the pathway activation level by coordinately targeting multiple genes in the same pathway [12].

The main goal of this study was to examine the global pattern of association between whole-genome miRNA expression and the expression of selected proteins in breast cancer. An association in this context translates to a mode of variation in miRNA expression that is reflected in a corresponding mode of variation in protein expression. While causality cannot be inferred from such associations, the totality of associations reflects the degree of coordination between the miRNAs and the proteins and represents an upper bound on the number of strong causal direct and indirect links between miRNAs and proteins. Adjusting for mRNA expression when assessing the relationship between protein expression and miRNA expression (thus considering three different molecular levels at the same time) is an important feature of the proposed approach. Failure to account for the commonly strong effect of mRNA expression on protein expression may lead to serious overestimation of the effect of miRNAs on protein expression in cases where the miRNA expression correlates with mRNA expression. By including mRNA expression in the model, this potential confounder effect is accounted for, and as a result the risk of reporting false positive associations between miRNAs and proteins is reduced. Whole-genome miRNA expression profiles were analyzed with respect to the mRNA/protein levels of 105 genes with known relevance in cancer. To detect effects of individual miRNAs on protein expression, the often strong and potentially correlated effects of in*-cis* mRNA expression on protein expression were separated out by modeling protein expression as a joint function of in*-cis* mRNA and whole-genome miRNA expression. The effect of individual miRNAs, as well as multiple miRNAs simultaneously, was assessed.

## Methods

An outline of the approach for assessing the effect of miRNAs on protein expression is shown in Additional file 2. Tumors from 283 primary breast cancer patients belonging to the Oslo2 cohort were profiled for genome-wide miRNA and mRNA expression using Agilent microarrays (Agilent Technologies, Santa Clara, CA, USA), as well as for protein expression using reverse-phase protein arrays (RPPA) [13] for a selected panel of 105 cancer-related proteins. The Oslo2 study is a consecutive study collecting material from breast cancer patients with primary operable disease (cT1 to cT2) in several hospitals in south-eastern Norway. Inclusion of patients started in 2006 and is still ongoing. The study was approved by the Norwegian Regional Committee for Medical Research Ethics (approval number 1.2006.1607, amendment 1.2007.1125), and patients have given written consent for the use of material for research purposes. All experimental methods performed are in compliance with the Helsinki Declaration. All computational analyses were performed in R [14] unless otherwise specified. See Additional file 3 for details about the miRNA, mRNA and protein expression profiling and *in silico* miRNA target predictions. The miRNA and mRNA expression data have been submitted to the Gene Expression Omnibus (GEO) database as a SuperSeries record with accession number GSE58215. The protein expression data can be found in Additional file 4.

Two independent breast cancer data sets with miRNA, mRNA and protein expression data available were used for model validation. The Danish Breast Cancer Cooperative Group (DBCG) cohort [15] had available data from these three levels for 128 primary breast tumors. The mRNA expression (Applied Biosystems, Foster City, CA, USA) and protein expression (RPPA) data were published in [16], and the miRNA expression data (Agilent Technologies) were published in [17]. For The Cancer Genome Atlas (TCGA) data, miRNA and mRNA sequencing data (Illumina, San Diego, CA, USA) and RPPA expression data published in [18] were downloaded from Broad GDAC Firehose (accessed 15 January 2014). Molecular data for all three levels were available for 395 primary breast tumors. Altogether, 348 miRNAs and 34 proteins overlapped the Oslo2, DBCG and TCGA data sets, and these overlapping miRNAs and proteins were considered when comparing the data sets.

### Modeling the relationship between miRNA, mRNA and protein expression

In order to make results comparable across mRNAs, miRNAs and proteins, all expression values were standardized to the same range after and in addition to ordinary normalization (Additional file 3). If *X*_1_, *X*_2_, … , *X*_*n*_ are the expression values (not log_2_-transformed) for a miRNA, mRNA or protein, then standardized values are defined as:

$$ {X}_i^{*}=\left({X}_i- \min \left({X}_i\right)\right)/\left( \max \left({X}_i\right)- \min \left({X}_i\right)\right) + {\delta}_0 $$

where δ_0_ > 0 is a small constant ensuring that the resulting values are positive and thus eligible to log-transformation (we used δ_0_ = 0.1).

In order to model protein expression as a function of mRNA expression (disregarding for the moment all other factors that may influence protein expression), a decision has to be made regarding the form of the functional relationship. Here, we first derive mathematically a linear model for this relationship under very simplistic conditions (to be outlined), and this model is subsequently generalized to encompass a large and more realistic range of relationships. At any time, a proportion of mRNA is translated into protein and a proportion of protein degrades. Mathematically, this can be modeled as:

$$ P^{\prime }(t) = aE(t) - bP(t) $$

where *P*(*t*) and *E*(*t*) denote protein and mRNA expression levels, respectively, at time *t*. Here, *P*’(*t*) denotes the derivative of *P*(*t*), and *a* > 0 and *b* > 0 are gene/protein-specific rates of translation and degradation, respectively. We assume that gene expression is relatively constant, that is, *E*(*t*) = *E*, over the short time interval required for the protein expression to fixate, leading to a first-order linear differential equation with constant coefficients and with solution (see, for example, [19]):

$$ P(t) = \left(a/b\right)E + C\  \exp \left(-bt\right) $$

It follows that the protein expression equals *P* = (*a*/*b*) ⋅ *E* after fixation. This important relationship implies that the protein expression will be proportional to the mRNA expression, with a constant of proportionality depending only on the ratio of the rates of translation and degradation. This ratio will be allowed in the present study to depend on the expression of miRNAs. Considering first the dependency on one miRNA, a flexible and convenient model encompassing the above relationship is:

1 $$ P=C\cdot {M}^{\beta}\cdot {E}^{\gamma } $$

where *M* denotes the expression of the miRNA and *β* and *γ* are coefficients to be estimated. For example, *β* = −1 and *γ* = 1 implies that the protein level is proportional to the mRNA level and inversely proportional to the miRNA level (consistent with, for example, an inhibitory effect on translation). Other combinations of parameter values permit the model to adapt to other functional relationships. The model for dependency on multiple independently acting miRNAs is:

2 $$ P=C\cdot {M}_1^{\beta_1}\cdot \cdot \cdot \cdot \cdot {M}_{421}^{\beta_{421}}\cdot {E}^{\gamma } $$

Here, *M*_1_, …, *M*_421_ denote the expressions of the miRNAs, and the coefficients *β*_1_, …, *β*_421_ are the effects of the miRNAs (with *β*_*k*_ = 0 implying no effect of the *k*^th^ miRNA). The above equation may be interpreted as follows: the quantity *E*^*γ*^ reflects the net amount of transcripts, and the quantity ${M}_{421}^{\beta_{421}}$ is the effect on protein expression caused by the 421st miRNA and so forth. Finally, the constant *C* reflects underlying properties of the gene that might influence the rate of translation/degradation.

### Model fitting

To fit the above models to the data, it is more convenient to consider log-transformed equations. For model 1 this becomes:

3 $$ \log P = \alpha + \beta \log {M}_k + \gamma \log E+\varepsilon $$

Here, the index *k* denotes a specific miRNA and the error term ε emphasizes the statistical nature of the relationship. For each combination of a protein and a miRNA, we fit the model above to the samples using linear regression and obtain estimates ${\widehat{\alpha}}_{ij},\ {\widehat{\beta}}_{ij},{\widehat{\gamma}}_{ij}$ and corresponding *P*-values for the i’th protein and the j’th miRNA. Considering all *N* estimated miRNA effects ${\widehat{\beta}}_{ij}$ on protein expression, we calculate for any given *P*-value threshold P* the number *S* of significant effects. An estimate of the corresponding false discovery rate (FDR) can be found by dividing the expected number of false positives by the number of actual positives. The most conservative estimate for the FDR is found by assuming that the tests are independent and that all the *N* null hypotheses are true, in which case the expected number of false positives is the number of tests times the chosen level of significance. This leads to the estimate *FDR* = (NP*)/*S*. The *P*-value threshold was chosen to ensure FDR ≤0.01.

For the multivariate model involving all miRNAs, the log-transformed equations become:

4 $$ \log P=\alpha + {\displaystyle \sum_{j=1}^{421}}{\beta}_j \log {M}_j + \gamma\ \log E+\varepsilon $$

and we fit the model for each protein to obtain estimates ${\widehat{\alpha}}_i,\ {\widehat{\beta}}_{ij},{\widehat{\gamma}}_i$. A mild restriction was enforced on the model by constraining regression coefficients found to be nominally nonsignificant (*P* > 0.01 with no correction for multiple comparisons) in the univariate model to be zero. A penalized least squares regression scheme was then used to accommodate the large number of coefficients to be estimated. Several methods exist for this purpose; here, the Lasso was chosen (as implemented in the R package GLMnet [20]) because this method also implicitly performs variable selection. The Lasso imposes a penalty on the parameters during likelihood optimization, essentially constraining the magnitude of the sum of the absolute values of the parameter values to be small [20]. The trade-off between goodness-of-fit to the data and low penalty is determined by a penalty parameter; the value of this parameter was determined using cross-validation. The variables selected by the Lasso are informally analogous to the significant coefficients in the univariate model. For brevity, the non-zero coefficients in the multivariate model were denoted significant effects.

### Detection of coordinated effects of miRNAs

Let B denote the 105 × 421 table of miRNA coefficients found in Oslo2 with either the univariate or the multivariate model described above. Assuming that rows and columns in the table are ordered appropriately, coordinated effects of multiple miRNAs on a group of proteins are detectable as a block of significant miRNA coefficients of the same sign. To visualize this, we used a heatmap to display the miRNA coefficients listed in Additional file 4C, ordering rows and columns with hierarchical clustering based on Pearson correlation and complete linkage. Clusters were then identified with the Partitioning Algorithm using Recursive Thresholding (PART) method available in the package clusterGenomics in the Comprehensive R Archive Network (CRAN). For more details, see [21].

### miRNA library screen by RPPA

The MDA-MB-231 cell line was received from the Characterized Cell Line Core Facility at the MD Anderson Cancer Center. This cell line was authenticated on 10 April 2014 by the short tandem repeat method, which was performed in the Characterized Cell Line Core Facility, and the results demonstrated perfect match to the NCI public database.

The miRIDIAN 13.1 miRNA library was designed and synthesized by Dharmacon (Lafayette, CO, USA). MDA-MB-231 cells (5,000 cells/well) were transfected with miRNA mimics (50 nM) in 96-well plates using DharmaFECT transfection reagent (Dharmacon), according to the manufacturer’s instructions. For RPPA analysis, cells were lysed 72 h after transfection and cellular proteins were denatured by 1% SDS (with beta-mercaptoethanol) and diluted in five two-fold serial dilutions in dilution buffer (lysis buffer containing 1% SDS). Serial diluted lysates were arrayed on nitrocellulose-coated slides (Grace Biolab, Bend, Oregon, USA) by Aushon 2470 Arrayer (Aushon BioSystems, Billerica, MA, USA). A total of 5,808 array spots were arranged on each slide, including the spots corresponding to positive and negative controls prepared from mixed cell lysates or dilution buffer, respectively. Each slide was probed with a validated primary antibody plus a biotin-conjugated secondary antibody. Only antibodies with a Pearson correlation coefficient between RPPA and western blotting of greater than 0.7 were used in the RPPA study. The signal was obtained and the slides scanned, analyzed and quantified as described under the ‘Protein expression profiling’ paragraph in Additional file 3. The RPPA data were normalized to the controls, log_2_-transformed and converted into standard scores by subtracting the mean of the whole screen (for a given antibody) and dividing by the standard deviation of the whole screen (for a given antibody). Values ±2 × standard deviation were considered statistically significant, which corresponded to a threshold of ±1.96.

## Results

### The mRNA-protein relationship

**Figure 1:**
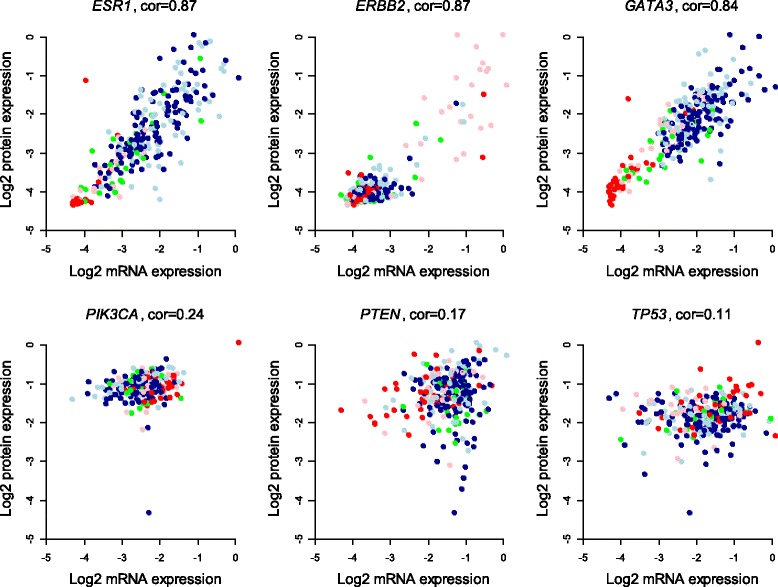
**Relationships between mRNA and protein for selected genes.** Each dot represents a patient and the colors indicate PAM50 expression subtype (red, basal-like; pink, HER2-enriched; green, normal-like; dark blue, luminal A; light blue, luminal B). Pearson correlation between mRNA and protein expression is indicated above each plot.

The correlation between mRNA expression and protein expression in the primary Oslo2 data set ranged from −0.19 (*KRAS*) to 0.87 (*ERBB2, ESR1*, *PGR*, and *AR*) (Table 1). The correlations followed a right-skewed distribution with a median value of 0.32 and a standard deviation of 0.36 (Additional file 5). Examples of low- and high-correlation relationships and dependence between protein expression and expression subtype for some breast cancer-associated proteins are shown in Figure 1, while the scatterplots of all 105 mRNA-protein relationships are given in Additional file 6. These relations (when present) were observed to be often fairly linear when both mRNA and protein expression are represented on a log-transformed scale (see Methods for a theoretical argument supporting this observation). On the other hand, the mRNA-protein relationships are generally quite noisy, suggesting that predictions of protein expression may be improved by taking into account additional factors, such as miRNAs.

**Table 1:** **The protein panel**

**Gene symbol**	**Protein**	**mRNA-protein Pearson correlation**	**Correlation** ***P*** **-value**
*ACACA*	ACC1	0.67	0.00
*AKT1*	Akt1	0.34	0.00
*AKT2*	Akt2	0.05	0.42
*AKT3*	Akt3	0.00	0.94
*ANXA1*	Annexin I	0.60	0.00
*AR*	AR	0.87	0.00
*BAK1*	Bak	0.30	0.00
*BAX*	Bax	0.29	0.00
*BCL2*	Bcl-2	0.85	0.00
*BCL2L1*	Bcl-X	0.27	0.00
*BCL2L11*	Bim	0.43	0.00
*BECN1*	Beclin	0.07	0.22
*BID*	Bid	0.12	0.05
*BIRC2*	cIAP	0.14	0.01
*BRAF*	B-Raf	0.29	0.00
*CASP8*	Caspase-8	0.14	0.02
*CAV1*	Caveolin-1	0.53	0.00
*CCNB1*	Cyclin B1	0.78	0.00
*CCND1*	Cyclin D1	0.59	0.00
*CCNE1*	Cyclin E1	0.74	0.00
*CDH1*	E-cadherin	0.65	0.00
*CDH2*	N-cadherin	−0.02	0.69
*CDH3*	P-cadherin	0.40	0.00
*CDK1*	CDK1	0.21	0.00
*CDKN1B*	p27	0.66	0.00
*CHEK1*	Chk1	0.11	0.06
*CHEK2*	Chk2	0.69	0.00
*CLDN7*	Claudin 7	0.47	0.00
*COL6A1*	Collagen VI	−0.03	0.67
*CTNNA1*	alpha-Catenin	0.27	0.00
*CTNNB1*	beta-Catenin	0.04	0.53
*DIABLO*	Smac	0.27	0.00
*DVL3*	Dvl3	0.26	0.00
*EEF2*	eEF2	−0.10	0.10
*EEF2K*	eEF2K	0.55	0.00
*EGFR*	EGFR	0.39	0.00
*EIF4E*	eIF4E	0.25	0.00
*EIF4EBP1*	4EBP1	0.68	0.00
*ERBB2*	HER2	0.87	0.00
*ERBB3*	HER3	0.46	0.00
*ERCC1*	ERCC1	−0.07	0.21
*ERRFI1*	MIG-6	0.13	0.03
*ESR1*	ER-alpha	0.87	0.00
*FN1*	Fibronectin	0.68	0.00
*FOXO3*	FOXO3a	0.32	0.00
*GAB2*	GAB2	0.78	0.00
*GATA3*	GATA3	0.84	0.00
*GSK3A*	GSK3-alpha	0.29	0.00
*GSK3B*	GSK3-beta	0.20	0.00
*IGF1R*	IGF-1R-beta	0.83	0.00
*IGFBP2*	IGFBP2	0.76	0.00
*INPP4B*	INPP4B	0.86	0.00
*IRS1*	IRS1	0.63	0.00
*KDR*	VEGFR2	−0.02	0.75
*KIT*	c-Kit	0.76	0.00
*KRAS*	K-Ras	−0.19	0.00
*MAP2K1*	MEK1	−0.08	0.20
*MAPK14*	p38 MAPK	0.12	0.04
*MAPK9*	JNK2	0.44	0.00
*MAPT*	Tau	0.13	0.04
*MET*	c-Met	0.18	0.00
*MRE11A*	Mre11	0.08	0.16
*MSH2*	MSH2	0.53	0.00
*MSH6*	MSH6	0.53	0.00
*MYC*	c-Myc	0.18	0.00
*NCOA3*	AIB1	0.34	0.00
*NF2*	NF2	0.31	0.00
*NOTCH1*	Notch1	0.39	0.00
*NOTCH3*	Notch3	0.40	0.00
*PARK7*	DJ-1	0.53	0.00
*PCNA*	PCNA	0.47	0.00
*PECAM1*	CD31	0.21	0.00
*PGR*	PR	0.87	0.00
*PIK3CA*	PI3K-p110-alpha	0.24	0.00
*PIK3R1*	PI3 kinase, p85	0.27	0.00
*PRKCA*	PKC-alpha	0.56	0.00
*PRKAA1*	AMPK alpha	0.37	0.00
*PTCH1*	PTCH	0.21	0.00
*PTEN*	PTEN	0.17	0.00
*PTGS2*	COX-2	0.28	0.00
*PTK2*	FAK	0.05	0.39
*PXN*	Paxilin	0.33	0.00
*RAB25*	Rab25	0.17	0.00
*RAD50*	Rad50	0.26	0.00
*RAD51*	Rad51	0.14	0.02
*RAF1*	C-Raf	0.32	0.00
*RB1*	Rb	0.11	0.07
*RPS6KB1*	p70S6K	0.70	0.00
*SMAD1*	Smad1	0.57	0.00
*SMAD3*	Smad3	0.57	0.00
*SMAD4*	Smad4	0.05	0.42
*SNAI1*	Snail	0.03	0.58
*SRC*	Src	0.54	0.00
*STAT5A*	STAT5-alpha	0.54	0.00
*STMN1*	Stathmin	−0.01	0.83
*SYK*	Syk	0.49	0.00
*TP53*	p53	0.11	0.07
*TP53BP1*	53BP1	0.49	0.00
*TSC2*	Tuberin	0.25	0.00
*VASP*	VASP	0.40	0.00
*XIAP*	XIAP	0.27	0.00
*XRCC1*	XRCC1	0.35	0.00
*YAP1*	YAP	0.47	0.00
*YBX1*	YB-1	0.14	0.02
*YWHAE*	14-3-3 epsilon	−0.03	0.60

### The combined effect of mRNA and miRNA on protein expression

**Figure 2:**
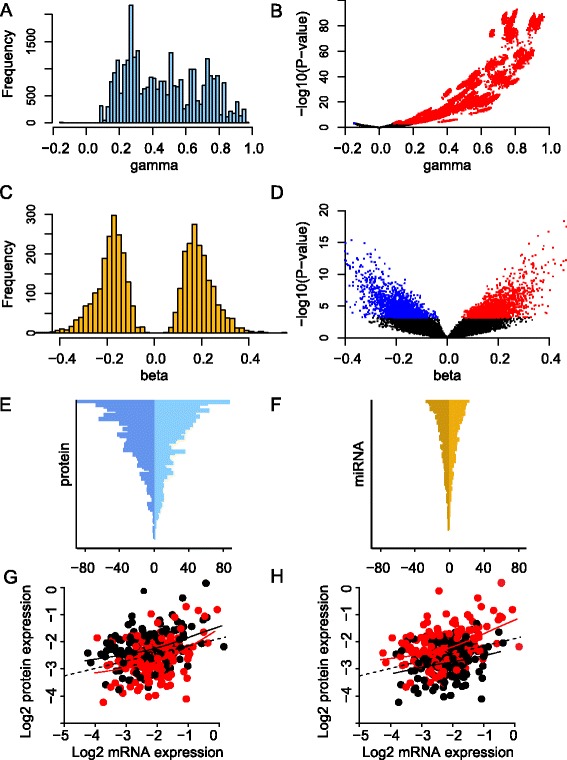
**Effect of mRNA and miRNA on protein expression. (A)** The effect of mRNA on protein for all significant mRNA coefficients (‘gamma’). **(B)** Volcano plot showing all estimated mRNA coefficients (‘gamma’) plotted against corresponding *P*-values. Significant and negative associations are shown in blue and significant and positive associations are shown in red. **(C)** The effect of miRNA on protein for all significant miRNA coefficients (‘beta’). **(D)** Volcano plot showing all estimated miRNA coefficients (‘beta’) plotted against corresponding *P*-values. Coloring as in **(B)**. **(E)** Number of miRNAs per protein. The horizontal axis represents the negative and positive number of associations with miRNAs, and the vertical axis represents the 105 proteins in descending order. **(F)** Number of proteins per miRNA. The horizontal axis represents the negative and positive number of associations with proteins, and the vertical axis represents the 421 miRNAs in descending order. **(G)** Example of a negative association between miRNA expression and protein expression. The horizontal axis represents *BRAF* mRNA expression and the vertical axis B-Raf protein expression (both on log_2_-scale). Each point represents a patient, and the color indicates whether the expression of miR-638 is above the median (red) or below the median (black). Solid lines represent smoothing splines fitted to the data. The dotted line represents a linear regression fit to the data. For any fixed level of mRNA expression, high expression of miR-638 is associated with decreased protein expression of B-Raf. **(H)** Example of a positive association between miRNA expression and protein expression. The horizontal and vertical axes are the same as in **(G)**, but here each patient point is color-coded according to miR-107 expression (red, miR-107 expression above median; black, miR-107 expression below median). For any fixed level of mRNA expression, high expression of miR-107 is associated with increased B-Raf protein expression.

The correlation analysis of the relationship between mRNA and protein expression presented above ignores any additional factors that may leverage how efficiently mRNA is translated into protein or how fast protein is degraded. Here, this was accomplished by allowing the intercept of the above linear model to depend on the expression of one or more miRNAs. Considering first the effect of one miRNA at a time, model 3 (see Methods) was fitted to the Oslo2 data for each combination of protein and miRNA, resulting in a total of 105 × 421 regression models (see Additional file 4A-F for coefficient estimates and *P*-values). Almost all significant (FDR <0.01) mRNA coefficients were positive, while the significant miRNA coefficients followed a bimodal distribution (Figure 2A-D). Among all the tested miRNA-protein associations, there were 3,687 (8.3%) significant ones, of which 1,882 were negative and 1,805 were positive (Additional file 4G). The overall structure of significant associations was confirmed by similar analysis performed in two additional data sets (see the ‘Replication in two independent data sets’ section below). For most miRNAs and proteins, the significant associations were a mixture of positive and negative effects (see Figure 2E-F for an overview and Figure 2G-H for specific examples). The proteins with the highest number of associations with miRNAs were B-Raf (n = 168), YB-1 (n = 145), Collagen VI (n = 106), cIAP (n = 103) and Syk (n = 102). Of the 105 studied proteins, 98 had at least one significant association with a miRNA (Additional file 4H). The number of significant connections to miRNAs was not associated with process, cellular location, functional type of protein or network centrality as quantified by a protein-protein interaction score [22,23] (Additional files 4I and 7). The miRNAs with the highest number of protein associations were miR-139-5p (n = 39), miR-497 (n = 38), miR-720 (n = 38) and miR-125b (n = 37), and 365 miRNAs (87%) had at least one significant association with a protein (Additional file 4J).

**Figure 3:**
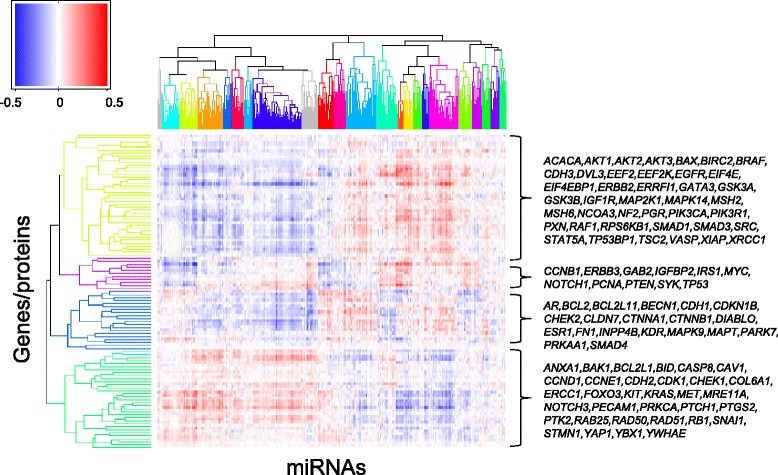
**The miRNA-mRNA-protein interactome.** The clustered heatmap represents all miRNA coefficients from the univariate model 3 with the 421 miRNAs shown as columns and the 105 gene/protein pairs shown as rows. Pearson correlation distance and complete linkage was used in the hierarchical clustering. The colors of the dendrograms represent the different clusters found by the PART algorithm [21]. The miRNAs form 23 unique clusters and the gene-protein pairs form four clusters. Genes/proteins residing in each cluster are indicated to the right in alphabetical order.

Two-way hierarchical clustering of all estimated miRNA coefficients, henceforth referred to as the interactome map, revealed groups of proteins with similar pattern of connectivity to miRNAs, and groups of miRNAs with similar pattern of connectivity to proteins (Figure 3). A subsequent cluster identification with PART [21] indicated the existence of four protein and 23 miRNA clusters (given in Additional file 4I-J). According to TargetScan [24], all miRNAs studied here (n = 421) have at least one representative of a total of 268 families; of these, 13 families have three or more representatives. There was a tendency for miRNAs in the same family to fall in the same cluster, most notably for the let-7, miR-17, miR-34 and miR-320 families (Additional file 4K).

### Direct interactions between miRNAs and mRNAs

An approximate estimate for the number of direct miRNA-mRNA interaction pairs among the negative and significant associations may be obtained by considering the number of *in silico* predicted targets among these associations. Among the 1,882 negative and significant associations, 290 were predicted by miRanda [25], 59 by TargetScan [24] and 19 by PicTar [26], and 50 associations were predicted by at least two of the three algorithms, including 29 unique proteins and 38 unique miRNAs (Additional file 8). Five of these have been functionally validated in previous studies; miR-19a targeting *CCND1*, miR-125b targeting *ERBB3*, miR-141 targeting *YAP1*, miR-222 targeting *BCL2L11* and miR-497 targeting *MAP2K1* (Additional file 4M).

### Combined effect of multiple miRNAs

So far the investigation has sought to explain protein expression in terms of the expression of mRNA and a single miRNA at a time. We may also consider the simultaneous effect of multiple miRNAs, which leads to the multivariate model 4 (see Methods). The coefficients obtained by fitting model 4 for each protein separately, in each case considering only the significant miRNAs from the univariate analysis, are found in Additional file 4N-P. Comparison of the results from the univariate and multivariate analyses was in overall agreement but also revealed differences (Additional file 9), consistent with the presence of groups of miRNAs connected in a similar manner to the proteins in the panel. In such cases, the multivariate model will tend to select only a few representative miRNAs to obtain the best possible predictions and avoid overfitting, while the univariate models produce estimates that are optimal when information about other miRNAs is ignored.

### Association with clinical parameters

**Figure 4:**
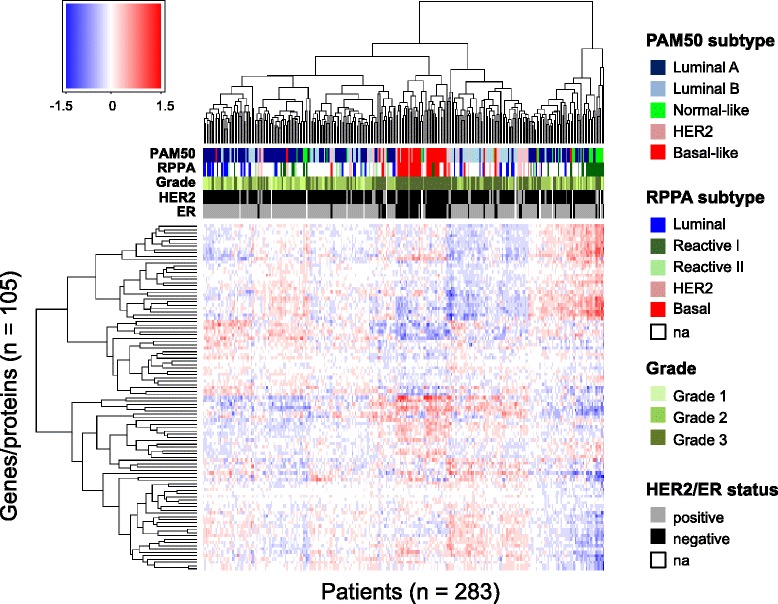
**Patient-specific predicted effects of miRNA on protein.** Rows represent the 105 genes/proteins and columns represent the 283 patients. The color bars under the dendrogram represent PAM50 and RPPA molecular subtypes (mRNA and protein based, respectively), histological grade, human epidermal growth factor receptor 2 (HER2) status, and estrogen receptor (ER) status. The colors in the heatmap represent the patient-specific effects of miRNA on protein and are numerical values obtained by multiplying each miRNA coefficient (from the multivariate analysis) with the corresponding miRNA expression, in a given patient, for a given protein. The clustering of the proteins and patients was performed using Euclidean distance and complete linkage. *Na*, not available.

For each patient and each protein, a score based on the observed miRNA levels in a patient can be computed that reflects the total effect of all miRNAs on protein expression. The resulting table of scores (one for each combination of protein and patient) is visualized as a heatmap in Figure 4. Noteworthy, using these scores one can recapitulate a cluster consisting primarily of estrogen receptor (ER)-negative samples of high grade, classified as basal-like subtype by both PAM50 [27] and RPPA [18], suggesting distinct miRNA effects on protein in this type of breast cancer. Luminal A and luminal B tumors clustered quite separately from each other, with one luminal A cluster containing most of the luminal tumors from the RPPA subtype classification. The correlation between HER2 status and cluster assignment was not significant.

### Replication in two independent data sets

To further investigate the robustness of the model and the estimated parameters of the primary data set (Oslo2), similar analyses were performed on two additional breast cancer data sets. The first (TCGA) has a similar distribution of ER-status, HER2-status and PAM50 subtypes as the Oslo2 data set, while the second (DBCG) has a higher fraction of ER-negative and HER2-positive tumors of the basal-like and HER2-enriched subtypes according to the mRNA expression-based PAM50 classification (Additional file 10). Altogether, 348 miRNAs and 34 proteins were present in all three data sets. An overall comparison of the effects of individual miRNAs and corresponding *P*-values showed consistency across the data sets (Additional file 11). A comparison of all miRNA coefficients obtained (using the univariate model) for each of the three data sets and for each protein individually (Additional files 12 and 13) revealed a large degree of consistency between pairs of data sets, with some exceptions: *ERBB2* and *PIK3CA* had negative correlation between Oslo2 and DBCG coefficients, and seven additional genes (*BCL2*, *CCNE1*, *ESR1*, *MAP2K1*, *MAPK14*, *PECAM1* and *SRC*) had negative correlation between Oslo2 and TCGA coefficients.

As a final step of validation, the multivariate model 4 was fitted to the Oslo2 data set and the estimated coefficients were used to predict protein expression for individual patients in the DBCG and TCGA data sets, using mRNA and miRNA expression values from the respective patients as predictors. Only proteins and significant miRNAs from the multivariate analysis present in all three data sets were considered in this analysis. It was found that for 19 of the 34 proteins considered (56%), the correlation between predicted and actual protein expression was significant (*P* < 0.05) in both data sets (Additional files 14 and 15). The genes for which the predicted and measured protein levels were most strongly correlated included *ESR1*, *CCNB1*, *CCNE1*, *PGR*, *RPS6KB1* and *KIT* (all with Pearson correlations >0.60). A complete list of miRNAs associated with the expression of these proteins is provided in Additional file 4O.

### Functional validation *in vitro*

**Figure 5:**
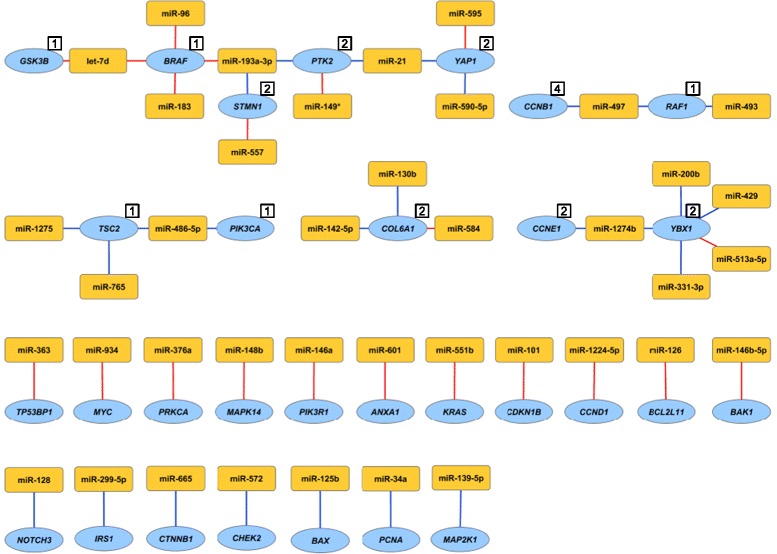
**miRNA-protein associations validated by cell line functional assessment.** Individual miRNAs were overexpressed in the MDA-MB-231 breast cancer cell line and the effect on protein expression was assessed. Shown are miRNA-protein associations that were both estimated based on the Oslo2 patient data and confirmed with the *in vitro* cell line experiment. The numbers in the white boxes represent protein cluster number in cases where several proteins share miRNA associations. Red lines indicate positive associations and blue lines negative associations. Yellow nodes represent miRNAs and blue nodes represent genes/proteins. The figure was made using Cytoscape version 2.8.3 [28].

To functionally assess the miRNA-protein associations *in vitro,* single miRNAs were overexpressed in the MDA-MB-231 breast cancer cell line and the effect on protein expression was investigated. Cell line experimental data were available for 416 miRNAs and 76 proteins (Additional file 4L). In all, 47 miRNA-protein associations found in Oslo2 were significantly and consistently functionally validated in the breast cancer cell line (Figure 5), 21 representing positive associations between miRNA and protein and 26 representing negative associations. Proteins sharing miRNAs in the same direction among the functionally validated miRNA-protein associations were in most cases found to be clustering together in the interactome heatmap (Figure 3).

### Consistently strong miRNA-protein associations across three data sets

**Figure 6:**
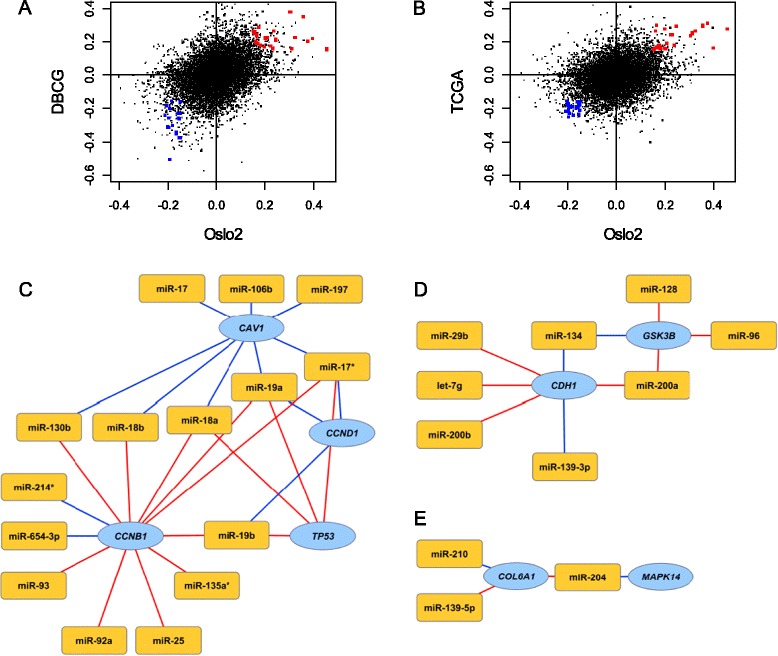
**Comparison of miRNA-protein associations in three independent data sets. (A)** Scatterplot representing the miRNA coefficients in model 3 estimated in the Oslo2 and DBCG data sets. **(B)** Scatterplot representing the beta values estimated in the Oslo2 and TCGA data sets. In **(A)** and **(B)**, red points indicate miRNA-protein associations with a miRNA coefficient above 0.15 across all three data sets and blue points indicate miRNA-protein associations which have a miRNA coefficient below −0.15 across all three data sets. **(C-E)** miRNA-protein associations with miRNA coefficients exceeding 0.15 in absolute value in all three data sets. Red lines indicate positive associations and blue lines negative associations. Yellow nodes represent miRNAs and blue nodes represent proteins. Panels **(C-E)** were made using Cytoscape version 2.8.3 [28].

To identify consistently strong positive or negative miRNA-protein associations across all three data sets, all associations with a coefficient value exceeding 0.15 in absolute value were selected (Figure 6A,B). This resulted in 41 miRNA-protein relationships, representing 8 unique proteins and 26 unique miRNAs (Figure 6C-E). The highest number of high concurrence associations was found for *CCNB1* with 12 miRNA-protein associations, of which 2 were negative and 10 positive (Figure 6C). Among the positive associations, seven miRNAs (miR-18a, miR-18b, miR-19a, miR-19b, miR-25, miR-92a and miR-93) involved the oncogenic miR-17 family [29]. The gene *CDH1*, which encodes the E-cadherin protein, shared two miRNA associations with *GSK3B* across all three data sets: a positive association with miR-200a and a negative association with miR-134 (Figure 6D). These proteins are negatively associated with the oncogenic process of epithelial-mesenchymal transition (EMT) [30], suggesting a coordinate miRNA effect on a single cellular process. *COL6A1* and *MAPK14* were associated with miR-204 in the opposite direction across all three data sets, and *COL6A1* was additionally associated with miR-139-5p and miR-210 (Figure 6E).

## Discussion

### A comprehensive map of associations between miRNAs and proteins

A map has been derived describing the individual contributions of in*-cis* mRNA expression and whole-genome miRNA expression in predicting protein expression in breast cancer. The analysis is based on a novel integrative model derived from first principles: any change in protein expression level must derive from an influx of protein proportional to the mRNA expression level and an outflux (degradation) of protein proportional to the protein expression level. The model was found to be in reasonable agreement with the actual observations of mRNA-protein relationships. The ratio of the two proportionality factors defines the slope of the mRNA-protein response curve and is modeled as a function of one or more miRNAs. The univariate model 1 (which should strictly be called bivariate, since it involves two covariates) is used to assess the strength of association between a protein and one miRNA at a time, adjusting for the effect of in*-cis* mRNA expression. It is essential to correct *P*-values for the fact that 105 × 421 = 44,205 tests are performed in parallel, and in this study a rather strict criterion of FDR <0.01 was used. A total of 3,687 significant associations between individual miRNAs and proteins were found in the analysis of the Oslo2 data, and no more than 37 of these are expected to be false positives at the chosen level of significance. After proper adjustment of *P*-values, there is still a risk that clinical composition and other specifics of the patient material influence the analysis. To further assess the validity of the associations, similar analyses were therefore performed on two additional breast cancer data sets (DBCG and TCGA) for the proteins and miRNAs that were present in all three data sets. This analysis largely confirmed the validity of the interactome found on the basis of Oslo2.

### Two models for two different purposes

The univariate model is appropriate for studying the association between individual miRNAs and protein (ignoring the effect of all other miRNAs), and was therefore used to infer the miRNA-protein interactome in Oslo2, which was subsequently confirmed in the two validation data sets.

The multivariate model 2 is more appropriate for construction of a predictive model for protein expression, as it considers the combined effect of all miRNAs and thus weighs the contributions of the individual miRNAs to obtain optimal prediction. Fitting the multivariate model to one data set (Oslo2) and subsequently predicting protein expression in the two other data sets, more than half of the proteins that were present in all data sets resulted in significant predictions. It should be noted that prediction in this context is nontrivial since not all miRNAs that were used to derive the coefficients for Oslo2 are present in the two other data sets, and the three data sets have different clinical compositions. In addition, although the analysis performed in this study determines the statistical relationships among variables, miRNA regulation is ultimately a stoichiometric event where the absolute target-to-miRNA ratio matters [31]. Thus, functional experiments are essential to separate direct (or indirect) molecular interactions from other forms of coordination that may involve other regulatory mechanisms. In this study, the main focus has been to capture the general pattern of all forms of coordination.

### Associations reflect multiple mechanisms

Significant associations between miRNAs and proteins were almost evenly distributed between positive and negative values. Taking into account the strict significance criteria used and the reproducibility across three independent data sets, this suggests that direct inhibitory action of miRNAs on protein translation constitutes only a small part of all present associations. The low overlap found in a comparison with *in silico* predicted targets using three different target prediction algorithms supports this contention. Alternatively, this may reflect challenges in the ability of current algorithms to accurately predict miRNA and mRNA interactions. Significant associations may reflect direct and indirect contributions of miRNAs to protein expression, effects in the opposite direction (that is, from protein to miRNA), as well as coordinated expression attributable to factors not accounted for in the analysis and not involving a direct causal link between miRNA and protein. It is not possible in general to distinguish these mechanisms from each other on the basis of a single snapshot of the miRNA-mRNA-protein state of the cell, except in cases where additional evidence is available to confirm or strengthen specific hypotheses. It should be emphasized, however, that the overall association pattern was recapitulated across data sets, suggesting that they are robust and potentially mechanistic.

### Direct and indirect effects of miRNAs on proteins

Focusing on the direct and indirect effects of miRNAs on protein, the former involves physical binding of miRNA to mRNA, while the latter may represent proteins that are downstream of the direct target of the miRNA, where, for example, feedforward and feedback regulatory loops could be involved. Positive associations then reflect relations where increasing miRNA expression is coupled to increasing protein levels (and decreasing miRNA expression with decreasing protein levels). This could be exemplified by a feedforward loop where a miRNA downregulates the repressor of a protein. On the other hand, the identified negative associations represent inverse relations between miRNA expression and protein output. Such relations could be direct miRNA-mRNA target interactions, or, for example, negative feedback loops. The involvement of miRNAs in feedback and feedforward loops have received attention in the literature [32,33].

### *In silico* and *in vitro* model validation

The significant negative associations were matched with *in silico* target predictions to identify putative direct miRNA-mRNA interactions. Due to the limited accuracy of such predictions [34], at least two out of three algorithms were required to predict a target. Some of the candidates for direct interaction have been previously validated. Furthermore, a subset of the estimated miRNA-protein associations was functionally validated in the MDA-MB-231 cell line, including four associations that were *in silico* predicted. The fact that only a small subset of all associations were functionally validated may partly be explained by the difficulty of capturing indirect effects in an *in vitro* cell line experiment due to, for example, context dependence, here exemplified by a triple-negative cell line. The small number of functionally validated associations may also suggest that many estimated associations represent extensive coordination between miRNAs and proteins, but that the direct causal effect of miRNAs on proteins is limited compared with the total number of associations. Further experimental testing where groups of miRNAs are combined is needed to further investigate this.

### Protein predictions in individual patients may have clinical impact

The interactome map showing the effect of miRNA on protein output is a map delineating possible associations between miRNAs and proteins, and was discovered by integration of three different data levels. The exploitation of these connections, however, is patient specific. Interestingly, the ER-negative patients with high grade breast cancer disease clustered together, suggesting that differences in miRNA expression affect protein levels and ultimately breast cancer phenotype. Alternatively, the intrinsic miRNA expression pattern may vary between different types of breast cancer, resulting in differing activation of various signaling networks. In agreement with this, ER-positive and ER-negative patients have previously been shown to have differential miRNA expression signatures [2], and differential expression of miRNAs between tumors of different subtype and grade has also been reported [2,17,35].

### miRNA-protein associations are consistent across three data sets

Comparison of results from different data sets involving different measurement platforms is potentially challenging [36,37]. While platform-specific bias is (in the ideal case) reduced by proper pre-normalization of the data, one would expect some differences between studies performed on different platforms to remain. Nevertheless, consistency between the three data sets was overall high. The differences that were found may partly be ascribed to the differences in clinical composition as the balance between HER2- and ER-positive/negative status was different between the data sets, and expression of these proteins is directly associated with these clinical markers (for example, *PIK3CA* is often mutated in ER-positive tumors [18,38]). The inconsistencies of miRNA coefficients found between Oslo2 and DBCG for miRNA species associated with key proteins such as HER2 and PIK3CA may be caused by the higher fraction of HER2-positive and ER-negative tumors in DBCG compared with Oslo2. *TP53* and *CCNB1* were among the most highly correlated genes across the data sets, highlighting these predictions of miRNA-protein correlations as the most robust.

One of the miRNA-protein association networks that was consistently found across all three data sets has c-Myc as a potential underlying common denominator; c-Myc is a transcription factor for both *CCNB1* [39] and the miR-17 family of miRNAs [40], which may explain this positive association and may suggest an oncogenic functional link between *CCNB1*, the miR-17 family and increased proliferation. This observation exemplifies another reason why the frequency of positive associations between miRNA and protein may be more frequent than expected in that it could represent co-regulation of the miRNA and the protein rather than regulation of translation of the protein by the miRNA. In addition, *TP53*, which was positively associated with three miR-17 family members, namely miR-18a, miR-19a and miR-19b, has been shown to be a transcriptionally activated target gene of c-Myc [39]. Interestingly, c-Myc has been shown to be a transcriptional repressor of *CAV1* and *CCND1* [39], and these two genes were here shown to be negatively associated to several of the miR-17 family miRNAs, including miR-17, miR-18a, miR-18b, miR-19a, miR-19b and miR-106b (Figure 6C). One of these negative associations, *CCND1* with miR-19a, was among the *in silico* predicted interactions (Additional file 8), and *CCND1* has previously been functionally validated as a target gene of both miR-19a [41] and miR-19b [42].

*CDH1* and *GSK3B* are negatively associated with the tumorigenic EMT process [30]. *GSK3B* is known to be a transcriptional inhibitor of the E-cadherin transcriptional repressor Snail [43]. The miR-200 family can also revert EMT by targeting two other E-cadherin transcriptional repressors, namely *ZEB1* and *ZEB2* [44]. Thus, the shared associations between *CDH1* and *GSK3B* with miR-200a, in addition to the association of *CDH1* with miR-200b (Figure 6D), likely reflect an EMT-associated network. The two other miRNAs that *CDH1* was positively associated with, let-7 g and miR-29b, have previously been shown to target extracellular matrix proteins such as collagens [45,46]. Furthermore, the negative associations between miR-134 and *CDH1* and *GSK3B* are both *in silico* predicted by the miRanda algorithm [25]. Thus, miR-134 may potentially directly bind to *CDH1* and *GSK3B* mRNA and downregulate expression. Further experimental studies are needed to confirm this, but if true, this would be an example of how miRNAs may effectively target several genes in the same pathway. Interestingly, overexpression of miR-134 was previously found to promote EMT while knockdown inhibited EMT [47], which further strengthen this potential functional relationship.

## Conclusions

We have provided a genome-wide description of the associations between miRNA expression and protein expression for a selected panel of 105 cancer-associated proteins. This landscape of interaction between miRNAs and proteins reveals several intriguing relationships, including the fact that groups of miRNAs coordinately interact with groups of proteins, hence suggesting ‘block interaction’ as a mode of modulation of protein modules in breast cancer. Studies of the effects of miRNAs on protein expression, through mRNA regulation, increase our understanding of the role of miRNAs in breast cancer. Our model predicted both previously demonstrated miRNA-protein associations and new candidate miRNA targets, and showed overall consistent miRNA-protein associations across three independent data sets. Furthermore, it allows a more accurate description of the actual phenotypic effects of miRNAs as it is comprehensively capturing both direct and indirect effects. This may prove important to elucidate the biological role of miRNAs, in particular when considering the role of miRNAs at the network level.

## Additional files

Additional file 1:
**Two mechanisms for miRNA regulation of protein expression.** The top panel depicts the relation between mRNA expression and protein expression (i.e. the mRNA-protein response curve) when the miRNA is not expressed. The lower left panel shows the effect of mRNA destabilization: the resulting loss in mRNA expression leads to reduced protein expression, while the mRNA-protein response curve remains the same. The lower right panel shows the effect of translation repression: in this case the mRNA expression remains unaffected, but the change in the mRNA-protein response curve leads to reduced protein expression. Additional file 2:
**A schematic outline of the approach used to assess the effect of miRNAs on protein expression.**
Additional file 3:
**Supplementary Methods.**
Additional file 4:
**Supplementary Data: Coefficient estimates, P-values, significant miRNA-protein associations, annotation of proteins and miRNAs, cell line functional data,** ***in silico*** **predictions and RPPA data.** **A**, estimated mRNA coefficients (univariate analysis). **B**, P-values for estimated mRNA coefficients (univariate analysis). **C**, estimated miRNA coefficients (univariate analysis). **D**, P-values for estimated miRNA coefficients (univariate analysis). **E**, estimated constants (univariate analysis). **F**, P-values for estimated constants (univariate analysis). **G**, significant miRNA-protein pairs. **H**, number of miRNAs per protein. **I**, annotation of proteins. **J**, number of proteins per miRNA. **K**, location of miRNA family members in respect to miRNA clusters. **L**, standard scores from miRNA library screen by RPPA of the MDA-MB-231 cell line. **M**, significant and negative miRNA-protein associations *in silico* predicted in 2 out of 3 algorithms tested. **N**, estimated mRNA coefficients (multivariate analysis). **O**, estimated miRNA coefficients (multivariate analysis). **P**, estimated constants (multivariate analysis). **Q**, log2-transformed and median centered RPPA (protein) data for the Oslo2 cohort. Additional file 5:
**Distribution of mRNA-protein correlations for the 105 proteins in Oslo2.** The dashed line shows the fit found with a kernel density estimator with Gaussian kernel. Additional file 6:
**Scatterplots of mRNA and protein expression for all 105 proteins.** Each dot represents a patient and the colors indicate PAM50 molecular subtype (red: basal-like; pink: HER2-enriched; green: normal-like; dark blue: luminal A; light blue: luminal B). Additional file 7:
**Number of associated miRNAs in relation to protein type.** **A**, boxplots showing the distribution of the number of miRNAs per protein associated process group. The number in parentheses shows the number of proteins in each group. *TF*: Transcription factor; *ECM*: extracellular matrix. **B**, boxplots showing the distribution of the number of miRNAs when proteins are divided into cellular location. **C**, boxplots showing the distribution of the number of miRNAs when proteins are divided into functional type. **D**, protein-protein interaction (ppi) degree (score) versus number of associated miRNAs per protein. The line indicates the least squares fit to the data. See Additional file 4I for details on the protein annotation. Additional file 8:
**Potential direct miRNA-mRNA target interactions identified from the univariate model.** The depicted interactions represent significant, negative associations identified from the univariate model that were also *in silico* predicted. Yellow nodes represent miRNAs and blue nodes represent proteins. Three miRNA target algorithms were used to assess potential direct interactions; TargetScan [24], miRanda [25] and PicTar [26]. The full edges indicate previously validated interactions (see Additional file 4 M), and dashed edges indicate potential direct interactions. Black edges represent interactions predicted by at least two out of three algorithms, and purple edges represent interactions predicted by all three algorithms. The thickness of the edges represents relative beta values. The figure was made using Cytoscape version 2.8.3 [28]. Additional file 9:
**Multivariate versus univariate miRNA coefficients.** The plot shows the univariate miRNA regression coefficients (“beta”) on the x-axis and the multivariate coefficients on the y-axis. Only those coefficients are shown that are significant in the univariate analysis (FDR < 0.01) and 0 in the multivariate analysis. Additional file 10:
**Clinical composition of the Oslo2, DBCG and TCGA breast cancer data sets.** The histograms are comparing ER and HER2 status together with molecular subtypes derived using the PAM50 calling. *Neg*: negative; *pos*: positive; LumA: Luminal A; LumB: Luminal B. Additional file 11:
**Comparison of estimated values across three data sets.** (i) Comparison of estimated beta values between the Oslo2 and DBCG cohort, and (ii) between the Oslo2 and TCGA cohort. (iii) Comparison of Z-score transformed P-values between the Oslo2 and DBCG cohort, (iv) and between the Oslo2 and TCGA cohort. Z-scores were obtained by applying the inverse cumulative normal distribution function to the P-values, and they follow a standard normal distribution under the null hypothesis of no effects of miRNA on protein expression. The grey lines indicate the first principal component curve of the data. Additional file 12:
**Comparison of estimated beta values across data sets.** The x-axis represents the Pearson correlation of beta values per protein between the Oslo2 and DBCG cohort and the y-axis represents the Pearson correlation of beta values per protein between the Oslo2 and TCGA cohort. Additional file 13:
**Scatterplots comparing estimated beta values from Oslo2 vs. DBCG and Oslo2 vs. TCGA.** Each dot represents a miRNA and the x-axes represent estimated beta values for the Oslo2 cohort from the univariate analysis. The y-axes represent estimated beta values for the DBCG and TCGA data sets, respectively, from the univariate analysis. The dashed lines indicate the least squares fit to the data and the red line indicates the first principal component curve of the data. Pearson correlation is indicated in the corner of each plot. Additional file 14:
**Comparison of predicted versus measured protein in the DBCG and TCGA data sets.** To calculate predicted protein expression, the estimated mRNA and miRNA coefficients from the Oslo2 cohort were fitted into equation (4) using the actual miRNA and mRNA expression data of the DBCG and TCGA data sets, respectively. Then the predicted and the measured protein expression were compared using correlation. The x- and y-axis represent Pearson correlation. Additional file 15:
**Measured versus predicted protein in the DBCG and TCGA data sets.** The multivariate model was fitted to the Oslo2 data set and used to predict protein expression for individual patients in the DBCG and TCGA data sets, using mRNA and miRNA expression values from the respective patients as predictors. The table shows an overview of the correlation between the predicted and the measured protein expression including correlation P-values for the 34 proteins overlapping all three cohorts. The following scatterplots shows the predicted versus the measured protein expression in the DBCG and the TCGA data sets. Solid lines represent a smoothing spline fitted to the data. The Pearson correlation between the predicted versus the measured protein expression is indicated above each plot.

## Abbreviations

DBCG: Danish Breast Cancer Cooperative Group
EMT: epithelial-mesenchymal transition
ER: estrogen receptor
FDR: false discovery rate
miRNA: microRNA
PART: Partitioning Algorithm using Recursive Thresholding
RPPA: reverse-phase protein array
TCGA: The Cancer Genome Atlas
