# Supplementary figures and images for: Integrated analysis reveals microRNA networks coordinately expressed with key proteins in breast cancer
Source: Genome Med. 2015 Feb 2;7(1):21. doi: 10.1186/s13073-015-0135-5 (PMC4396592; doi:10.1186/s13073-015-0135-5)

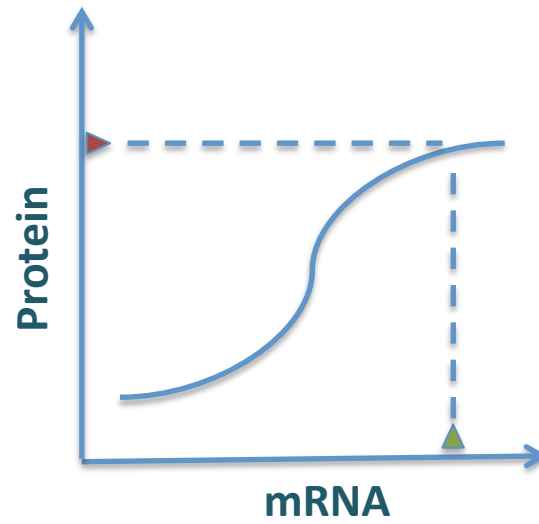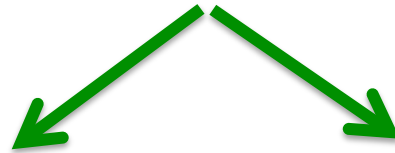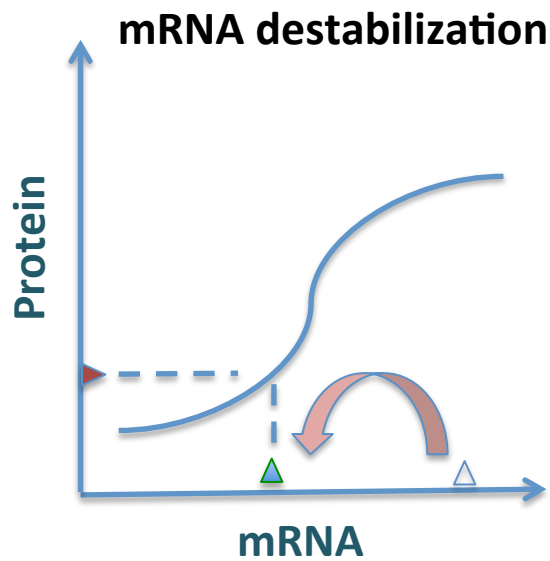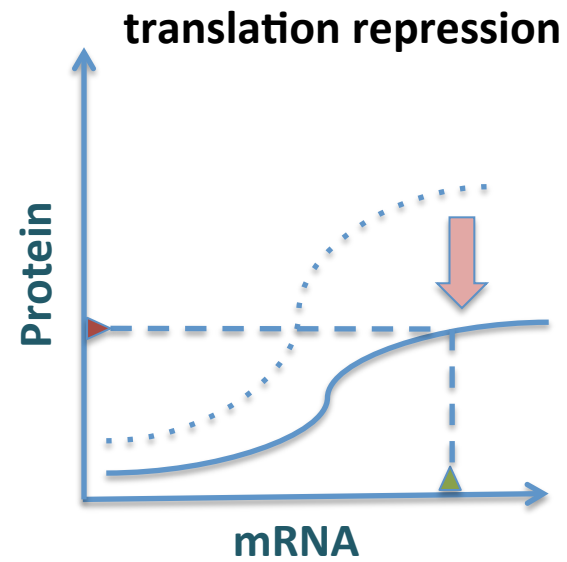

Supplement: Additional file 1: — Two mechanisms for miRNA regulation of protein expression. The top panel depicts the relation between mRNA expression and protein expression (i.e. the mRNA-protein response curve) when the miRNA is not expressed. The lower left panel shows the effect of mRNA destabilization: the resulting loss in mRNA expression leads to reduced protein expression, while the mRNA-protein response curve remains the same. The lower right panel shows the effect of translation repression: in this case the mRNA expression remains unaffected, but the change in the mRNA-protein response curve leads to reduced protein expression. [file 13073_2015_135_MOESM1_ESM.pdf]

# Correlation mRNA-protein

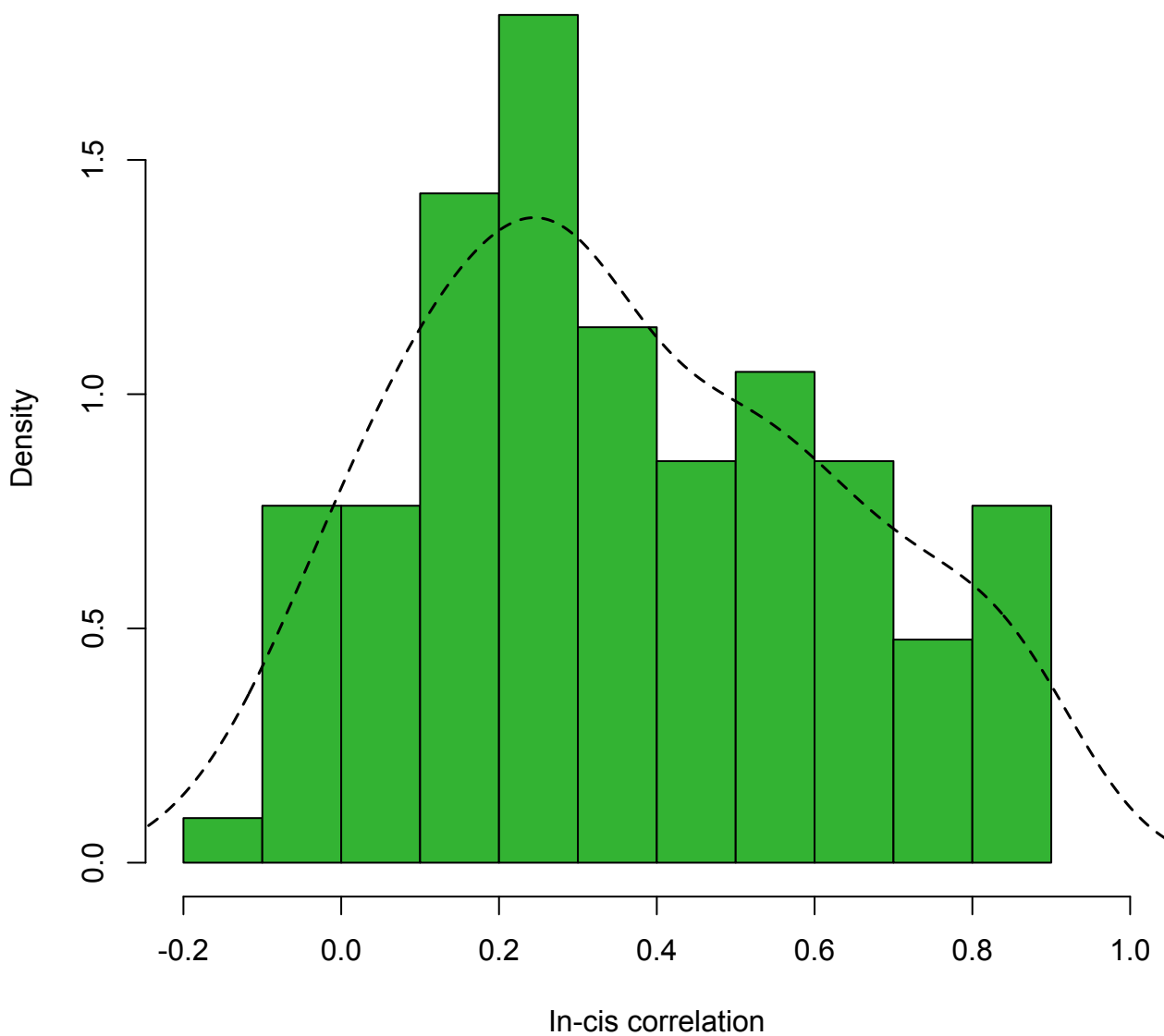

Supplement: Additional file 5: — Distribution of mRNA-protein correlations for the 105 proteins in Oslo2. The dashed line shows the fit found with a kernel density estimator with Gaussian kernel. [file 13073_2015_135_MOESM5_ESM.pdf]

A

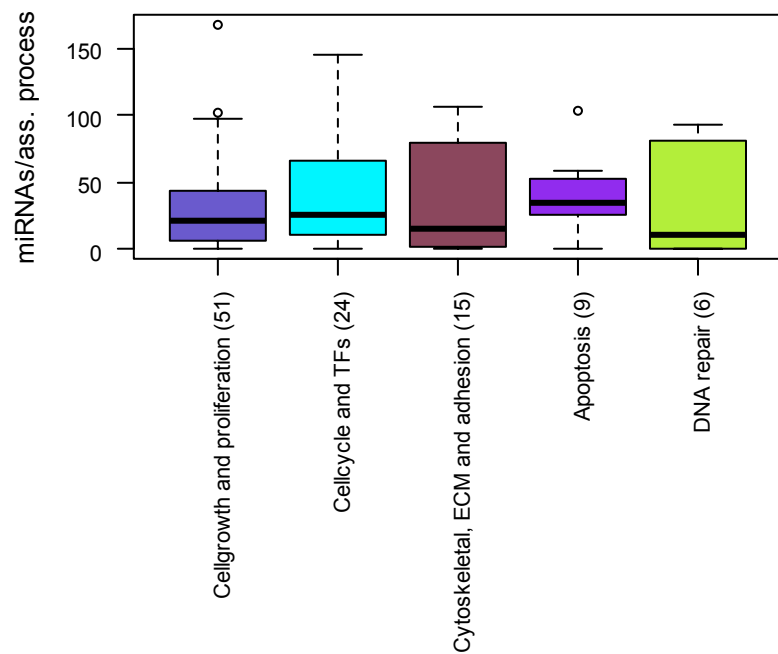

B

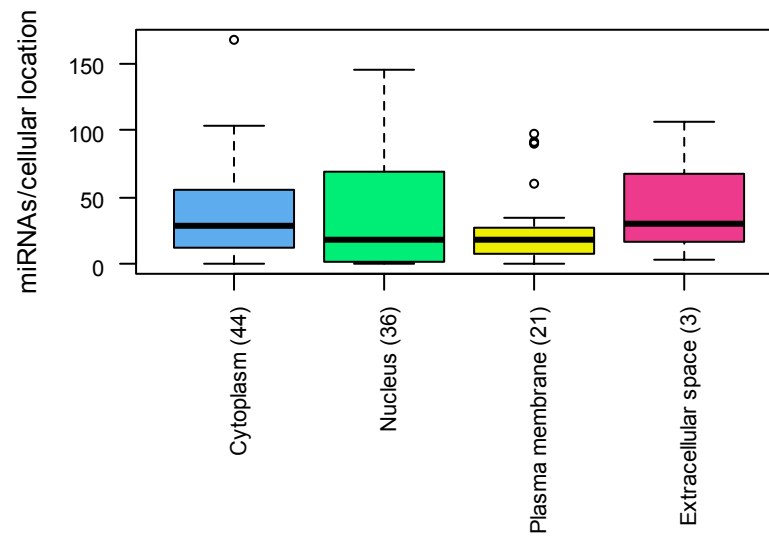

C

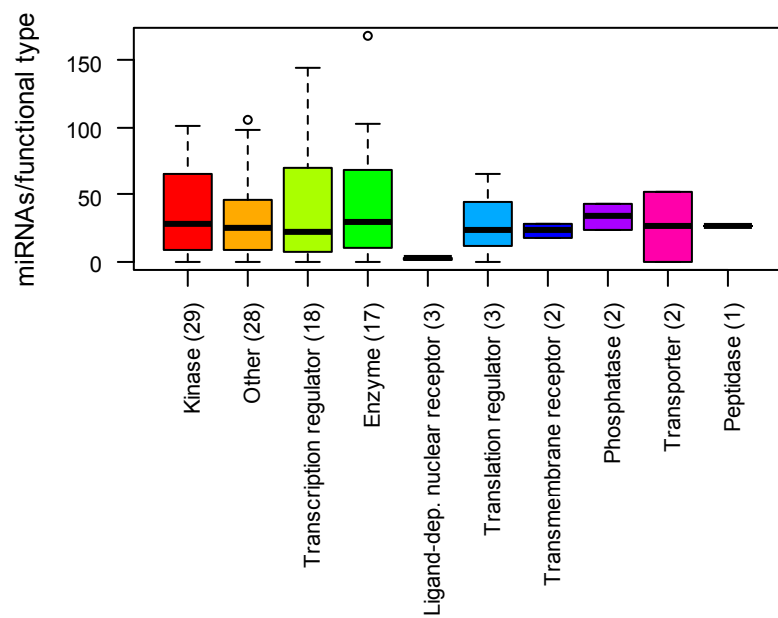

D

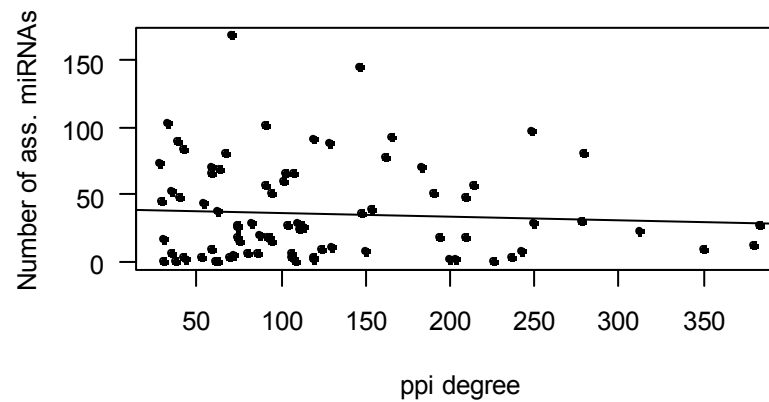

Supplement: Additional file 7: — Number of associated miRNAs in relation to protein type. A, boxplots showing the distribution of the number of miRNAs per protein associated process group. The number in parentheses shows the number of proteins in each group. TF: Transcription factor; ECM: extracellular matrix. B, boxplots showing the distribution of the number of miRNAs when proteins are divided into cellular location. C, boxplots showing the distribution of the number of miRNAs when proteins are divided into functional type. D, protein-protein interaction (ppi) degree (score) versus number of associated miRNAs per protein. The line indicates the least squares fit to the data. See Additional file 4I for details on the protein annotation. [file 13073_2015_135_MOESM7_ESM.pdf]

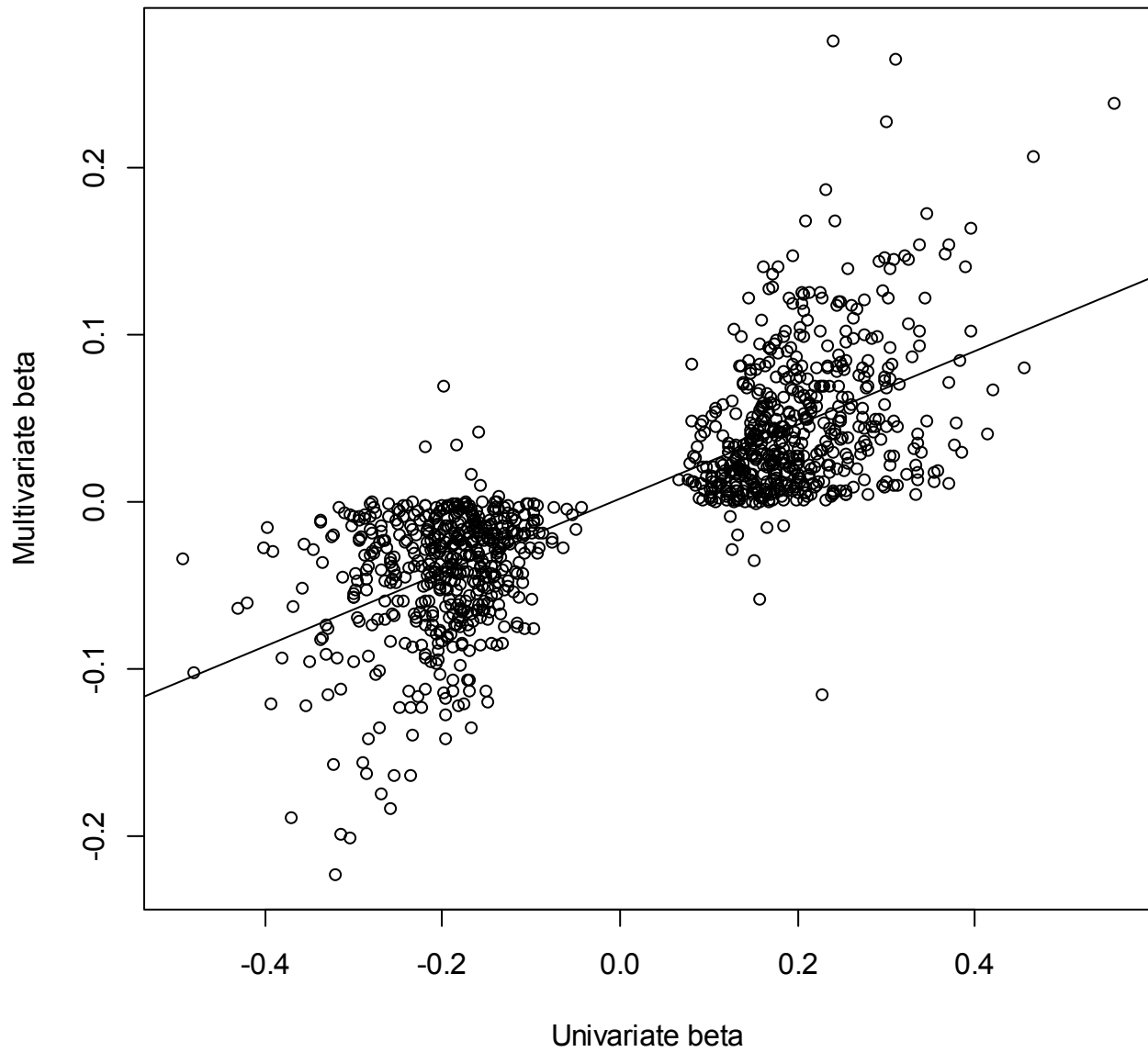

Supplement: Additional file 9: — Multivariate versus univariate miRNA coefficients. The plot shows the univariate miRNA regression coefficients (“beta”) on the x-axis and the multivariate coefficients on the y-axis. Only those coefficients are shown that are significant in the univariate analysis (FDR < 0.01) and 0 in the multivariate analysis. [file 13073_2015_135_MOESM9_ESM.pdf]

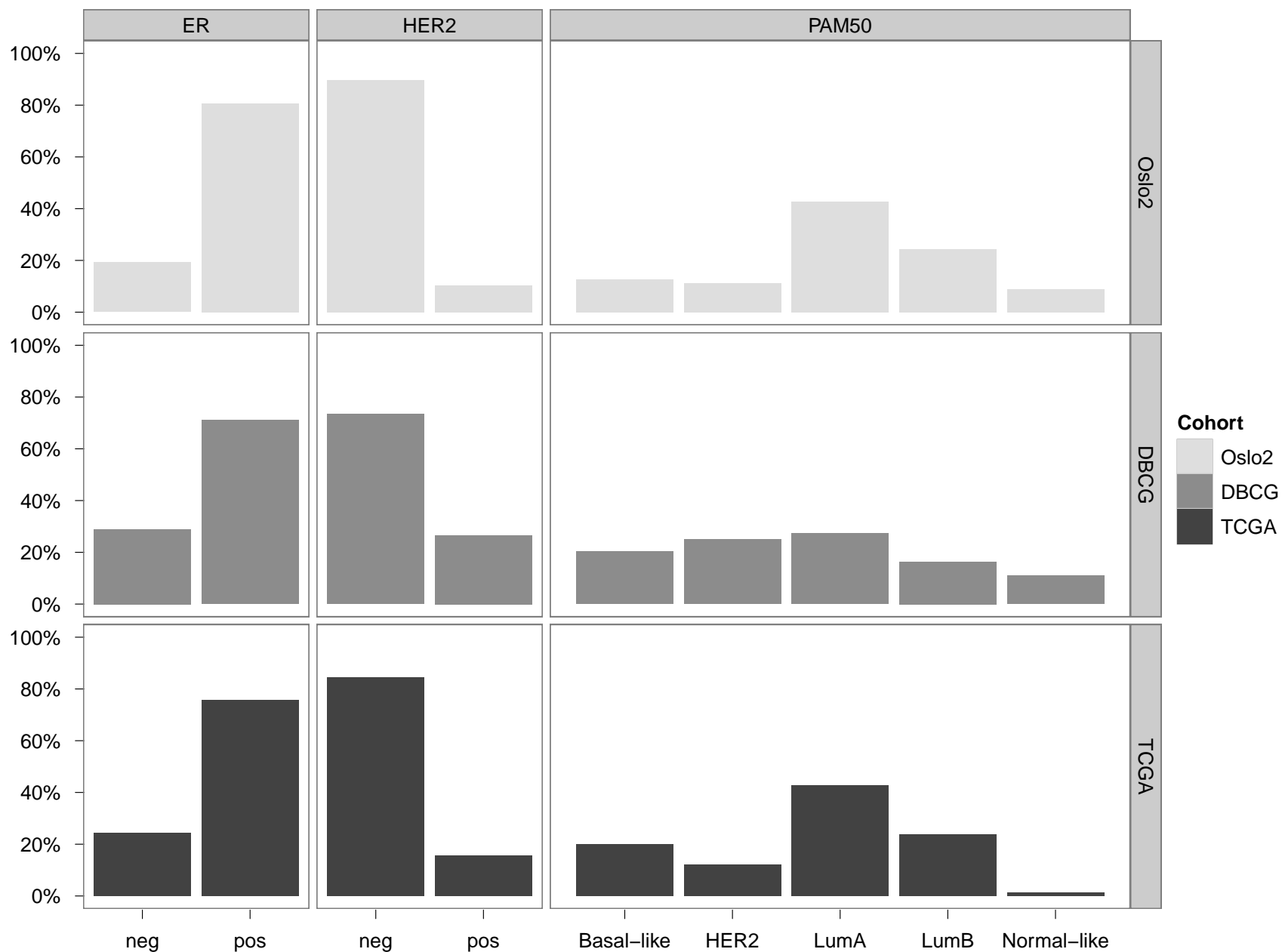

Supplement: Additional file 10: — Clinical composition of the Oslo2, DBCG and TCGA breast cancer data sets. The histograms are comparing ER and HER2 status together with molecular subtypes derived using the PAM50 calling. Neg: negative; pos: positive; LumA: Luminal A; LumB: Luminal B. [file 13073_2015_135_MOESM10_ESM.pdf]

Oslo2 - TCGA correlation

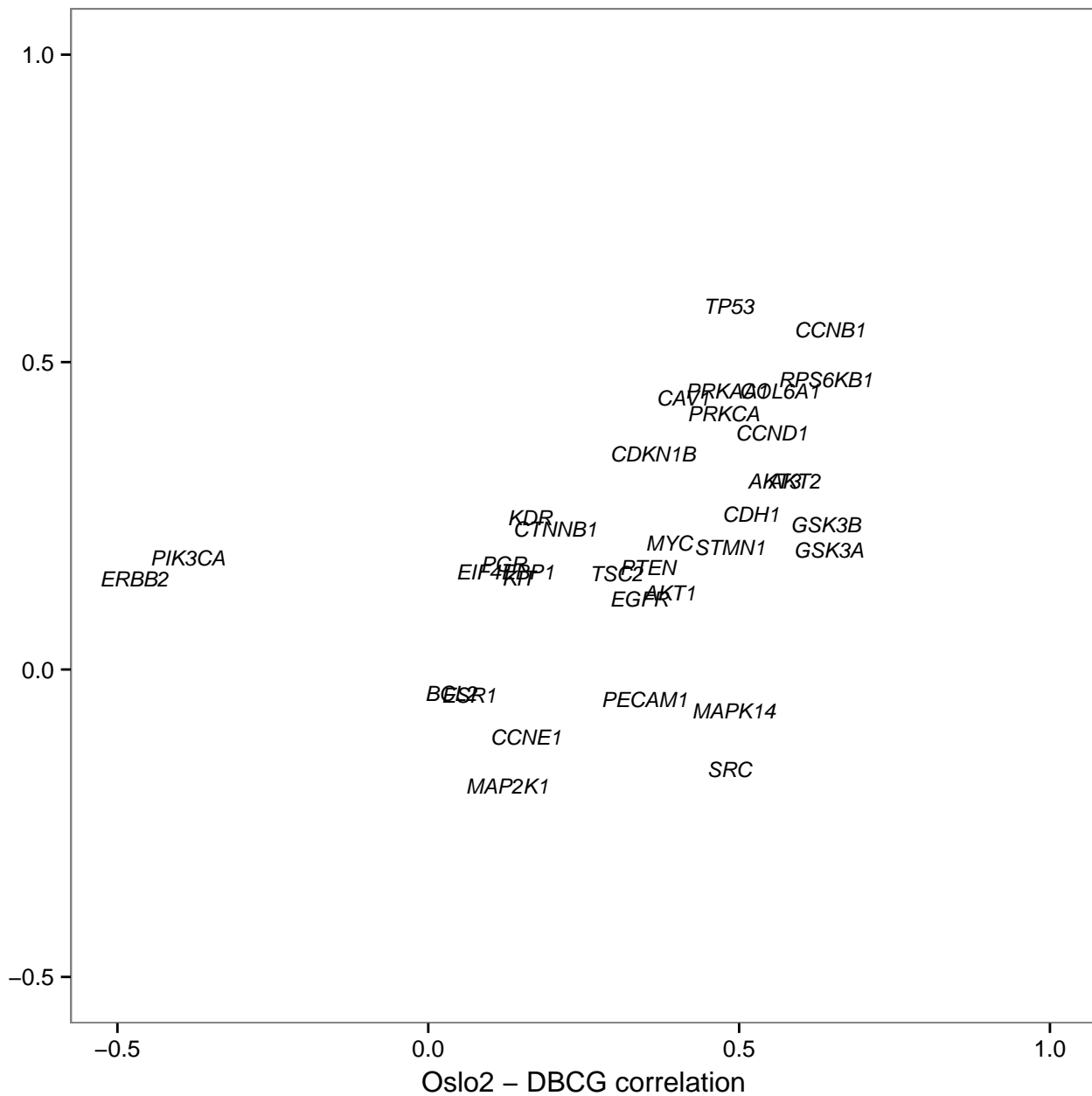

Oslo2 - DBCG correlation

Supplement: Additional file 12: — Comparison of estimated beta values across data sets. The x-axis represents the Pearson correlation of beta values per protein between the Oslo2 and DBCG cohort and the y-axis represents the Pearson correlation of beta values per protein between the Oslo2 and TCGA cohort. [file 13073_2015_135_MOESM12_ESM.pdf]

TCGA predicted vs measured protein correlation

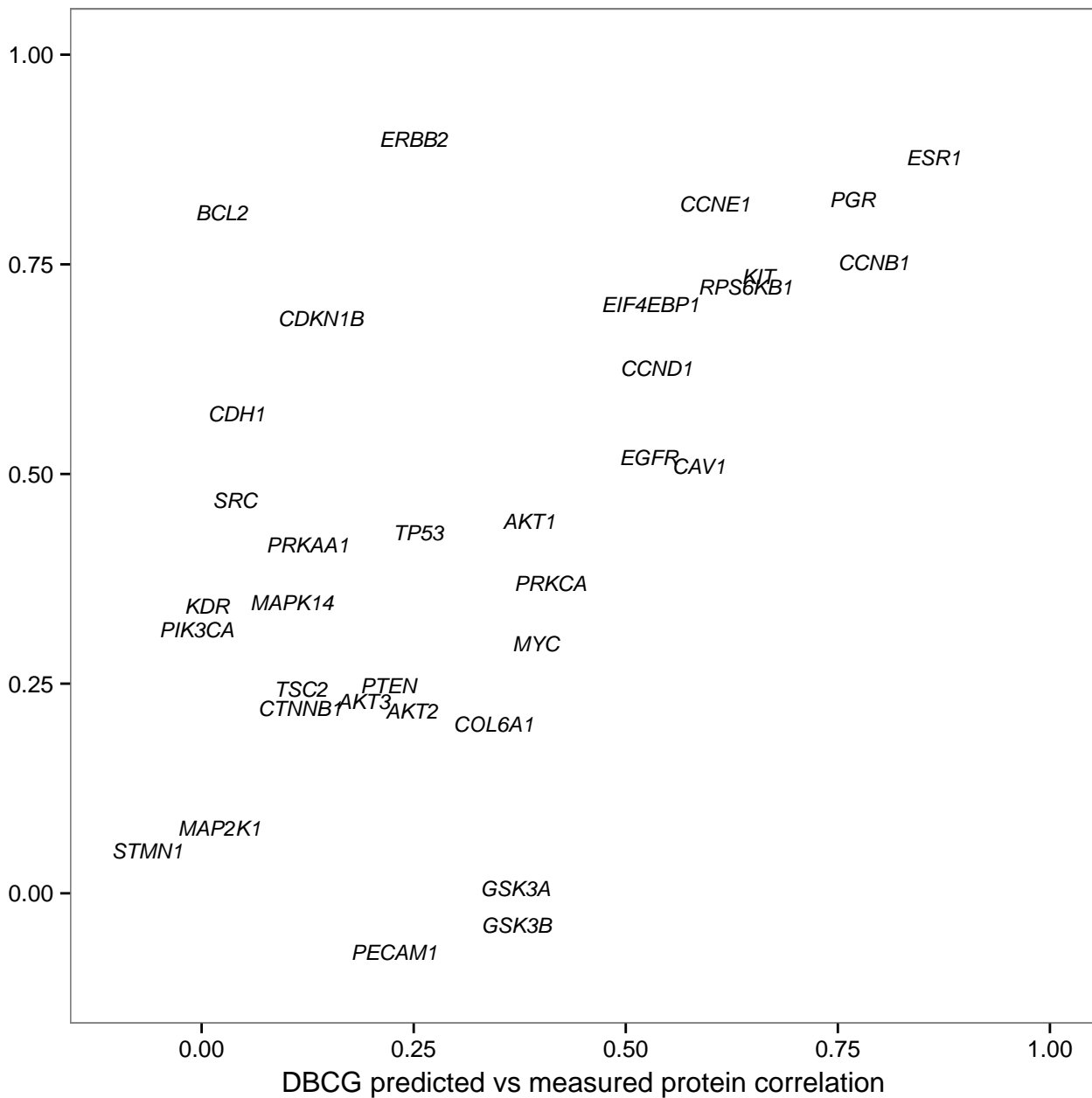

Supplement: Additional file 14: — Comparison of predicted versus measured protein in the DBCG and TCGA data sets. To calculate predicted protein expression, the estimated mRNA and miRNA coefficients from the Oslo2 cohort were fitted into equation (4) using the actual miRNA and mRNA expression data of the DBCG and TCGA data sets, respectively. Then the predicted and the measured protein expression were compared using correlation. The x- and y-axis represent Pearson correlation. [file 13073_2015_135_MOESM14_ESM.pdf]
