# Supplementary material for: Integrated analysis reveals microRNA networks coordinately expressed with key proteins in breast cancer
Source: Genome Med. 2015 Feb 2;7(1):21. doi: 10.1186/s13073-015-0135-5 (PMC4396592; doi:10.1186/s13073-015-0135-5)

Assessing the effect of miRNAs on protein expression

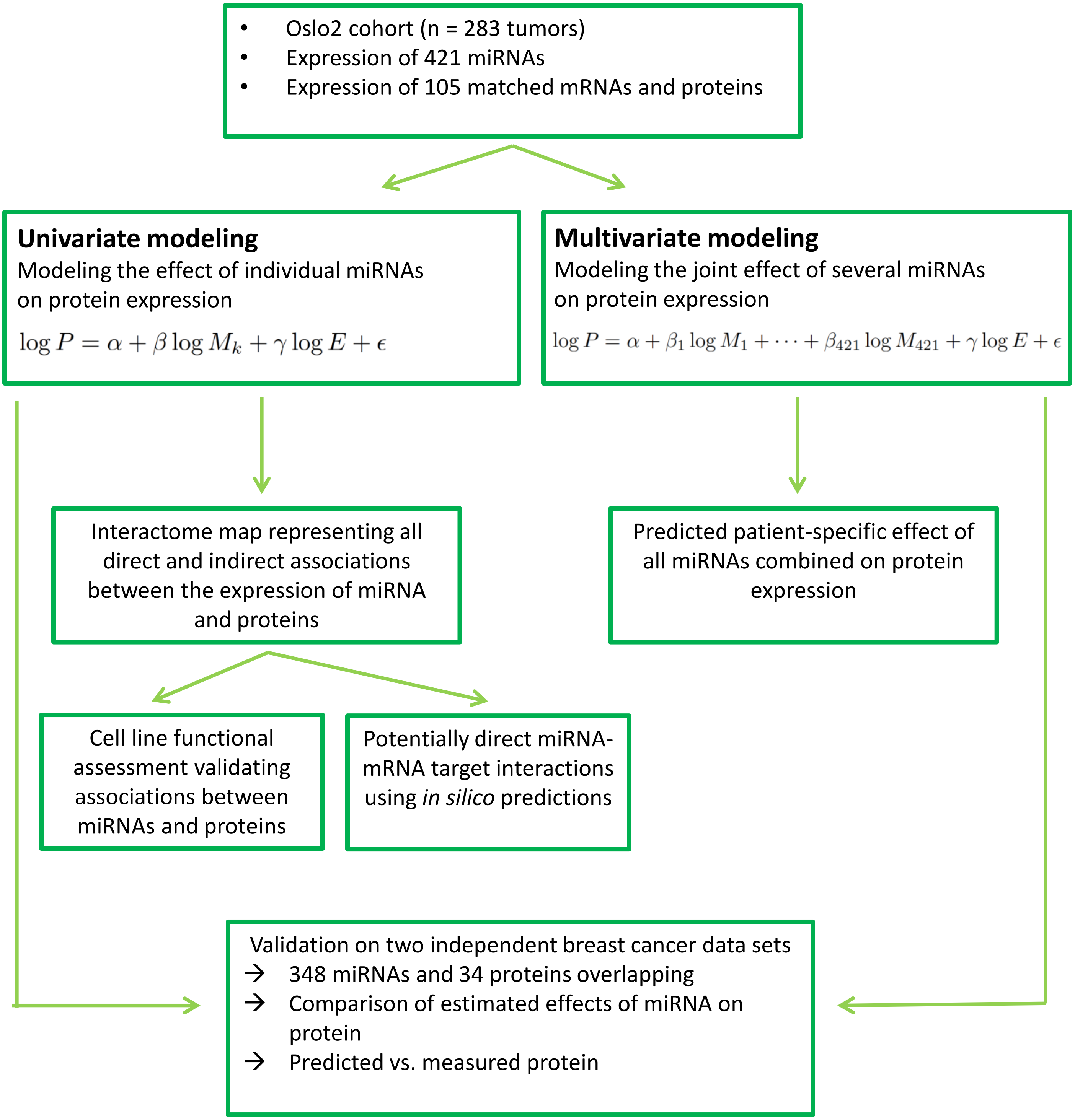

Supplement: Additional file 2: — A schematic outline of the approach used to assess the effect of miRNAs on protein expression. [file 13073_2015_135_MOESM2_ESM.pdf]
