# Supplementary material for: Integrated analysis reveals microRNA networks coordinately expressed with key proteins in breast cancer
Source: Genome Med. 2015 Feb 2;7(1):21. doi: 10.1186/s13073-015-0135-5 (PMC4396592; doi:10.1186/s13073-015-0135-5)
Supplement: Additional file 3: — Supplementary Methods. [file 13073_2015_135_MOESM3_ESM.pdf]

Supplementary Methods

miRNA expression profiling

Total RNA was isolated with TRIzol (Invitrogen, Carlsbad, CA, USA), and 100 ng RNA was used as input for labeling and hybridization to the array. Expression of miRNA was measured by the one-color microarray Human miRNA Microarray Kit (V2) design ID 029297 (Agilent Technologies, Santa Clara, CA, USA) according to the protocol supplied by the manufacturer (miRNA Microarray System v2.3). The miRNA array is based on miRBase release 14.0 and contains 887 human miRNAs. Furthermore, 14,907 features (60-mers) including 715 control probes are spotted on one array; hence each miRNA is on average replicated ~16 times. Scanning was performed on Agilent Scanner G2565A. Samples were processed using Feature Extraction (FE) version 10.7.3.1 (Agilent Technologies). The data were log_2-transformed and centered on the 90th percentile using GeneSpring GX v.11.0. (Agilent Technologies). Quality was assessed by the quality control parameters in FE. miRNAs detected in less than 10% of samples were removed, resulting in 421 miRNAs considered to be expressed in the Oslo2 cohort. The miRNA expression data have been submitted to the Gene Expression Omnibus (GEO) database with accession number GSE58210.

Gene expression profiling

Expression of mRNA was measured using SurePrint G3 Human GE 8x60K one-color microarrays (Agilent Technologies) according to the manufacturer's protocol (One-Color Microarray-Based Gene Expression Analysis, Low Input Quick Amp Labeling, v.6.5, May 2010). For each sample, 100 ng of RNA was amplified and hybridized on the array, which includes 42,405 unique 60-mer probes, targeting 27,958 Entrez genes and 7,419 lincRNAs. Scanning was performed with Agilent Scanner G2565A, using AgilentG3_GX_1Color as profile. Signals were extracted using FE v.10.7.3.1 and protocol GE1_107_Sep09 (Agilent Technologies). Arrays were log_2-transformed, quantile normalized and hospital-adjusted by subtracting from each probe value the mean probe value among samples from the same hospital. Finally, values corresponding to probes with identical Entrez ID were averaged to form a single expression value per gene. Molecular subtype classification of each tumor sample based on centroid correlation was done using the PAM50 classification (1). The mRNA expression data have been submitted to the GEO database with accession number GSE58212.

Protein expression profiling

Protein levels were determined using Reverse Phase Protein Array (RPPA), a platform where single protein levels can be measured across a series of samples simultaneously (2). Frozen tumor samples were lysed by homogenization in lysis buffer containing proteinase inhibitors and phosphatase inhibitors. The tumor lysates were diluted to 1.33 mg/ml concentration as assessed by bicinchonic acid assay (BCA) and boiled in 1% SDS and 2-mercaptoethanol. Supernatants were manually diluted in five serial 2-fold dilutions with lysis buffer. The samples were spotted onto and immobilized on nitrocellulose-coated FAST slides. The slides were probed with 105 primary highly validated antibodies in the appropriate dilution. The signal intensity was captured by a biotin conjugated secondary antibody and was amplified by DakoCytomation-catalyzed system (Dako, Glostrup, Denmark). Slides were scanned, analyzed and quantitated using MicroVigene software (VigeneTech Inc., Carlise, MA, USA) to generate spot signal intensities. These were then processed by the R package SuperCurve (version 1.01), available at http://bioinformatics.mdanderson.org/OOMPA (3). The protein concentrations were derived from the supercurve for each sample by curve fitting, $\log_2$-transformed, and the relative concentrations were normalized by median centering of the samples for each of the antibodies. RPPA subtypes were obtained using non-negative matrix factorization as done in (4). The protein expression data can be found in Supplementary File 2Q.

miRNA target predictions

Three *in silico* miRNA target prediction algorithms were used to identify putative miRNA binding sites in the transcripts coding for the 86 (out of 105 studied) proteins for which significant and negative associations to miRNAs were predicted by our model; TargetScan (5), miRanda (6) and PicTar (7).

TargetScan predictions. The "Conserved_Site_Context_Scores.txt" file was downloaded from

http://www.targetscan.org/cgi-bin/targetscan/data_download.cgi?db=vert_61

(based on TargetScan release 6.2 in June 2012). This file contains all TargetScan predictions for conserved binding sites in mRNAs, for both conserved and non-conserved miRNAs. From this file, all human predictions were considered (species id 9606). Matching the genes on gene symbol, 81 of the 86 genes were found to have predicted and conserved miRNA sites.

miRanda predictions. The August 2010 release was downloaded from http://www.microrna.org/microrna/getDownloads.do. The file for human target site

predictions for good mirSVR score and conserved miRNAs, ”hg19_predictions_S_C_aug2010.txt”, and the file for good mirSVR score and non-conserved miRNAs, "hg19_predictions_S_0_aug2010.txt", were merged. Among the 86 unique genes, one gene was not predicted to have any miRNA binding sites.

PicTar predictions. Two files were downloaded from the web site http://hgdownload.cse.ucsc.edu/goldenPath/hg17/database/: the "picTarMiRNA4Way.txt" and the "picTarMiRNA5Way.txt" (both last modified in April 2006). The first file contains miRNA-target predictions based on target site conservation in four species; human, mouse, rat and dog. The second file is based on predictions from five species; human, mouse, rat, dog and chicken. The NM-accession numbers in the PicTar files were converted into gene symbols using IPA (Ingenuity Systems, www.ingenuity.com), and among the 86 unique genes with significant negative associations, 56 had miRNA binding sites predicted by PicTar.

References

1. Parker JS, Mullins M, Cheang MCU, Leung S, Voduc D, Vickery T, et al. Supervised Risk Predictor of Breast Cancer Based on Intrinsic Subtypes. Journal of Clinical Oncology 2009;27:1160-7.
2. Tibes R, Qiu Y, Lu Y, Hennessy B, Andreeff M, Mills GB, et al. Reverse phase protein array: validation of a novel proteomic technology and utility for analysis of primary leukemia specimens and hematopoietic stem cells. Molecular Cancer Therapeutics 2006;5:2512-21.
3. Hu J, He X, Baggerly KA, Coombes KR, Hennessy BTJ, Mills GB. Non-parametric quantification of protein lysate arrays. Bioinformatics 2007;23:1986-94.
4. The Cancer Genome Atlas Network. Comprehensive molecular portraits of human breast tumours. Nature 2012;490:61-70.
5. Friedman RC, Farh KK-H, Burge CB, Bartel DP. Most mammalian mRNAs are conserved targets of microRNAs. Genome Research 2009;19:92-105.
6. John B, Enright AJ, Aravin A, Tuschl T, Sander C, Marks DS. Human microRNA targets. Plos Biology 2004;2:e363.
7. Krek A, Grun D, Poy MN, Wolf R, Rosenberg L, Epstein EJ, et al. Combinatorial microRNA target predictions. Nat Genet 2005;37:495-500.
