# Supplementary material for: Integrated analysis reveals microRNA networks coordinately expressed with key proteins in breast cancer
Source: Genome Med. 2015 Feb 2;7(1):21. doi: 10.1186/s13073-015-0135-5 (PMC4396592; doi:10.1186/s13073-015-0135-5)
Supplement: Additional file 6: — Scatterplots of mRNA and protein expression for all 105 proteins. Each dot represents a patient and the colors indicate PAM50 molecular subtype (red: basal-like; pink: HER2-enriched; green: normal-like; dark blue: luminal A; light blue: luminal B). [file 13073_2015_135_MOESM6_ESM.pdf]

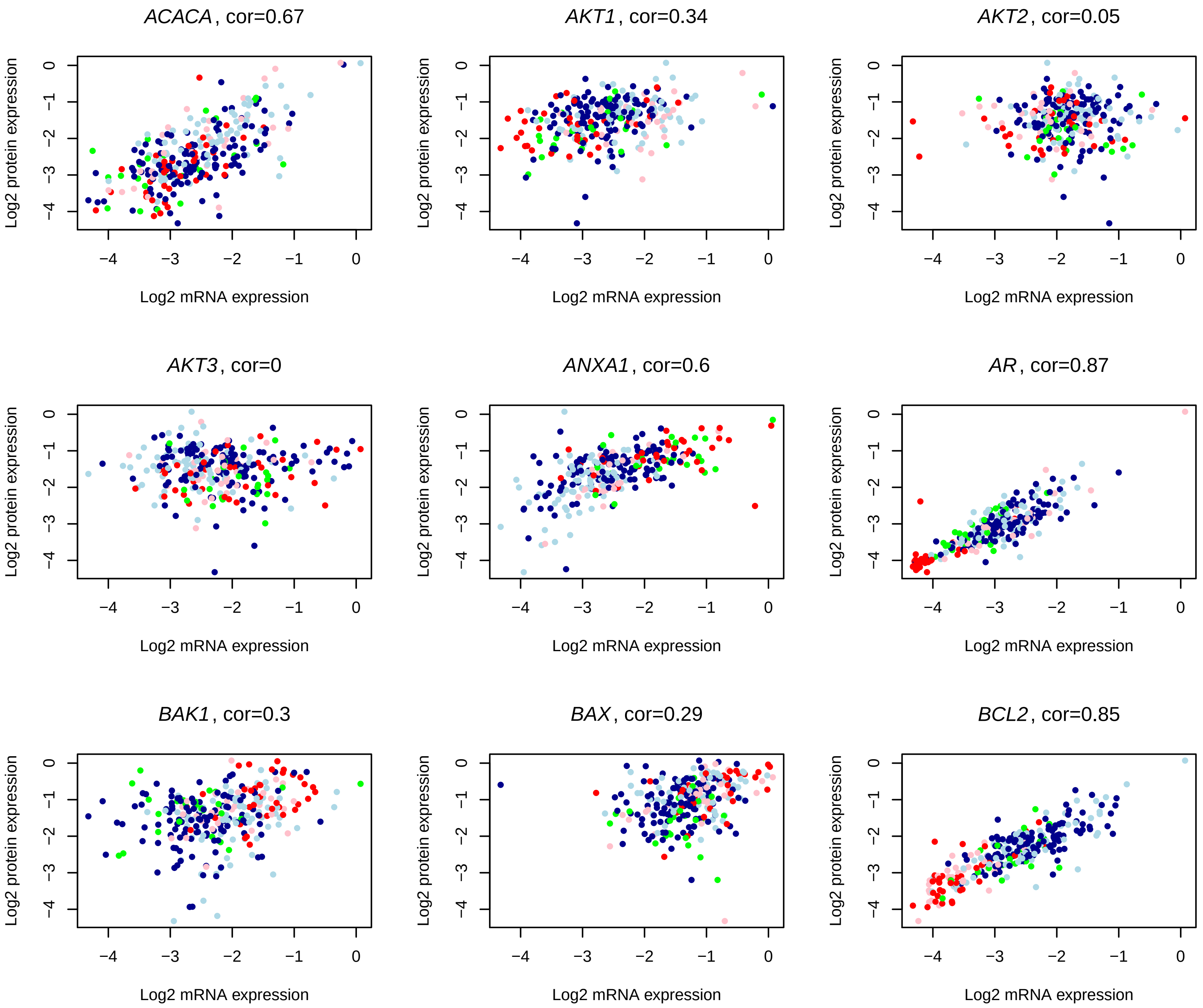
ACACA, cor=0.67
AKT1, cor=0.34
AKT2, cor=0.05
AKT3, cor=0
ANXA1, cor=0.6
AR, cor=0.87
BAK1, cor=0.3
BAX, cor=0.29
BCL2, cor=0.85
Log2 protein expression
Log2 mRNA expression
0
−1
−2
−3
−4

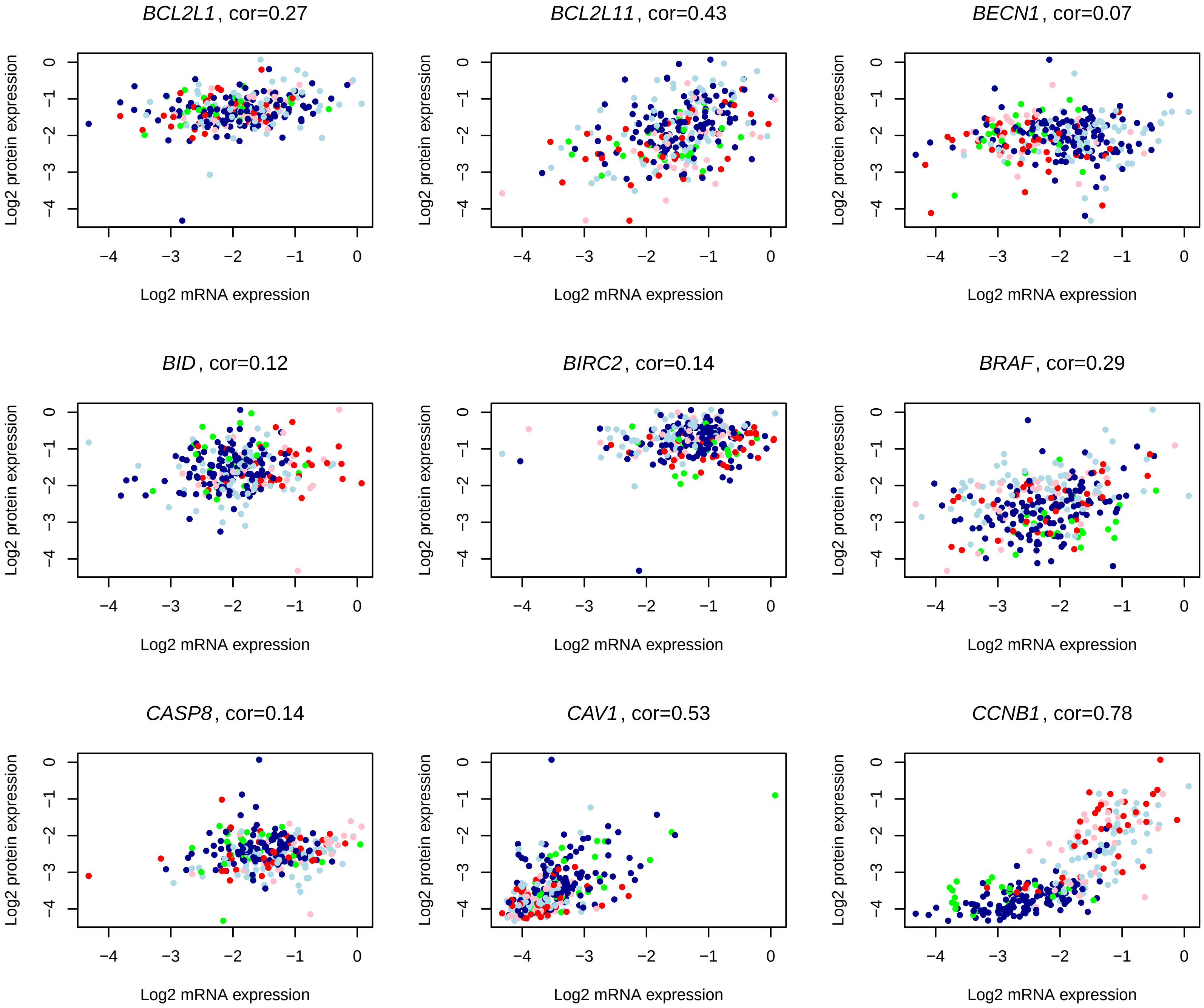

BCL2L1, cor=0.27
BCL2L11, cor=0.43
BECN1, cor=0.07
BID, cor=0.12
BIRC2, cor=0.14
BRAF, cor=0.29
CASP8, cor=0.14
CAV1, cor=0.53
CCNB1, cor=0.78
Log2 protein expression
Log2 mRNA expression

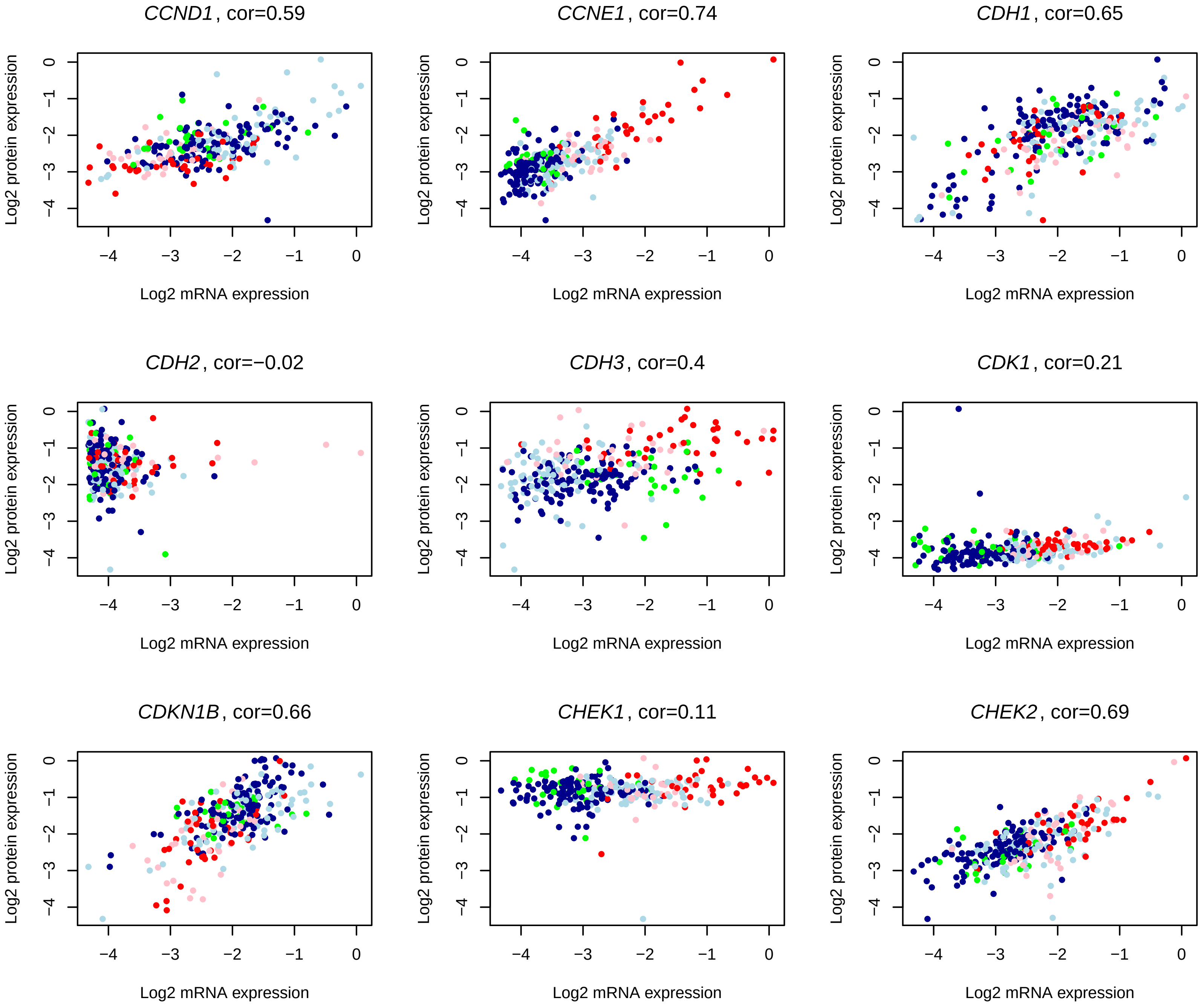

CCND1, cor=0.59
CCNE1, cor=0.74
CDH1, cor=0.65
CDH2, cor=−0.02
CDH3, cor=0.4
CDK1, cor=0.21
CDKN1B, cor=0.66
CHEK1, cor=0.11
CHEK2, cor=0.69
Log2 protein expression
Log2 mRNA expression
−4
−3
−2
−1
0

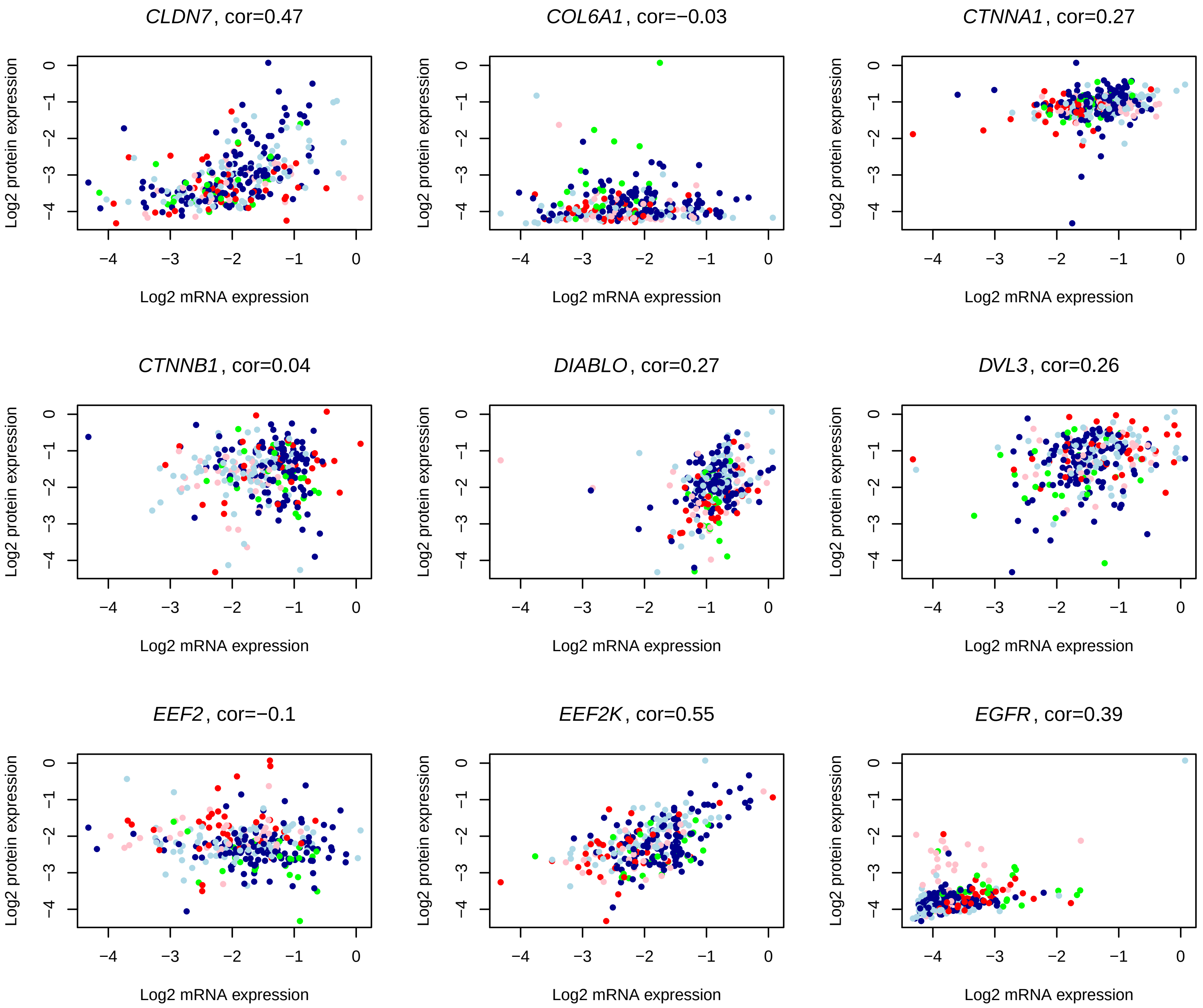
CLDN7, cor=0.47
COL6A1, cor=−0.03
CTNNA1, cor=0.27
CTNNB1, cor=0.04
DIABLO, cor=0.27
DVL3, cor=0.26
EEF2, cor=−0.1
EEF2K, cor=0.55
EGFR, cor=0.39
Log2 protein expression
Log2 mRNA expression
−4
−3
−2
−1
0

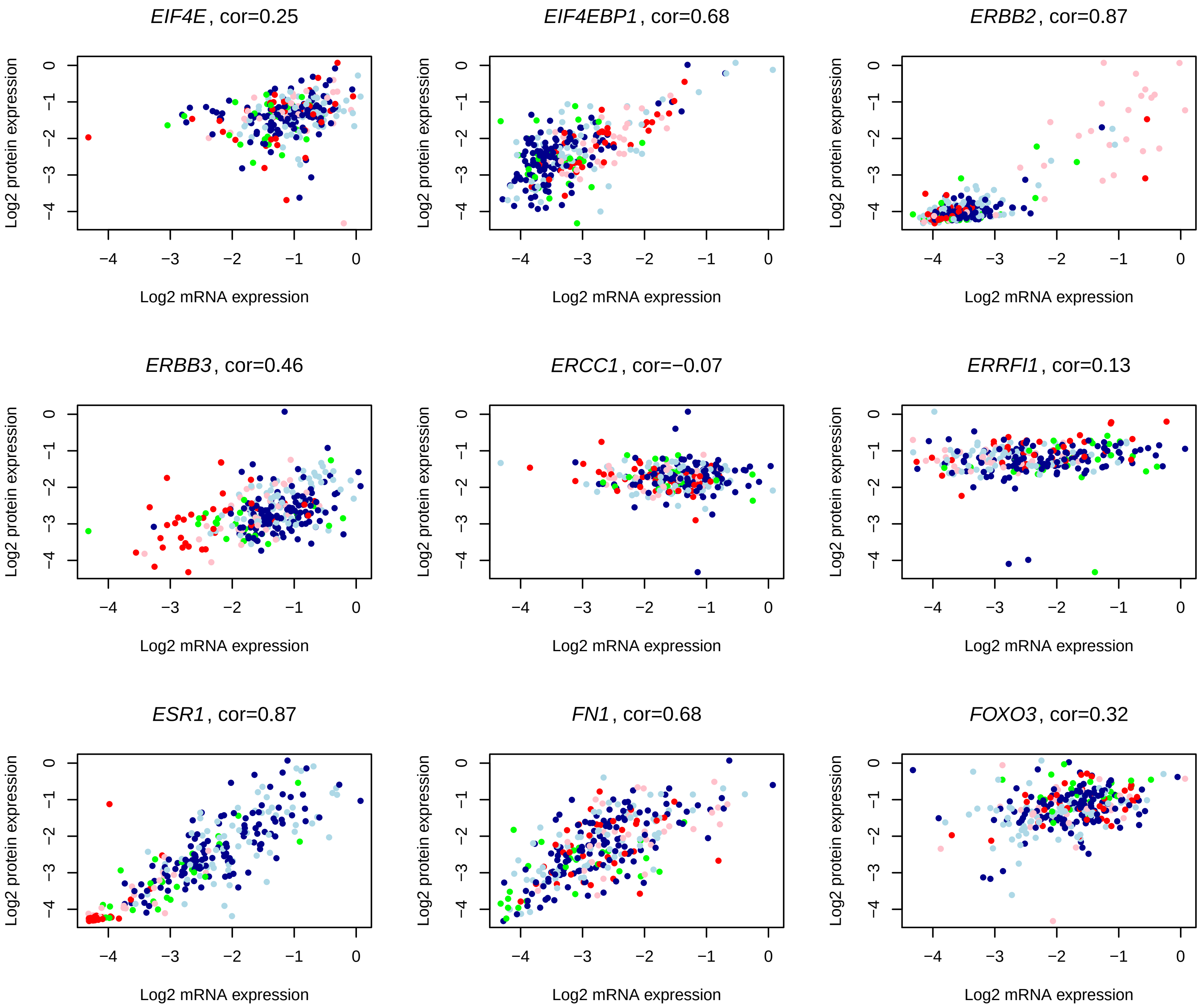
EIF4E, cor=0.25
EIF4EBP1, cor=0.68
ERBB2, cor=0.87
ERBB3, cor=0.46
ERCC1, cor=−0.07
ERRFI1, cor=0.13
ESR1, cor=0.87
FN1, cor=0.68
FOXO3, cor=0.32
Log2 protein expression
Log2 mRNA expression

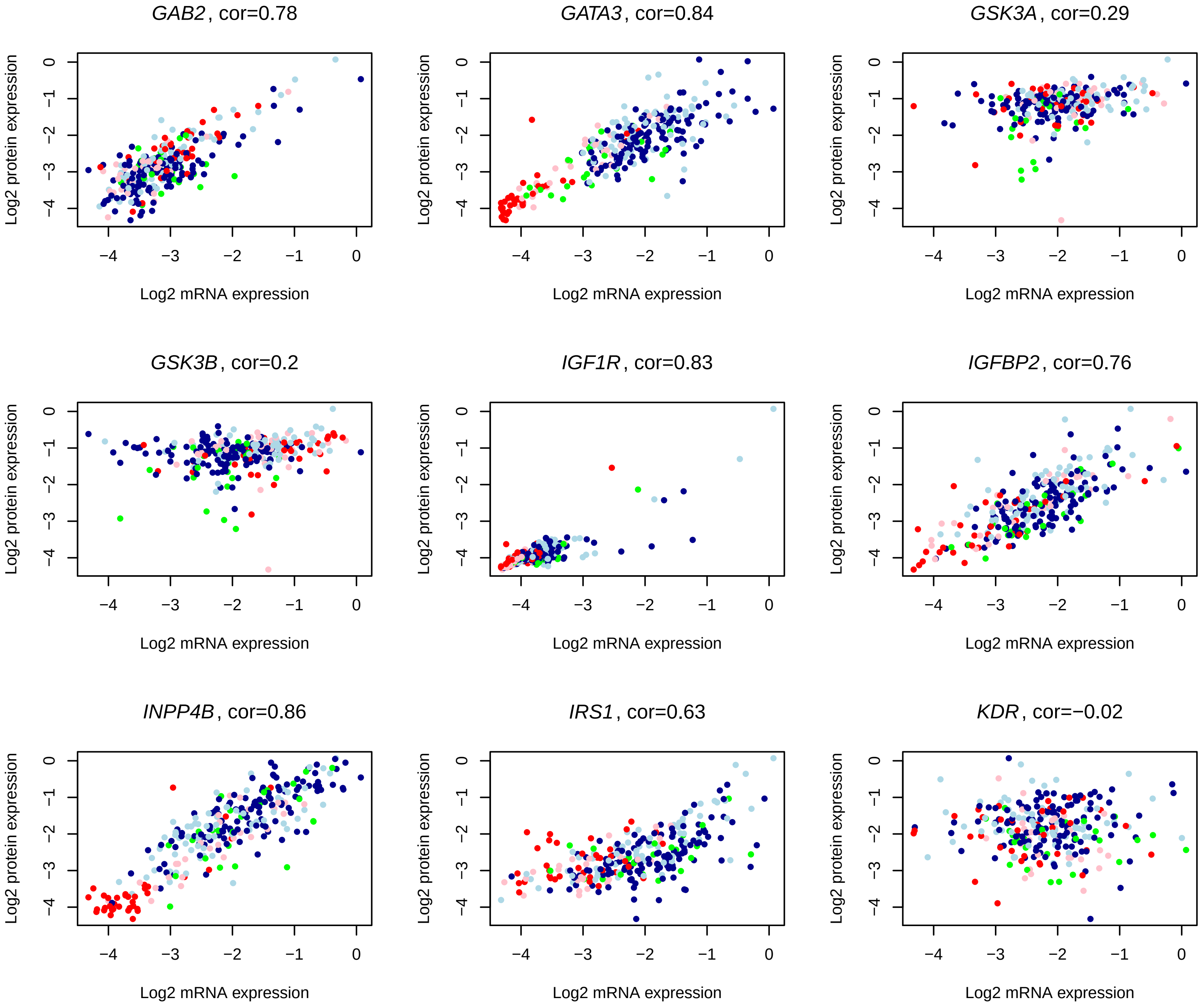
GAB2, cor=0.78
GATA3, cor=0.84
GSK3A, cor=0.29
GSK3B, cor=0.2
IGF1R, cor=0.83
IGFBP2, cor=0.76
INPP4B, cor=0.86
IRS1, cor=0.63
KDR, cor=−0.02
Log2 protein expression
Log2 mRNA expression
−4
−3
−2
−1
0

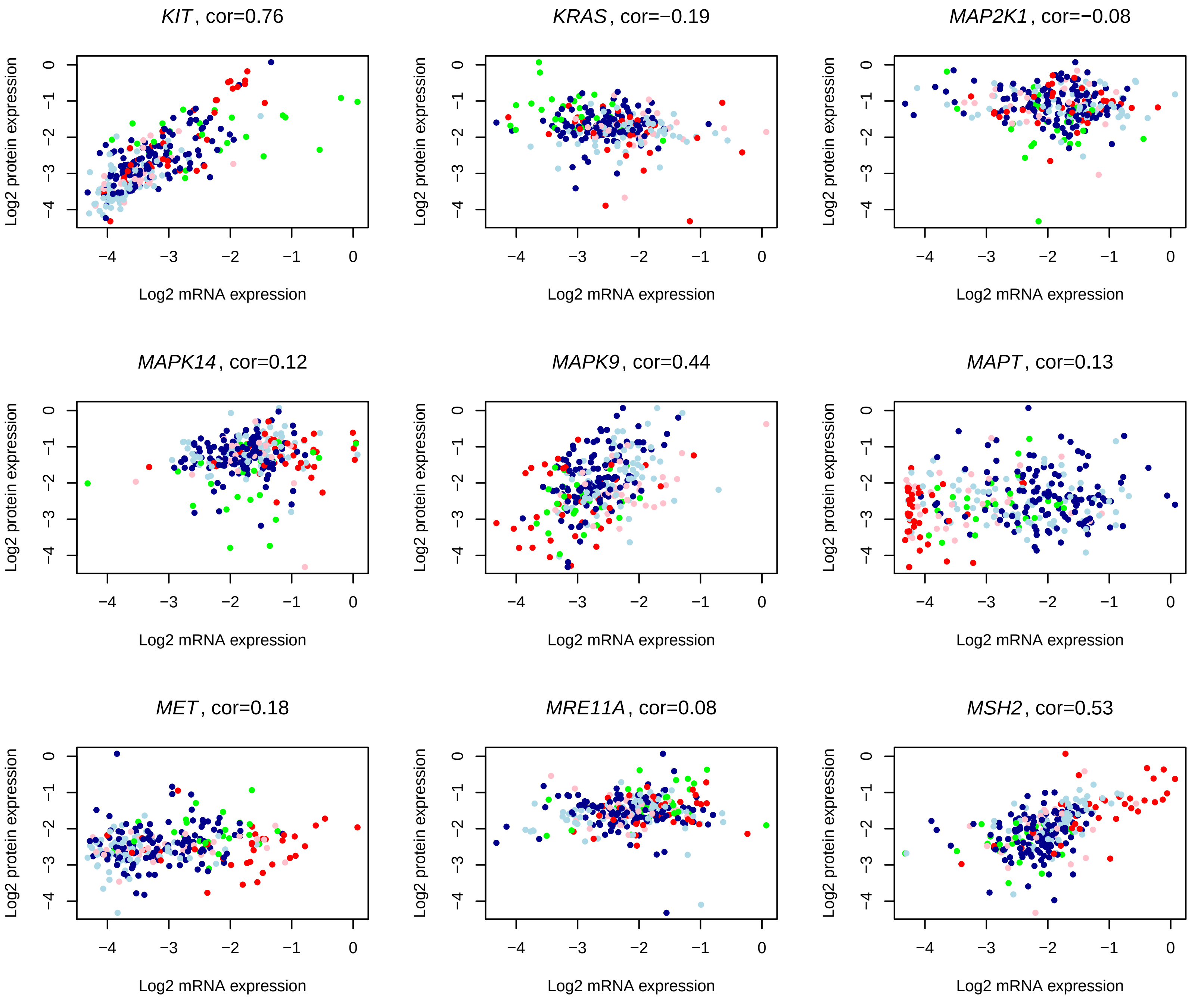

KIT, cor=0.76
KRAS, cor=−0.19
MAP2K1, cor=−0.08
MAPK14, cor=0.12
MAPK9, cor=0.44
MAPT, cor=0.13
MET, cor=0.18
MRE11A, cor=0.08
MSH2, cor=0.53
Log2 protein expression
Log2 mRNA expression

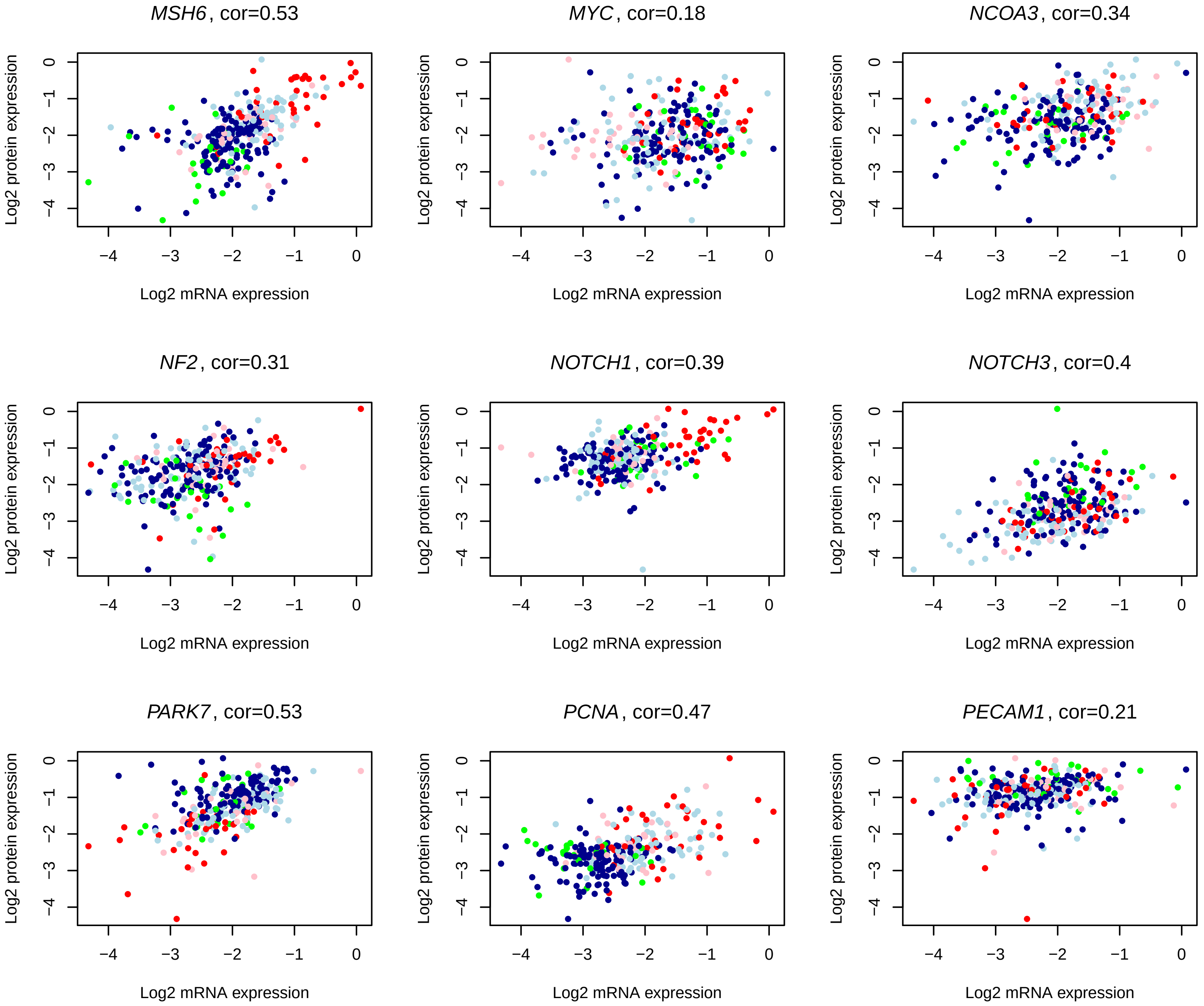

MSH6, cor=0.53
MYC, cor=0.18
NCOA3, cor=0.34
NF2, cor=0.31
NOTCH1, cor=0.39
NOTCH3, cor=0.4
PARK7, cor=0.53
PCNA, cor=0.47
PECAM1, cor=0.21
Log2 protein expression
Log2 mRNA expression
−4
−3
−2
−1
0

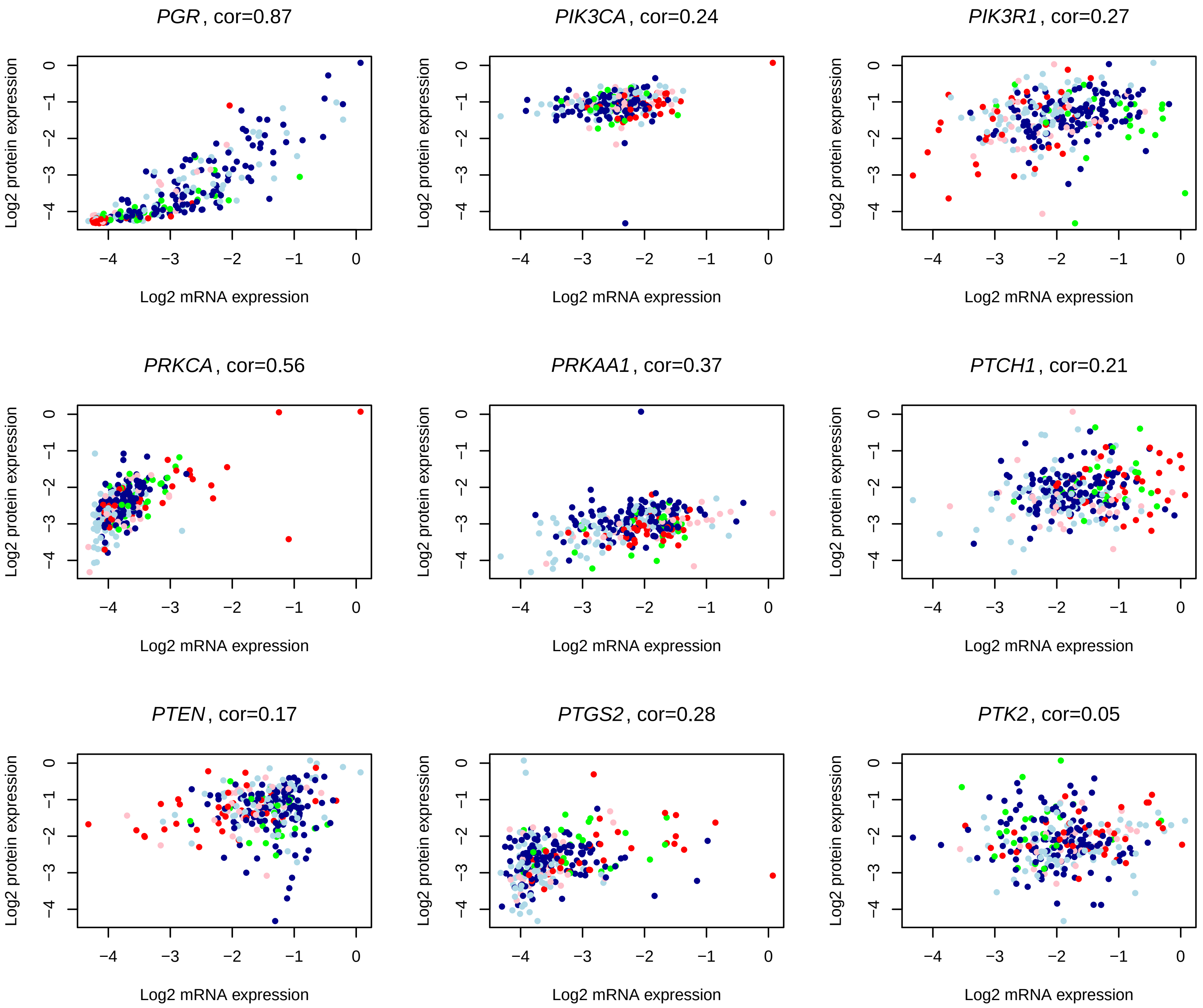
PGR, cor=0.87
PIK3CA, cor=0.24
PIK3R1, cor=0.27
PRKCA, cor=0.56
PRKAA1, cor=0.37
PTCH1, cor=0.21
PTEN, cor=0.17
PTGS2, cor=0.28
PTK2, cor=0.05
Log2 protein expression
Log2 mRNA expression
−4
−3
−2
−1
0

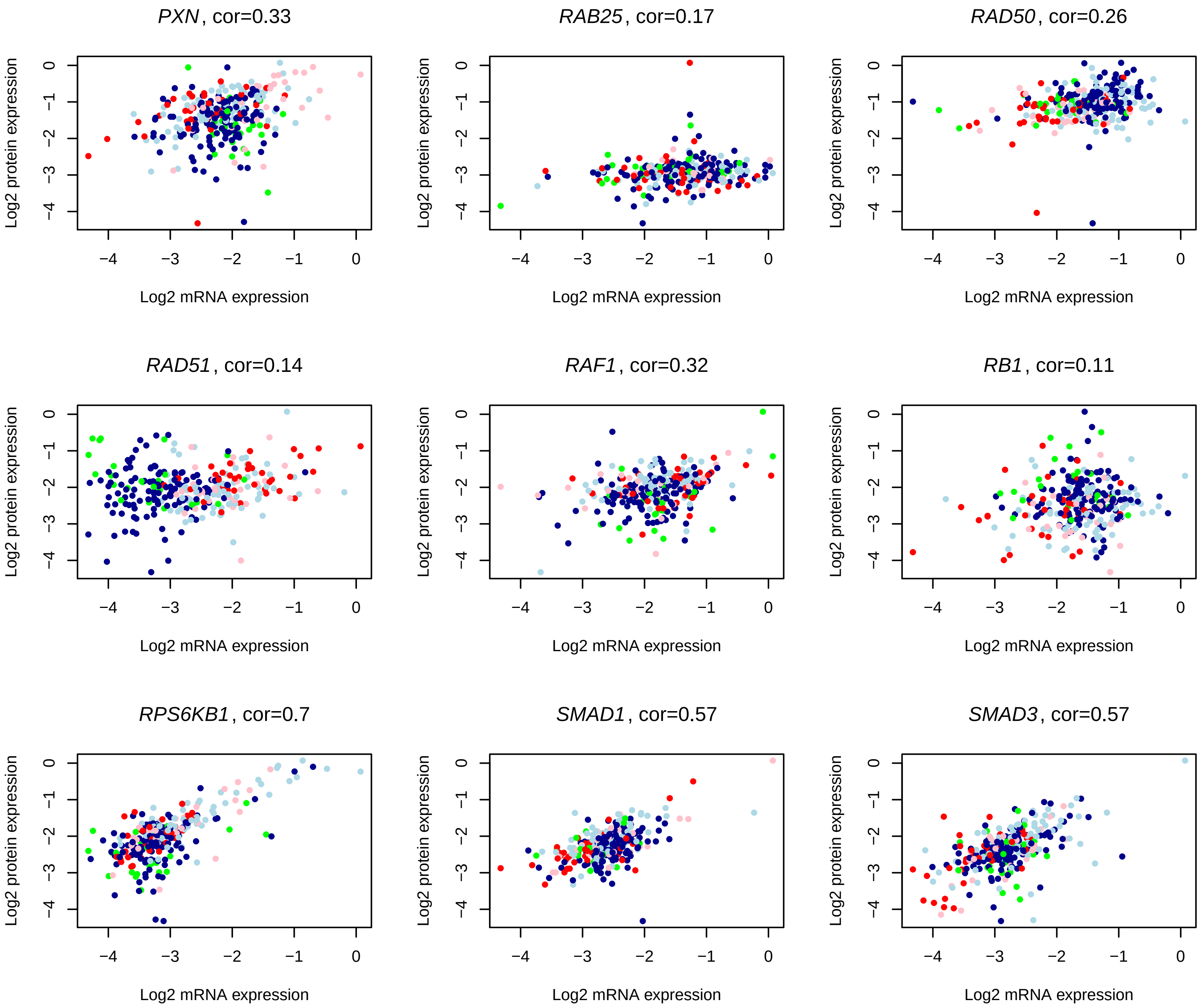
PXN, cor=0.33
RAB25, cor=0.17
RAD50, cor=0.26
RAD51, cor=0.14
RAF1, cor=0.32
RB1, cor=0.11
RPS6KB1, cor=0.7
SMAD1, cor=0.57
SMAD3, cor=0.57
Log2 protein expression
Log2 mRNA expression
0
−1
−2
−3
−4

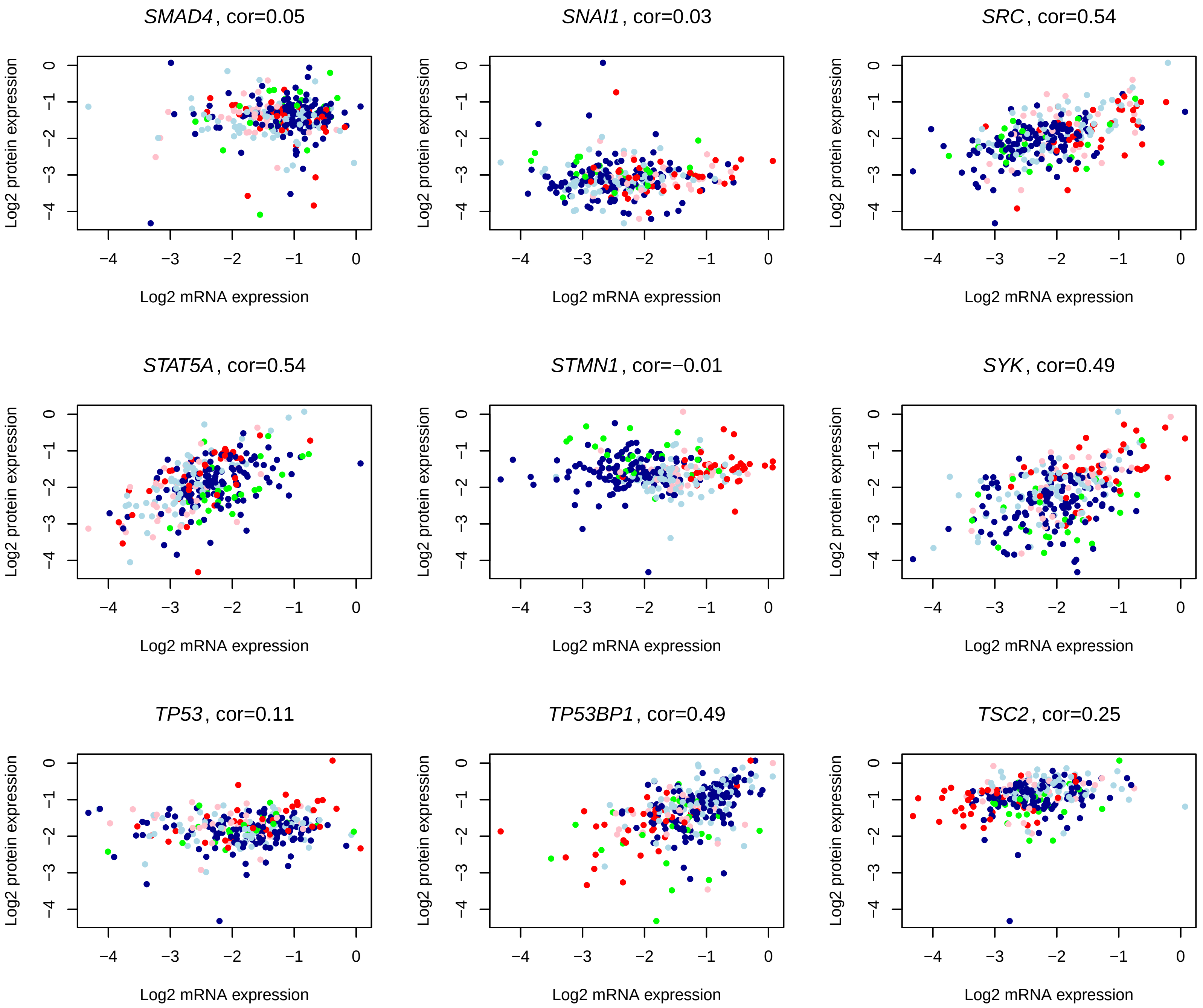
SMAD4, cor=0.05
SNAI1, cor=0.03
SRC, cor=0.54
STAT5A, cor=0.54
STMN1, cor=−0.01
SYK, cor=0.49
TP53, cor=0.11
TP53BP1, cor=0.49
TSC2, cor=0.25
Log2 protein expression
Log2 mRNA expression

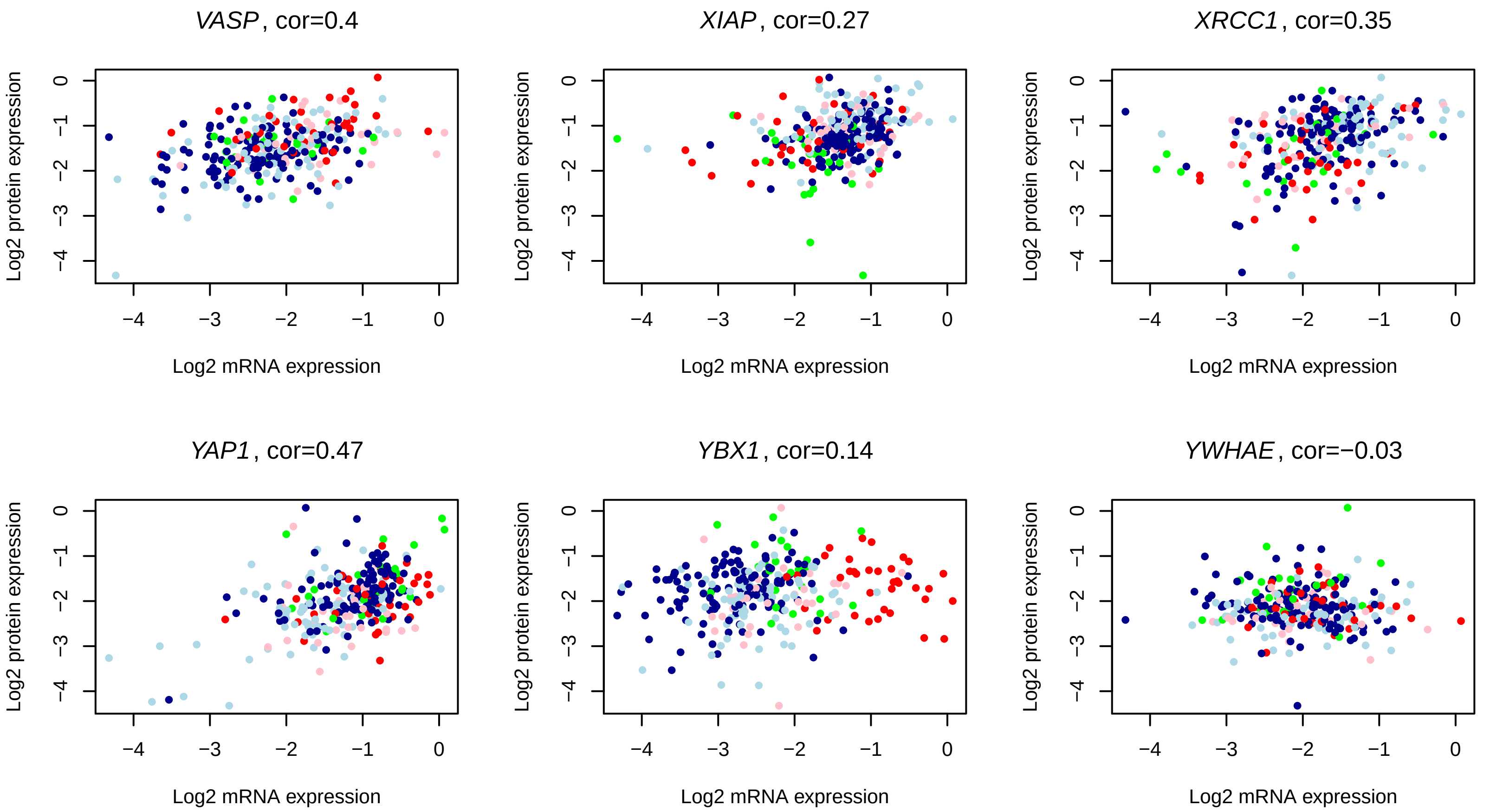

VASP, cor=0.4
XIAP, cor=0.27
XRCC1, cor=0.35
YAP1, cor=0.47
YBX1, cor=0.14
YWHAE, cor=−0.03
Log2 protein expression
Log2 mRNA expression
0
−1
−2
−3
−4
