# Supplementary material for: Integrated analysis reveals microRNA networks coordinately expressed with key proteins in breast cancer
Source: Genome Med. 2015 Feb 2;7(1):21. doi: 10.1186/s13073-015-0135-5 (PMC4396592; doi:10.1186/s13073-015-0135-5)
Supplement: Additional file 8: — Potential direct miRNA-mRNA target interactions identified from the univariate model. The depicted interactions represent significant, negative associations identified from the univariate model that were also in silico predicted. Yellow nodes represent miRNAs and blue nodes represent proteins. Three miRNA target algorithms were used to assess potential direct interactions; TargetScan [24], miRanda [25] and PicTar [26]. The full edges indicate previously validated interactions (see Additional file 4 M), and dashed edges indicate potential direct interactions. Black edges represent interactions predicted by at least two out of three algorithms, and purple edges represent interactions predicted by all three algorithms. The thickness of the edges represents relative beta values. The figure was made using Cytoscape version 2.8.3 [28]. [file 13073_2015_135_MOESM8_ESM.pdf]

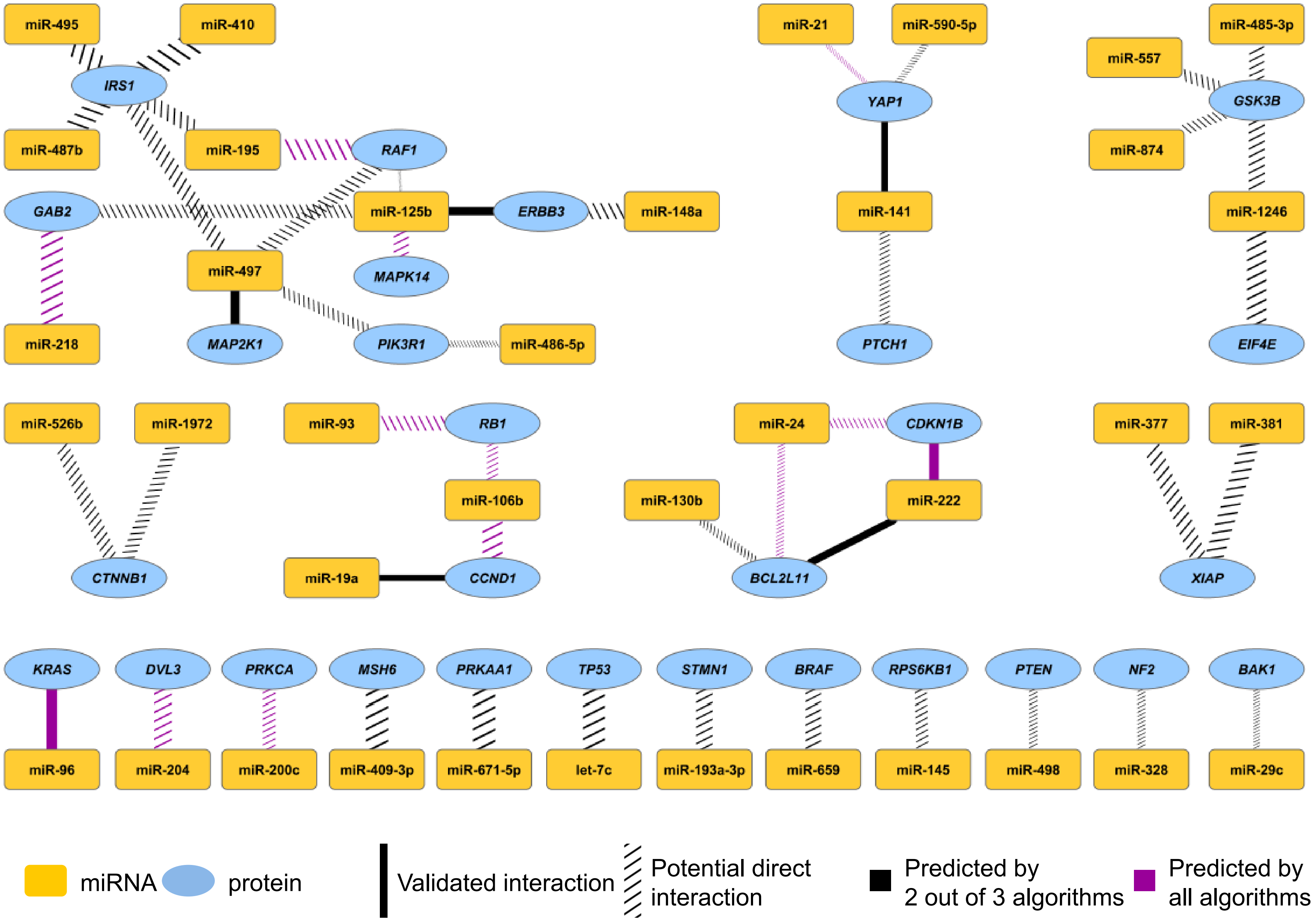
miR-495
miR-410
IRS1
miR-487b
miR-195
RAF1
GAB2
miR-125b
ERBB3
miR-148a
miR-497
MAPK14
miR-218
MAP2K1
PIK3R1
miR-486-5p
miR-21
miR-590-5p
YAP1
miR-141
PTCH1
miR-557
miR-485-3p
GSK3B
miR-874
miR-1246
EIF4E
miR-526b
miR-1972
CTNNB1
miR-93
RB1
miR-106b
miR-19a
CCND1
miR-24
CDKN1B
miR-130b
miR-222
BCL2L11
miR-377
miR-381
XIAP
KRAS
miR-96
DVL3
miR-204
PRKCA
miR-200c
MSH6
miR-409-3p
PRKAA1
miR-671-5p
TP53
let-7c
STMN1
miR-193a-3p
BRAF
miR-659
RPS6KB1
miR-145
PTEN
miR-498
NF2
miR-328
BAK1
miR-29c
miRNA
protein
Validated interaction
Potential direct interaction
Predicted by 2 out of 3 algorithms
Predicted by all algorithms
