# Supplementary material for: Integrated analysis reveals microRNA networks coordinately expressed with key proteins in breast cancer
Source: Genome Med. 2015 Feb 2;7(1):21. doi: 10.1186/s13073-015-0135-5 (PMC4396592; doi:10.1186/s13073-015-0135-5)
Supplement: Additional file 11: — Comparison of estimated values across three data sets. (i) Comparison of estimated beta values between the Oslo2 and DBCG cohort, and (ii) between the Oslo2 and TCGA cohort. (iii) Comparison of Z-score transformed P-values between the Oslo2 and DBCG cohort, (iv) and between the Oslo2 and TCGA cohort. Z-scores were obtained by applying the inverse cumulative normal distribution function to the P-values, and they follow a standard normal distribution under the null hypothesis of no effects of miRNA on protein expression. The grey lines indicate the first principal component curve of the data. [file 13073_2015_135_MOESM11_ESM.pdf]

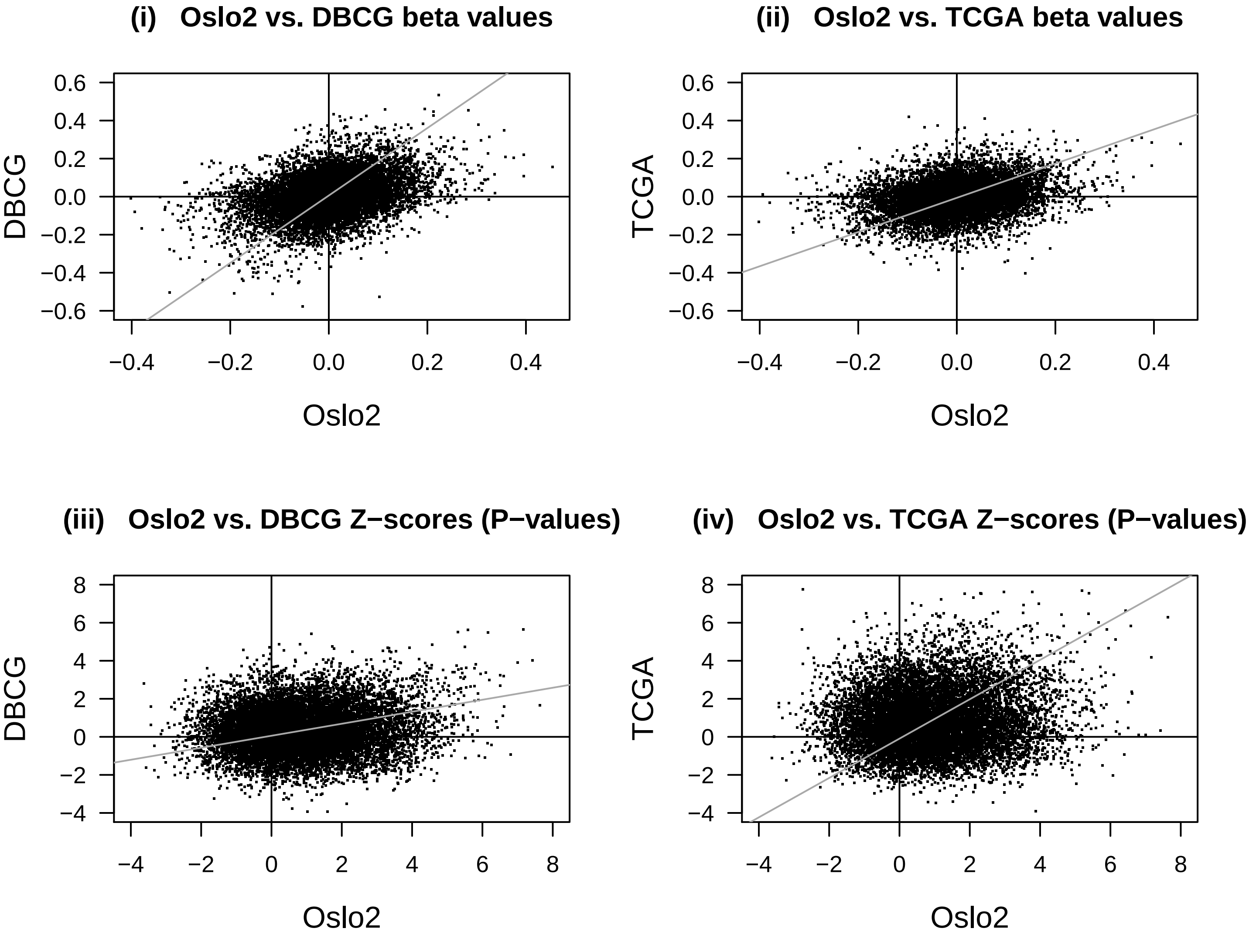

(i) Oslo2 vs. DBCG beta values
DBCG
Oslo2
(ii) Oslo2 vs. TCGA beta values
TCGA
Oslo2
(iii) Oslo2 vs. DBCG Z−scores (P−values)
DBCG
Oslo2
(iv) Oslo2 vs. TCGA Z−scores (P−values)
TCGA
Oslo2
