# Supplementary material for: Integrated analysis reveals microRNA networks coordinately expressed with key proteins in breast cancer
Source: Genome Med. 2015 Feb 2;7(1):21. doi: 10.1186/s13073-015-0135-5 (PMC4396592; doi:10.1186/s13073-015-0135-5)
Supplement: Additional file 13: — Scatterplots comparing estimated beta values from Oslo2 vs. DBCG and Oslo2 vs. TCGA. Each dot represents a miRNA and the x-axes represent estimated beta values for the Oslo2 cohort from the univariate analysis. The y-axes represent estimated beta values for the DBCG and TCGA data sets, respectively, from the univariate analysis. The dashed lines indicate the least squares fit to the data and the red line indicates the first principal component curve of the data. Pearson correlation is indicated in the corner of each plot. [file 13073_2015_135_MOESM13_ESM.pdf]

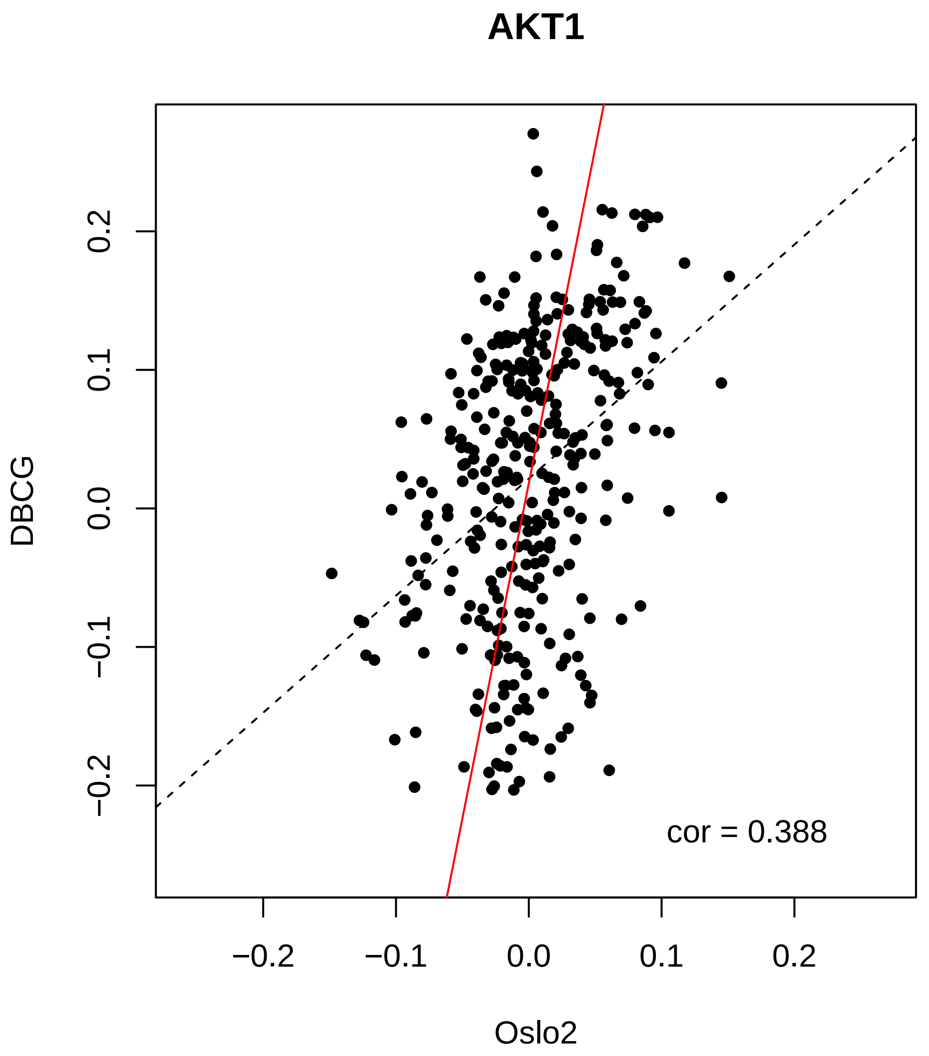

AKT1
DBCG
Oslo2
0.2
0.1
0.0
−0.1
−0.2
cor = 0.388

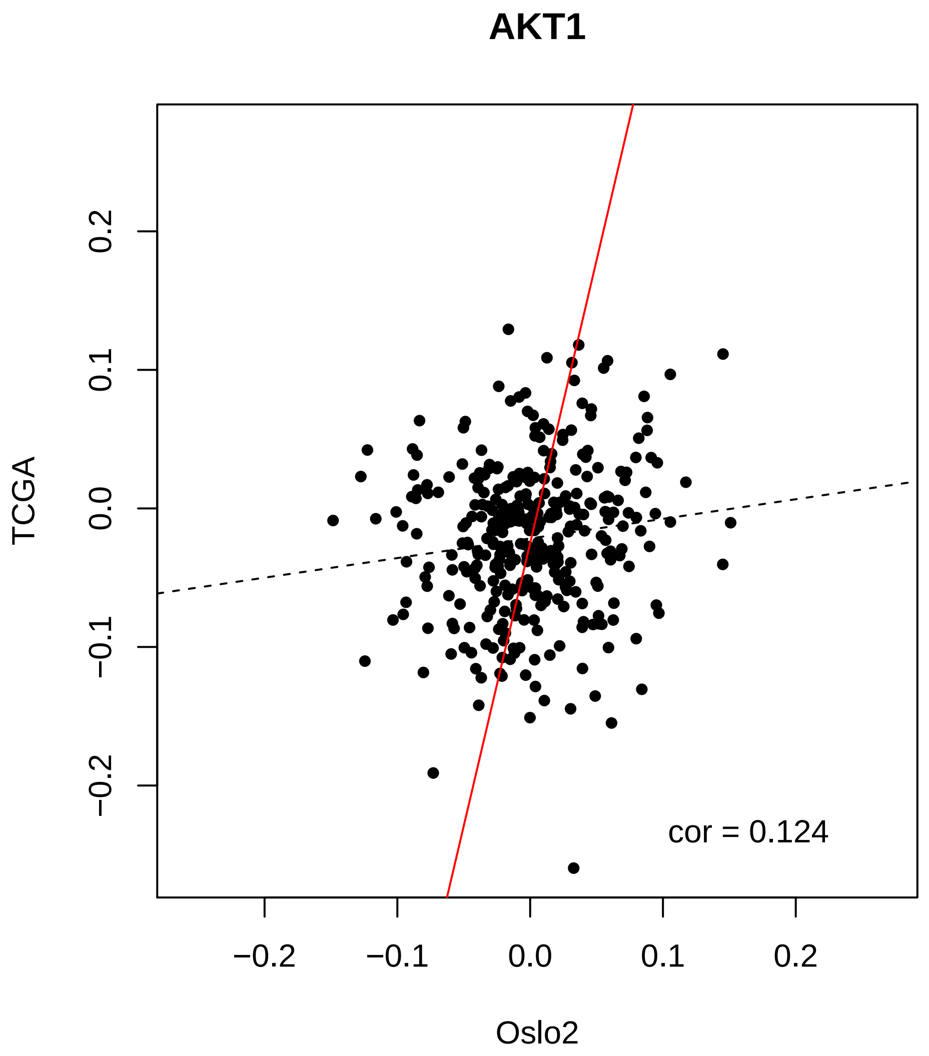

AKT1
TCGA
Oslo2
0.2
0.1
0.0
−0.1
−0.2
cor = 0.124

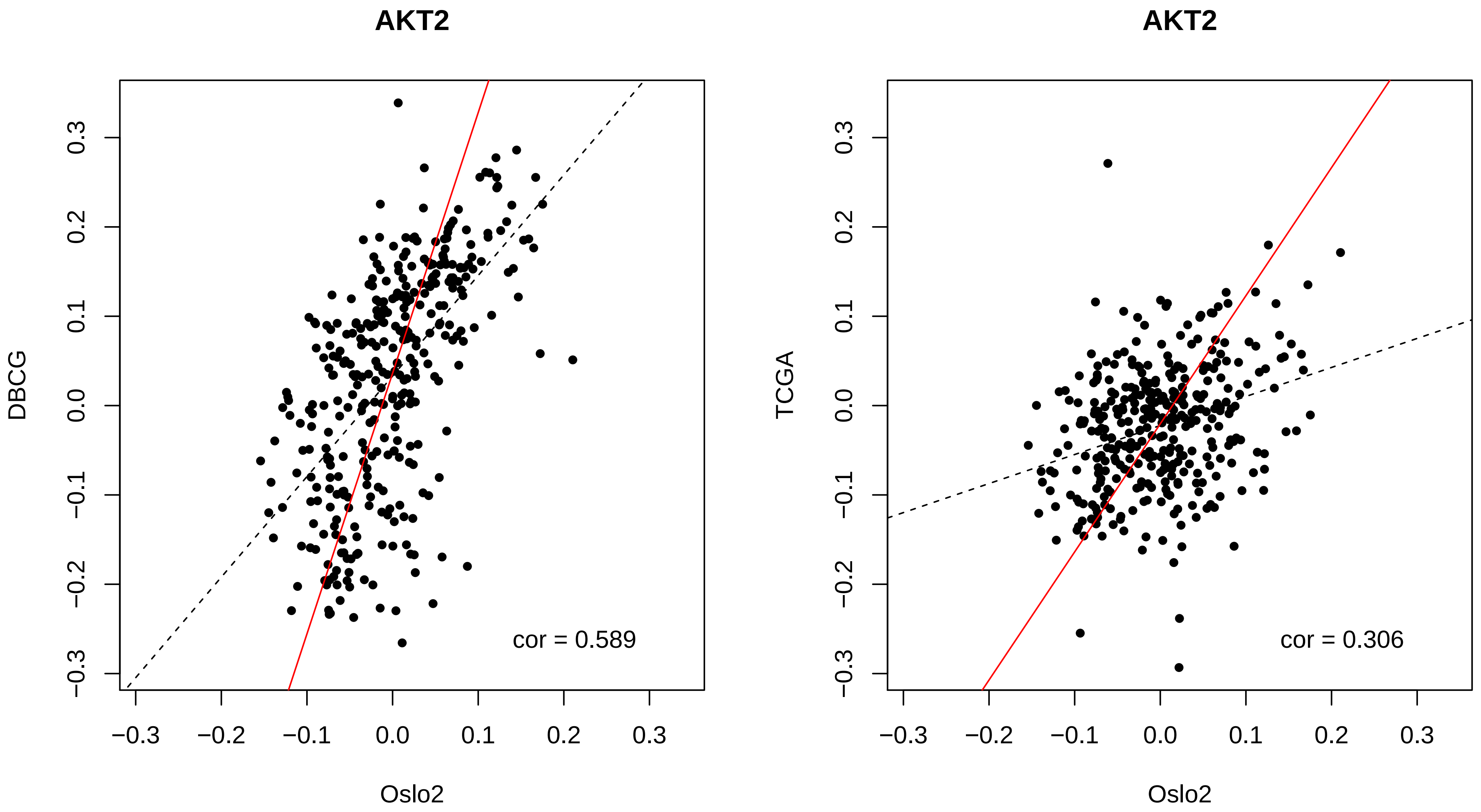
AKT2
DBCG
Oslo2
cor = 0.589
AKT2
TCGA
Oslo2
cor = 0.306
−0.3
−0.2
−0.1
0.0
0.1
0.2
0.3

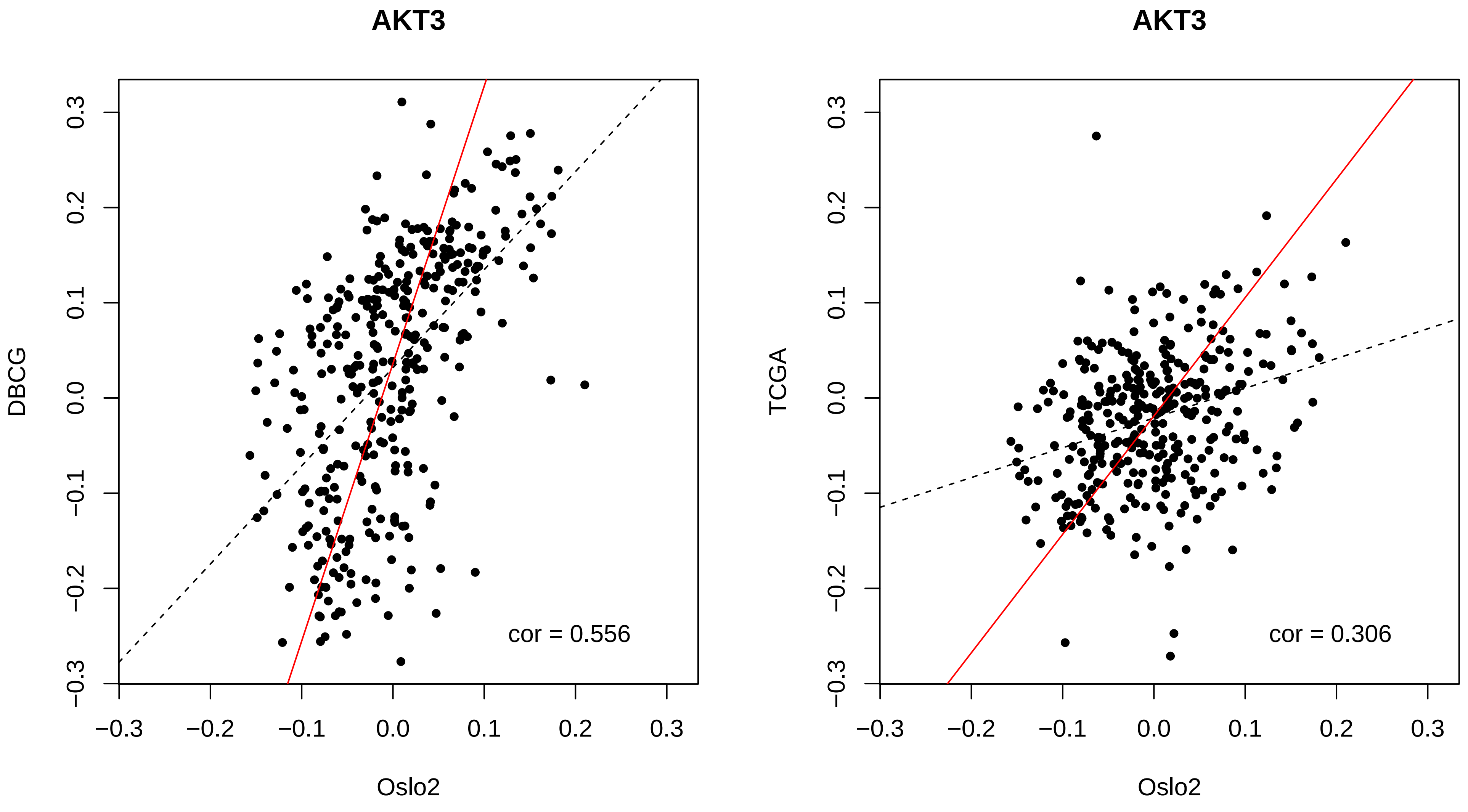

AKT3
DBCG
Oslo2
cor = 0.556
AKT3
TCGA
Oslo2
cor = 0.306

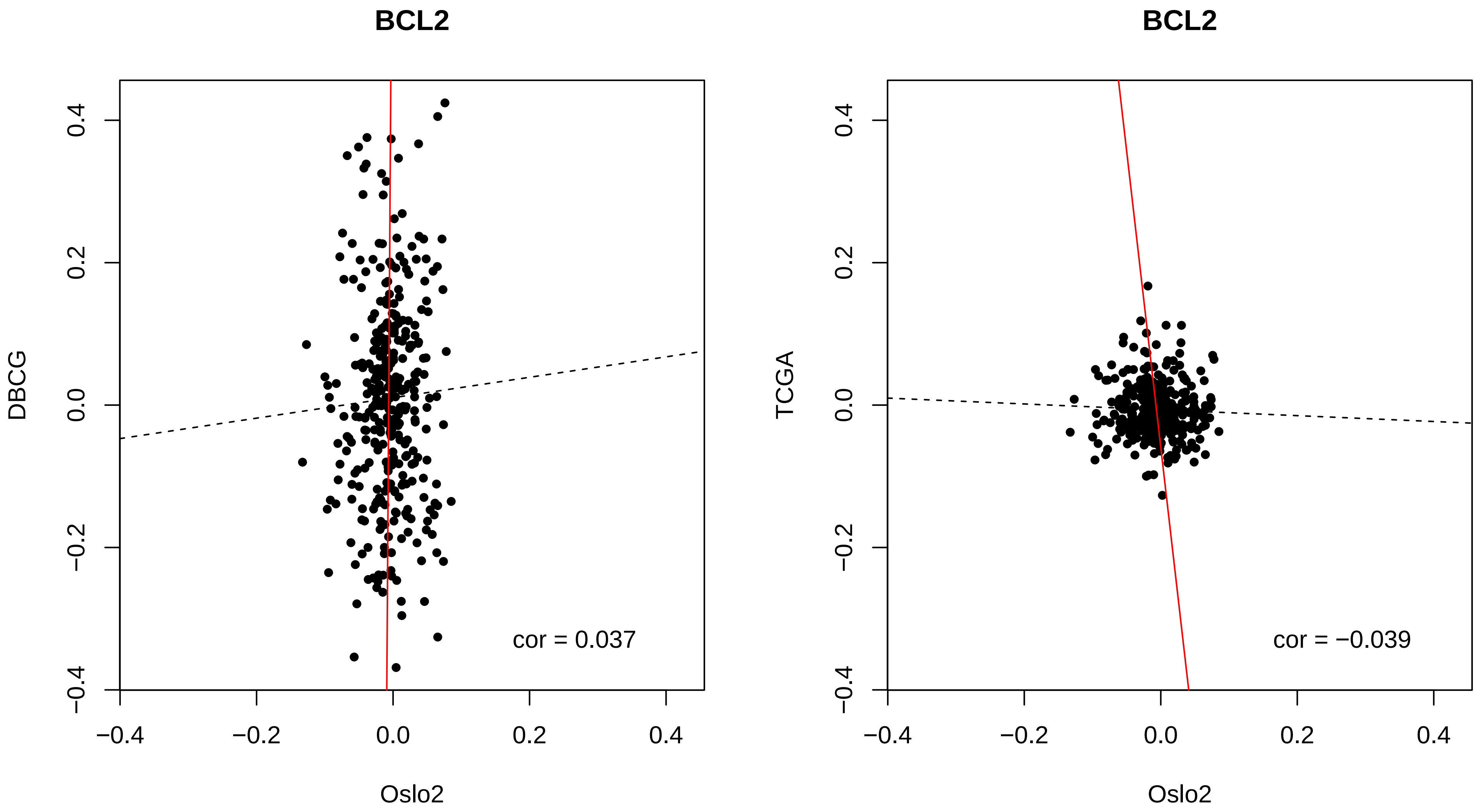
BCL2
DBCG
0.4
0.2
0.0
−0.2
−0.4
−0.4
−0.2
0.0
0.2
0.4
Oslo2
cor = 0.037
BCL2
TCGA
0.4
0.2
0.0
−0.2
−0.4
−0.4
−0.2
0.0
0.2
0.4
Oslo2
cor = −0.039

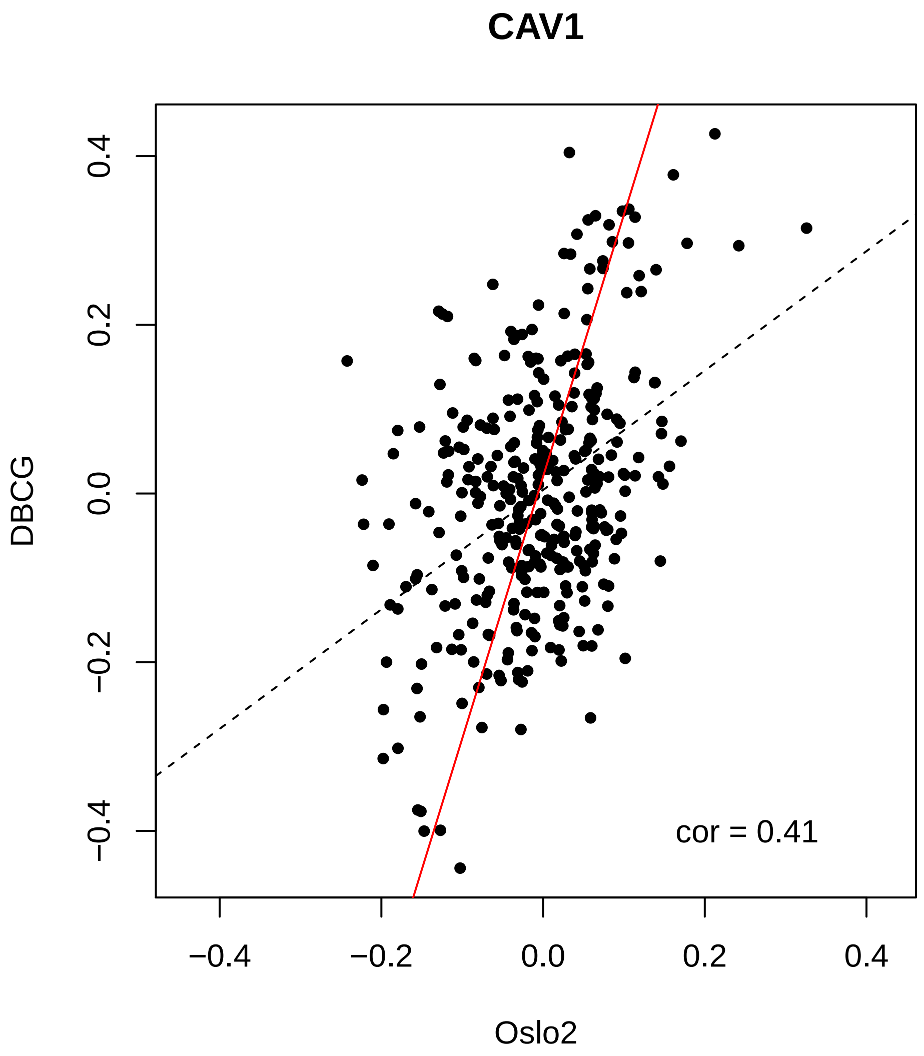

CAV1
DBCG
Oslo2
cor = 0.41
0.4
0.2
0.0
−0.2
−0.4
−0.4
−0.2
0.0
0.2
0.4

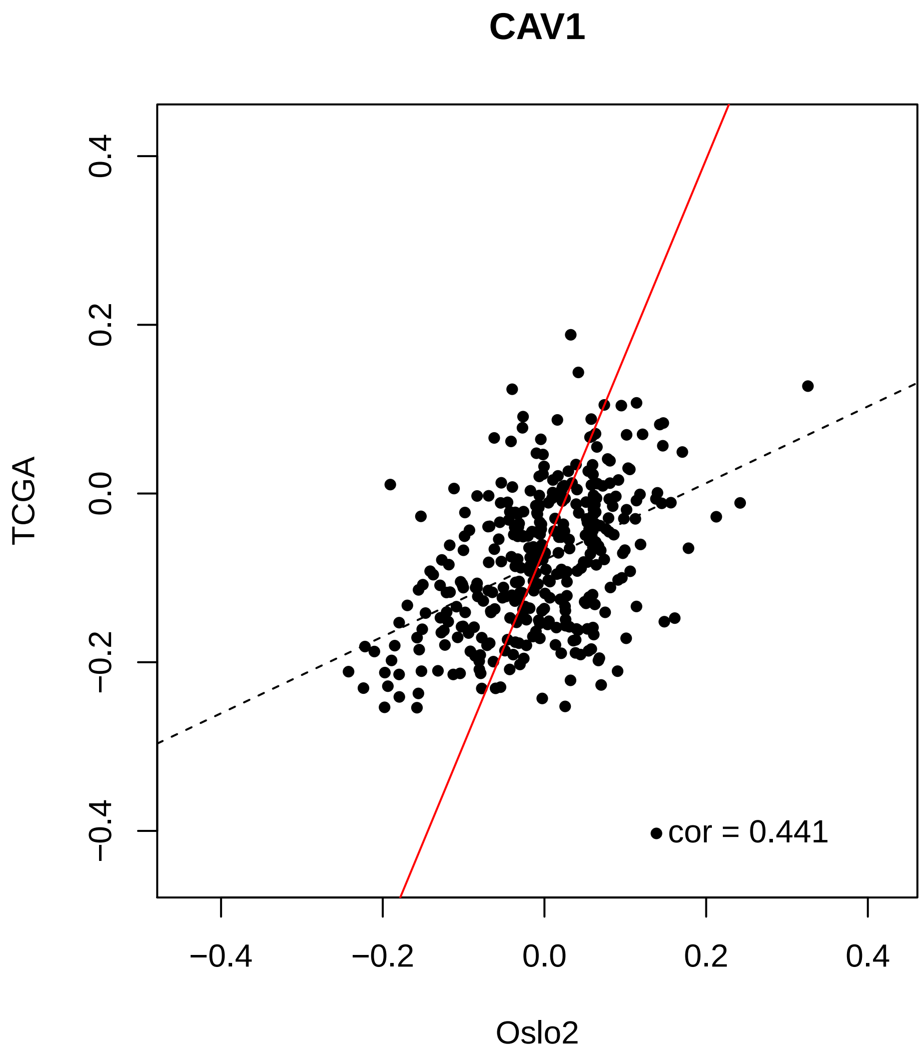

CAV1
TCGA
Oslo2
cor = 0.441
0.4
0.2
0.0
−0.2
−0.4
−0.4
−0.2
0.0
0.2
0.4

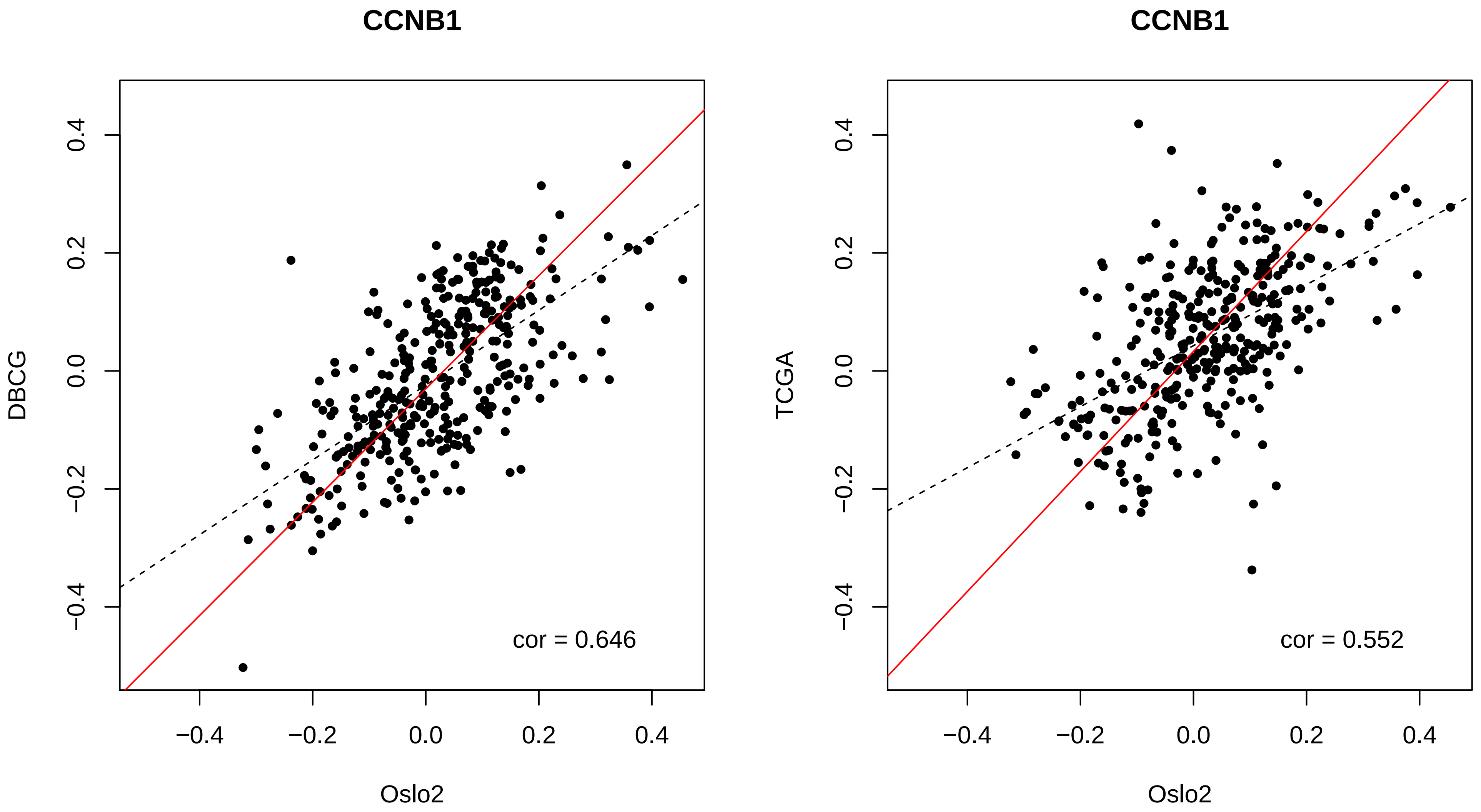
CCNB1
DBCG
Oslo2
cor = 0.646
−0.4
−0.2
0.0
0.2
0.4
CCNB1
TCGA
Oslo2
cor = 0.552

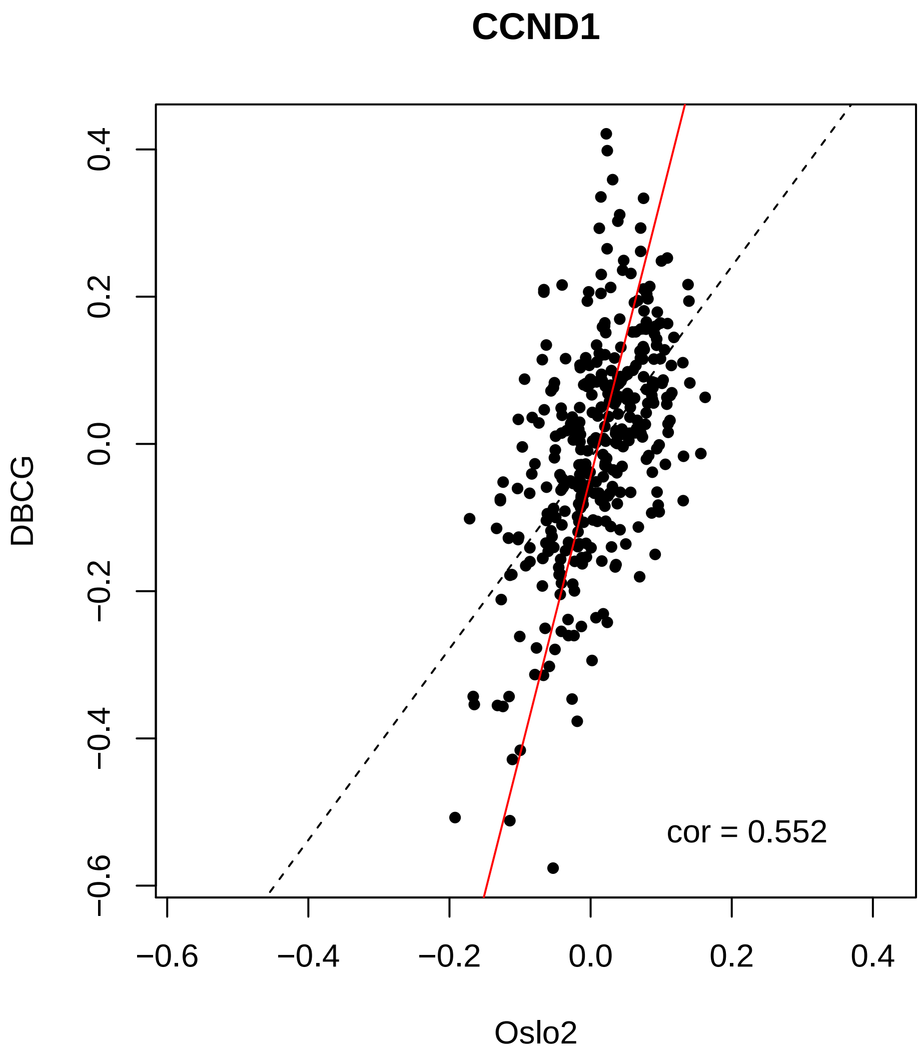

CCND1
DBCG
Oslo2
cor = 0.552
0.4
0.2
0.0
−0.2
−0.4
−0.6
−0.6
−0.4
−0.2
0.0
0.2
0.4

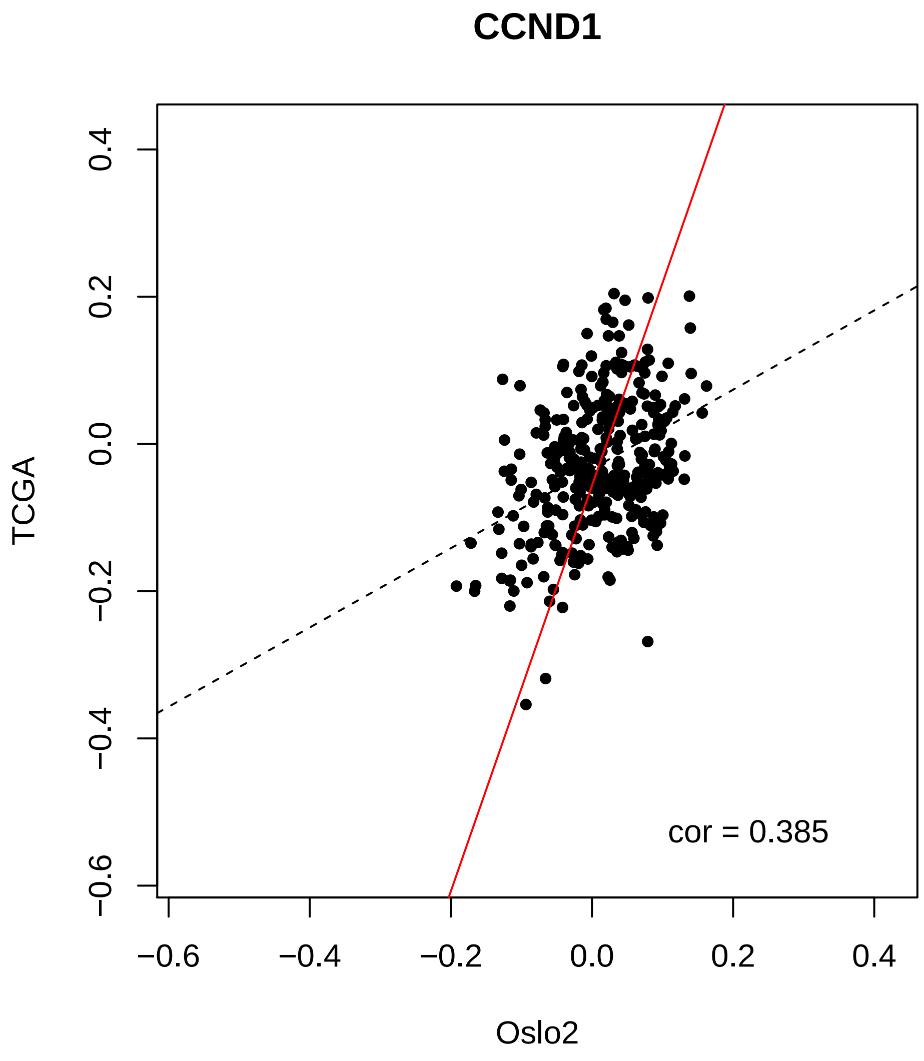

CCND1
TCGA
Oslo2
cor = 0.385
0.4
0.2
0.0
−0.2
−0.4
−0.6
−0.6
−0.4
−0.2
0.0
0.2
0.4

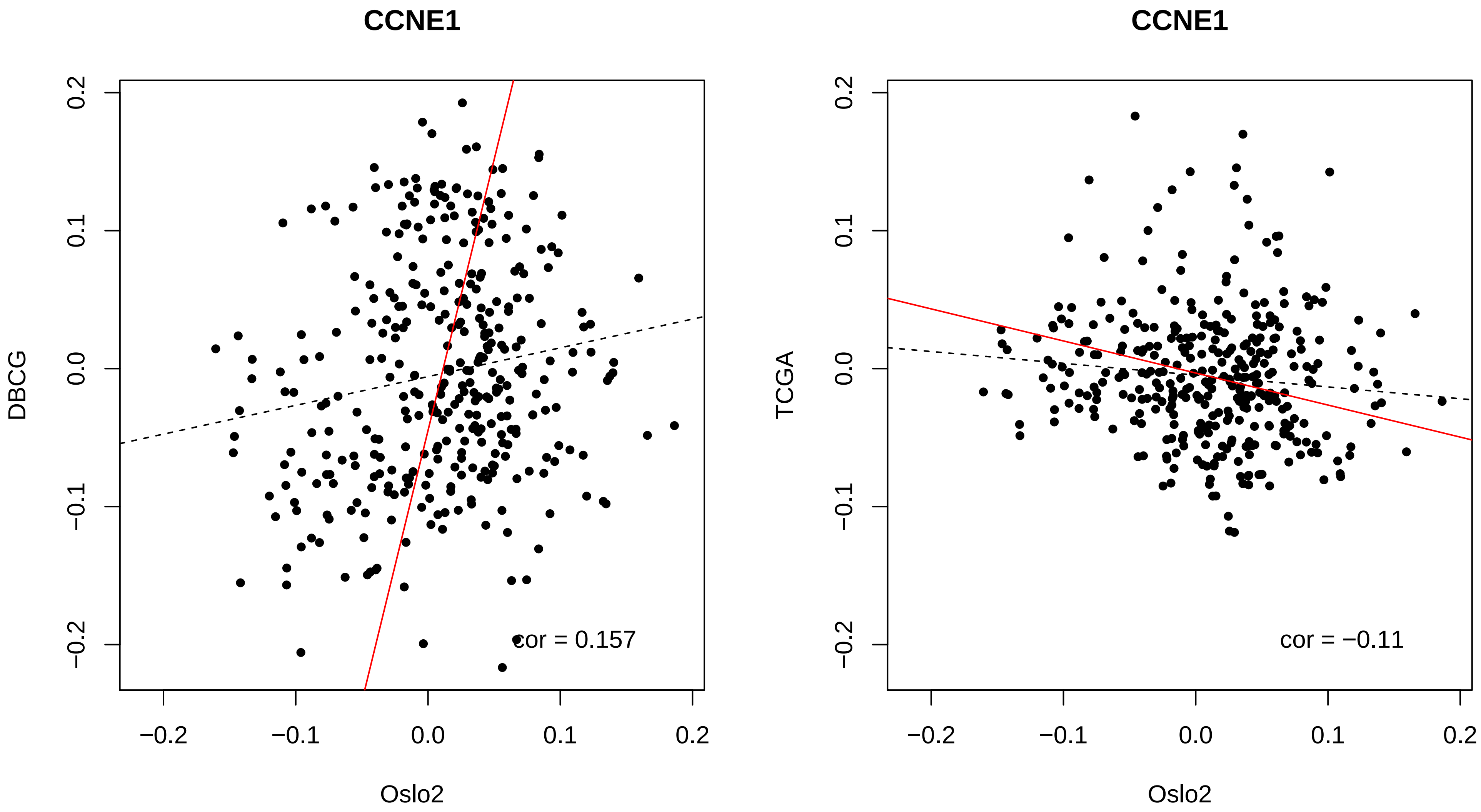
CCNE1
DBCG
Oslo2
cor = 0.157
0.2
0.1
0.0
−0.1
−0.2
CCNE1
TCGA
Oslo2
cor = −0.11

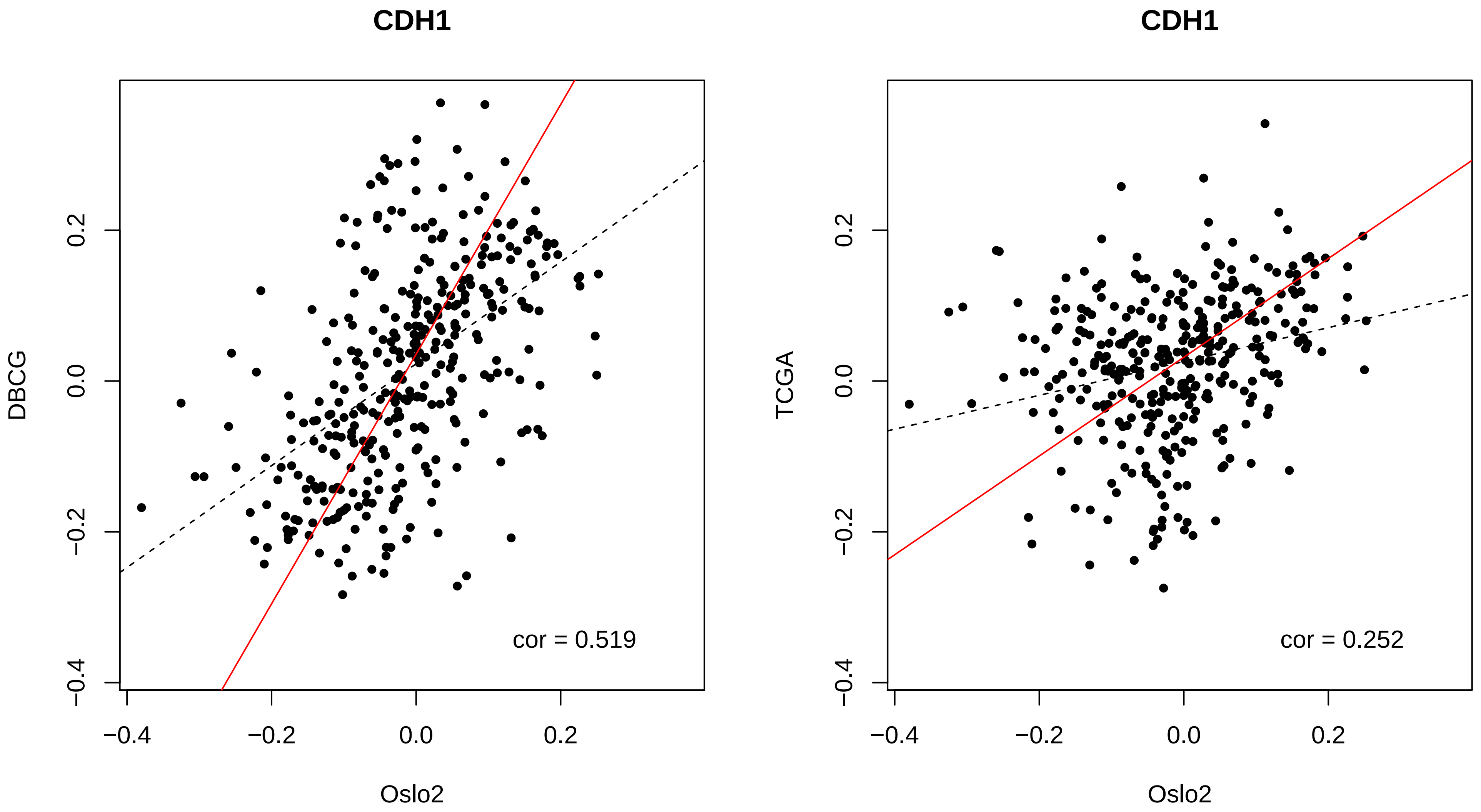
CDH1
DBCG
Oslo2
cor = 0.519
CDH1
TCGA
Oslo2
cor = 0.252
−0.4
−0.2
0.0
0.2

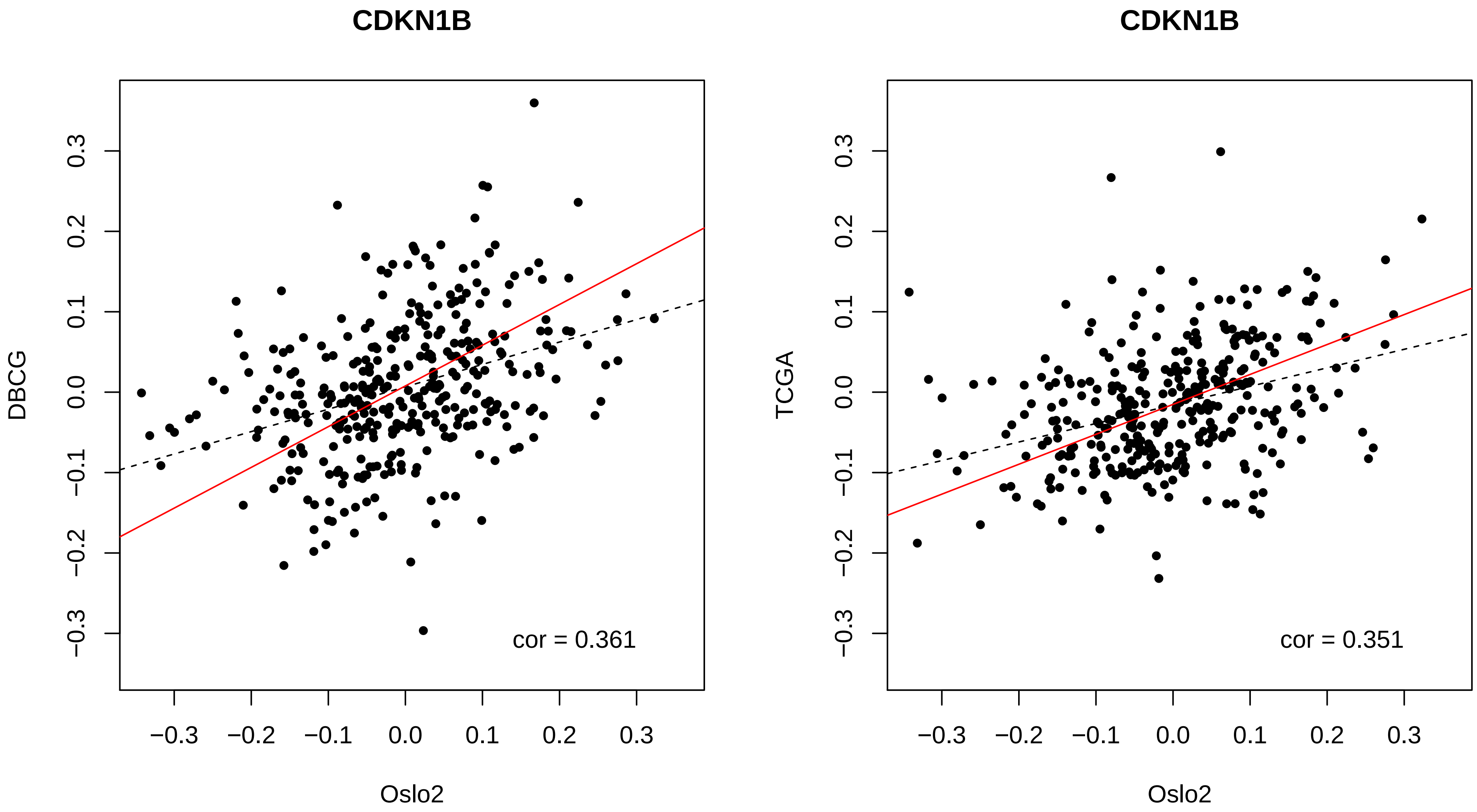
CDKN1B
DBCG
Oslo2
cor = 0.361
0.3
0.2
0.1
0.0
−0.1
−0.2
−0.3
CDKN1B
TCGA
Oslo2
cor = 0.351

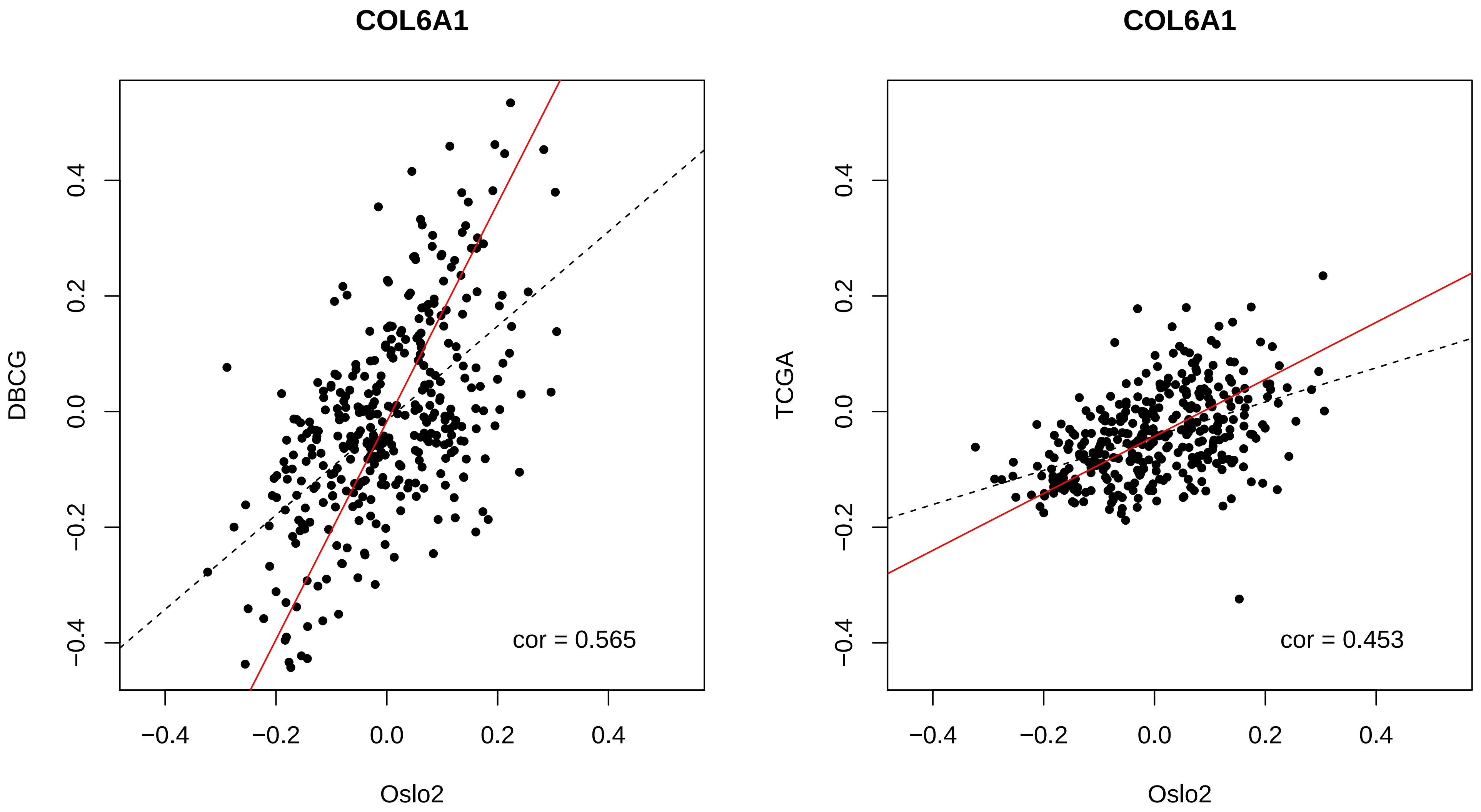
COL6A1
DBCG
Oslo2
cor = 0.565
COL6A1
TCGA
Oslo2
cor = 0.453
−0.4
−0.2
0.0
0.2
0.4

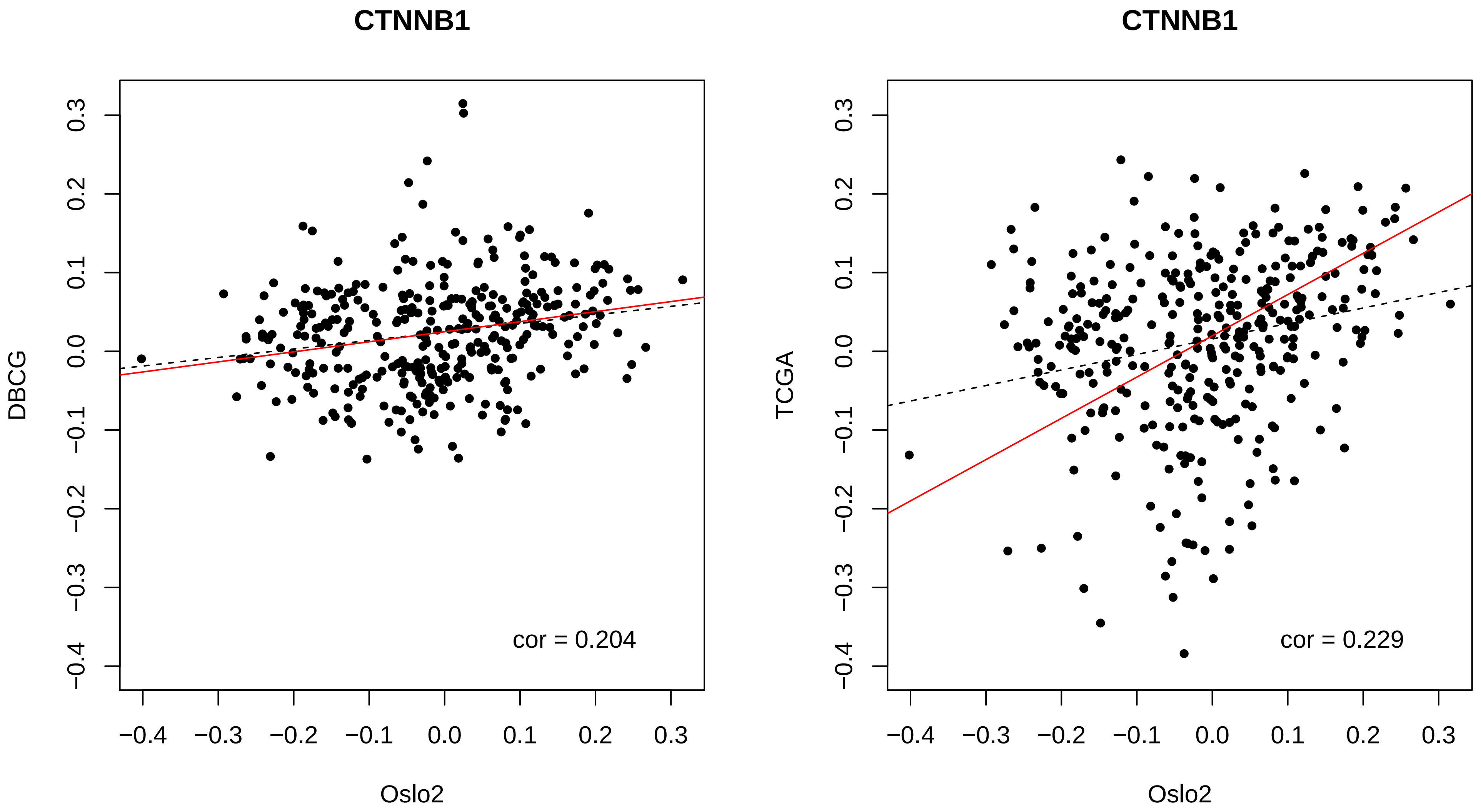

CTNNB1
DBCG
Oslo2
cor = 0.204
CTNNB1
TCGA
Oslo2
cor = 0.229

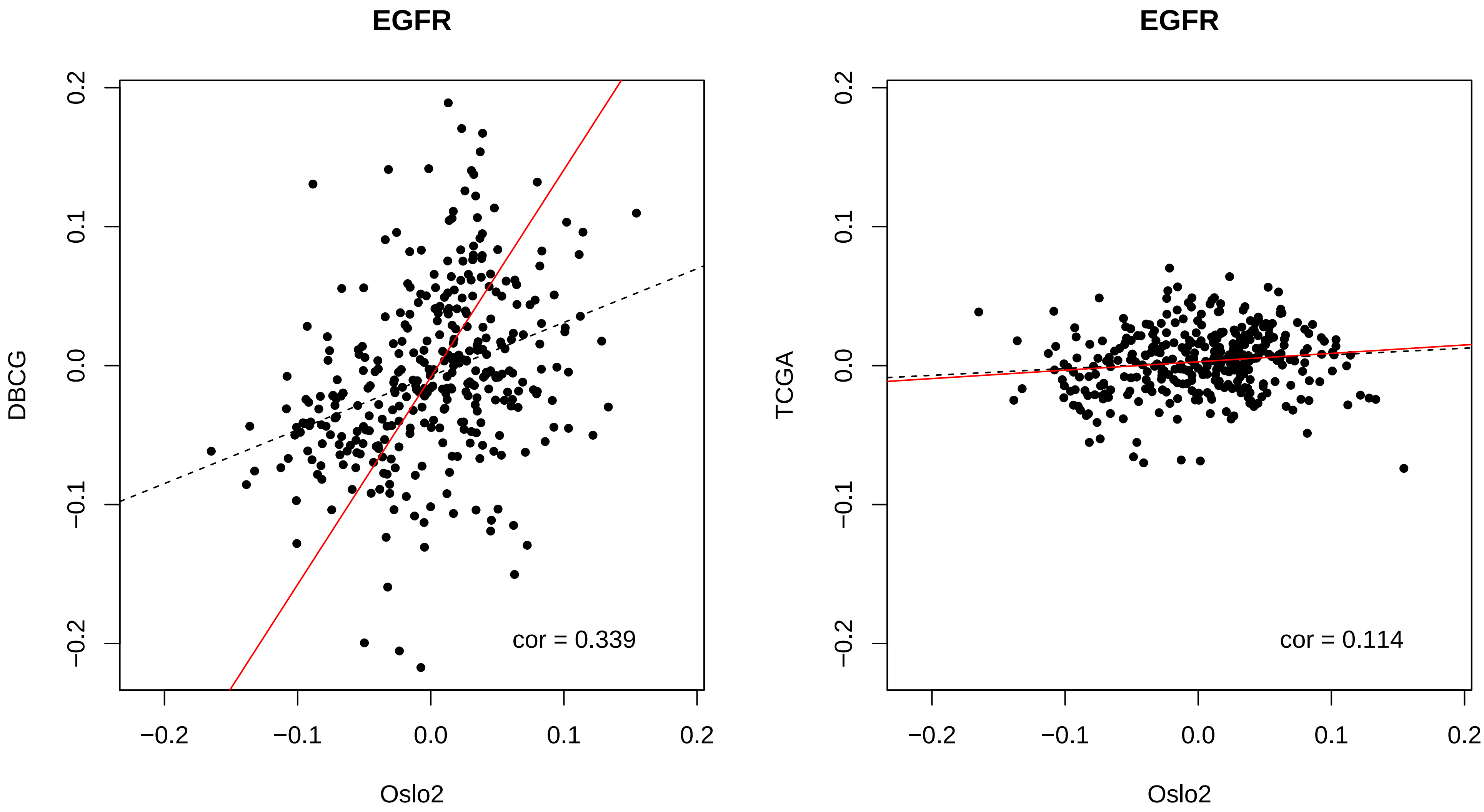
EGFR
DBCG
Oslo2
cor = 0.339
0.2
0.1
0.0
−0.1
−0.2
−0.2
−0.1
0.0
0.1
0.2
EGFR
TCGA
Oslo2
cor = 0.114

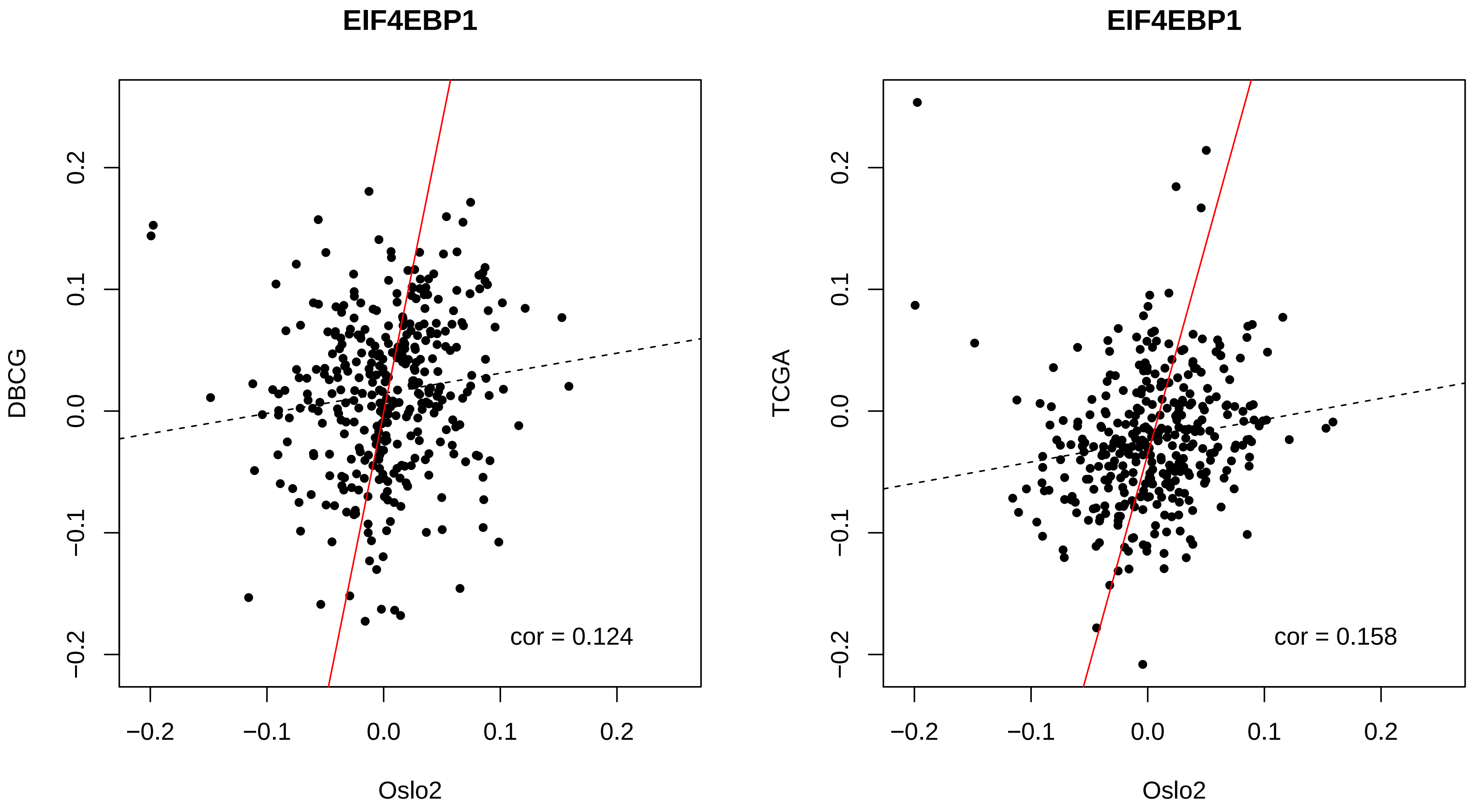

EIF4EBP1
DBCG
0.2
0.1
0.0
−0.1
−0.2
−0.2
−0.1
0.0
0.1
0.2
Oslo2
cor = 0.124
EIF4EBP1
TCGA
0.2
0.1
0.0
−0.1
−0.2
−0.2
−0.1
0.0
0.1
0.2
Oslo2
cor = 0.158

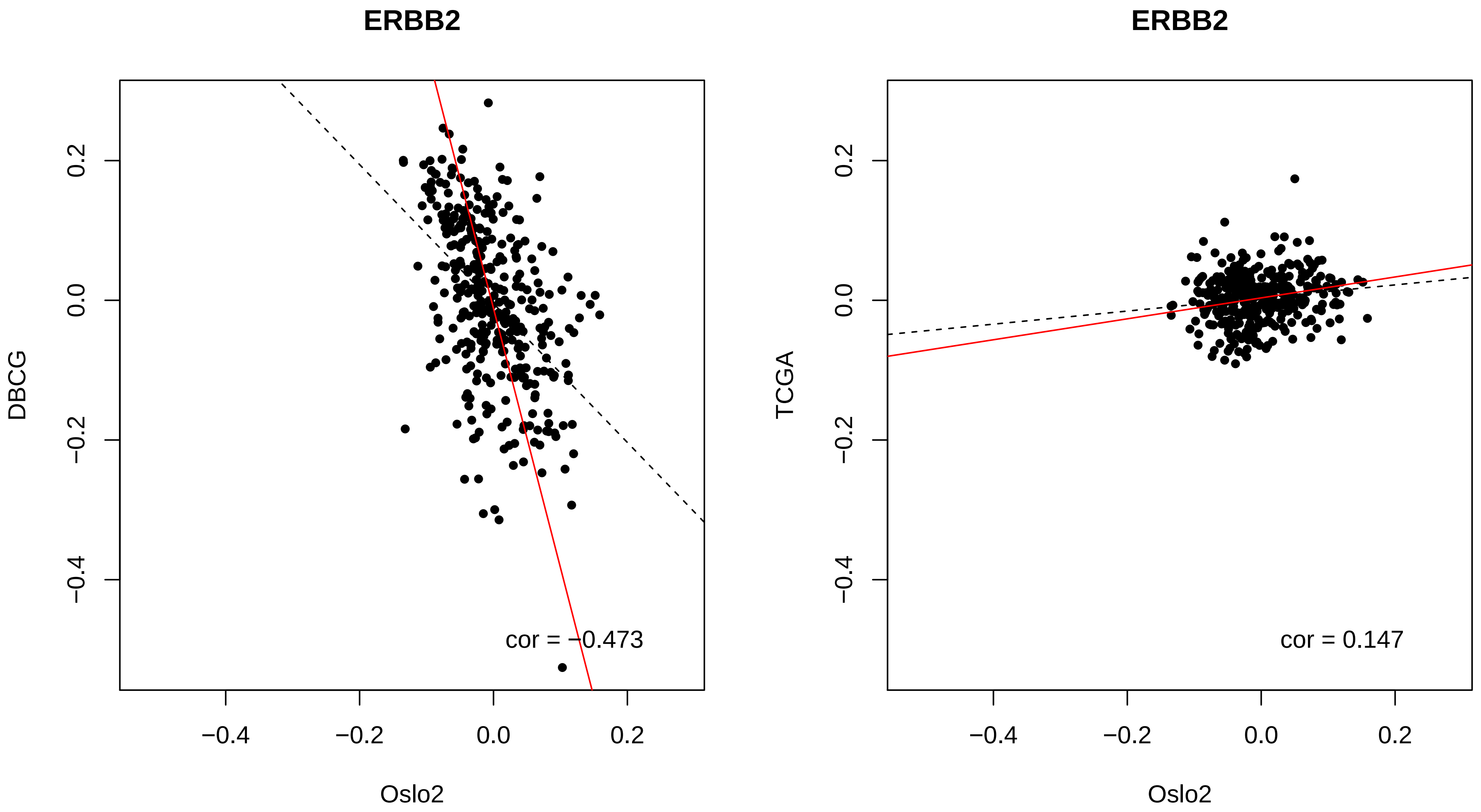

ERBB2
DBCG
Oslo2
cor = −0.473
0.2
0.0
−0.2
−0.4
ERBB2
TCGA
Oslo2
cor = 0.147

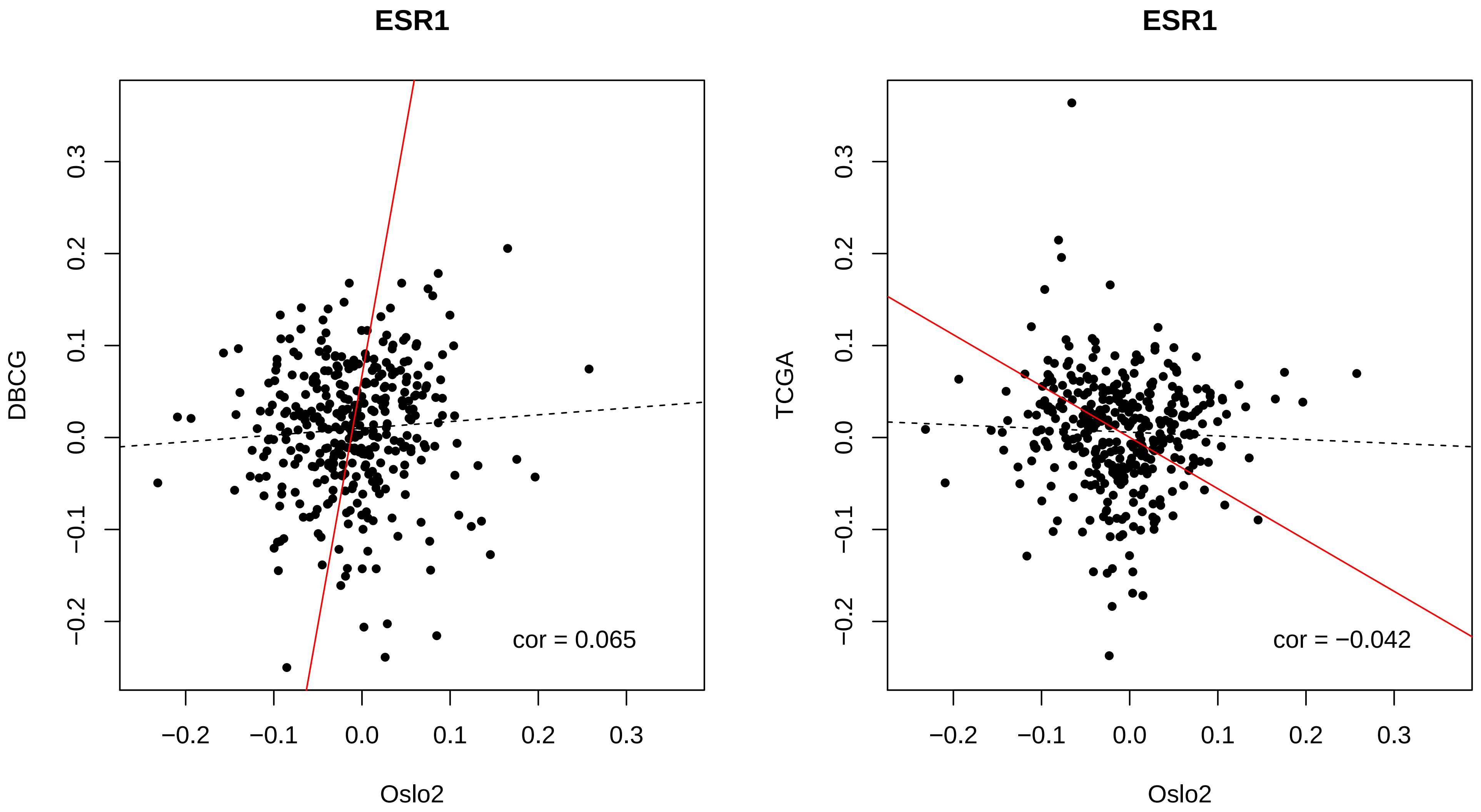
ESR1
DBCG
Oslo2
cor = 0.065
−0.2
−0.1
0.0
0.1
0.2
0.3
ESR1
TCGA
Oslo2
cor = −0.042

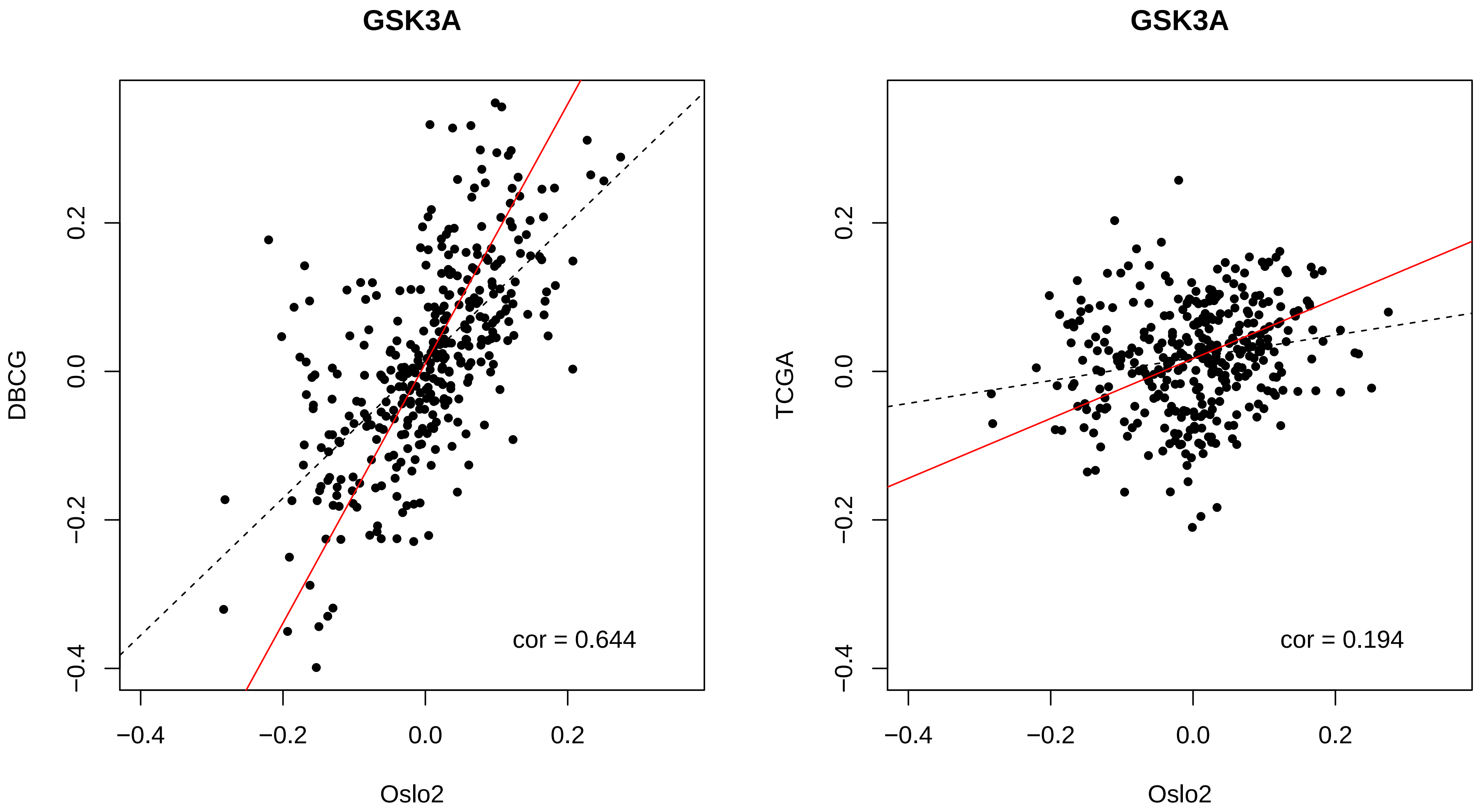
GSK3A
DBCG
Oslo2
cor = 0.644
−0.4
−0.2
0.0
0.2
GSK3A
TCGA
Oslo2
cor = 0.194

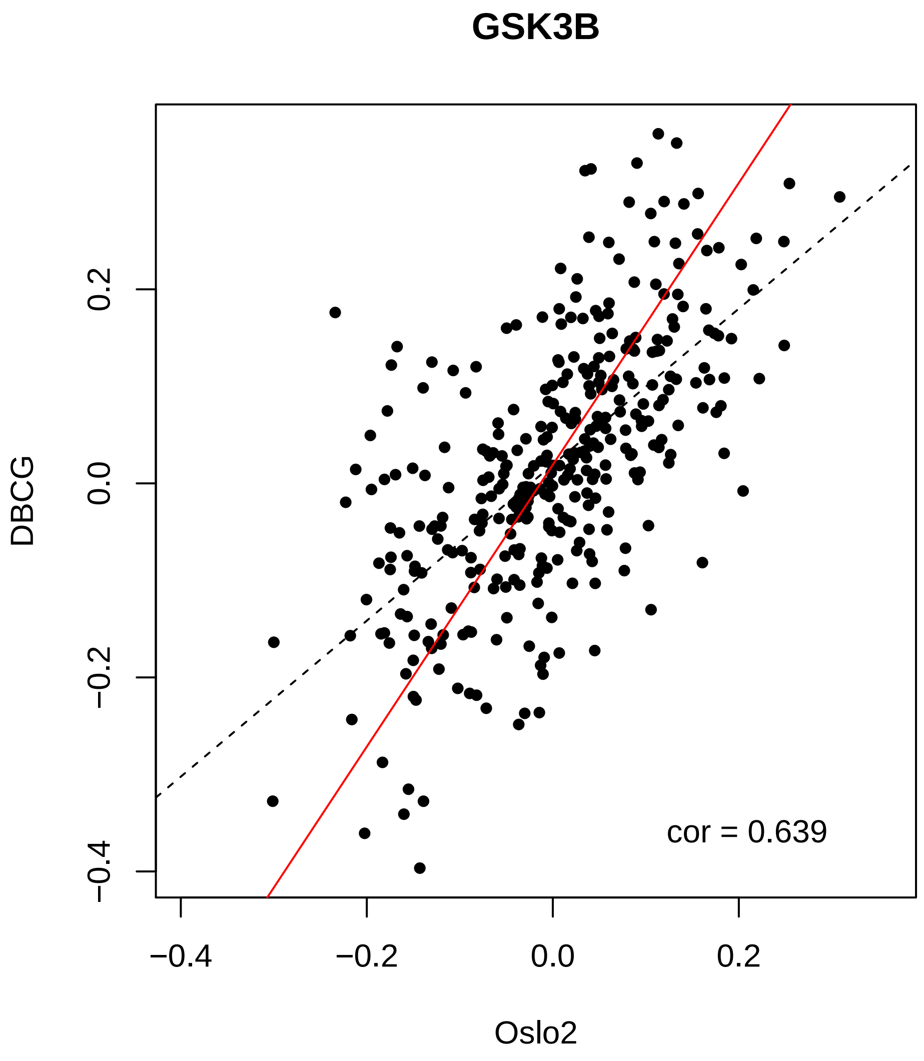
GSK3B
DBCG
Oslo2
cor = 0.639
0.2
0.0
−0.2
−0.4
−0.4
−0.2
0.0
0.2

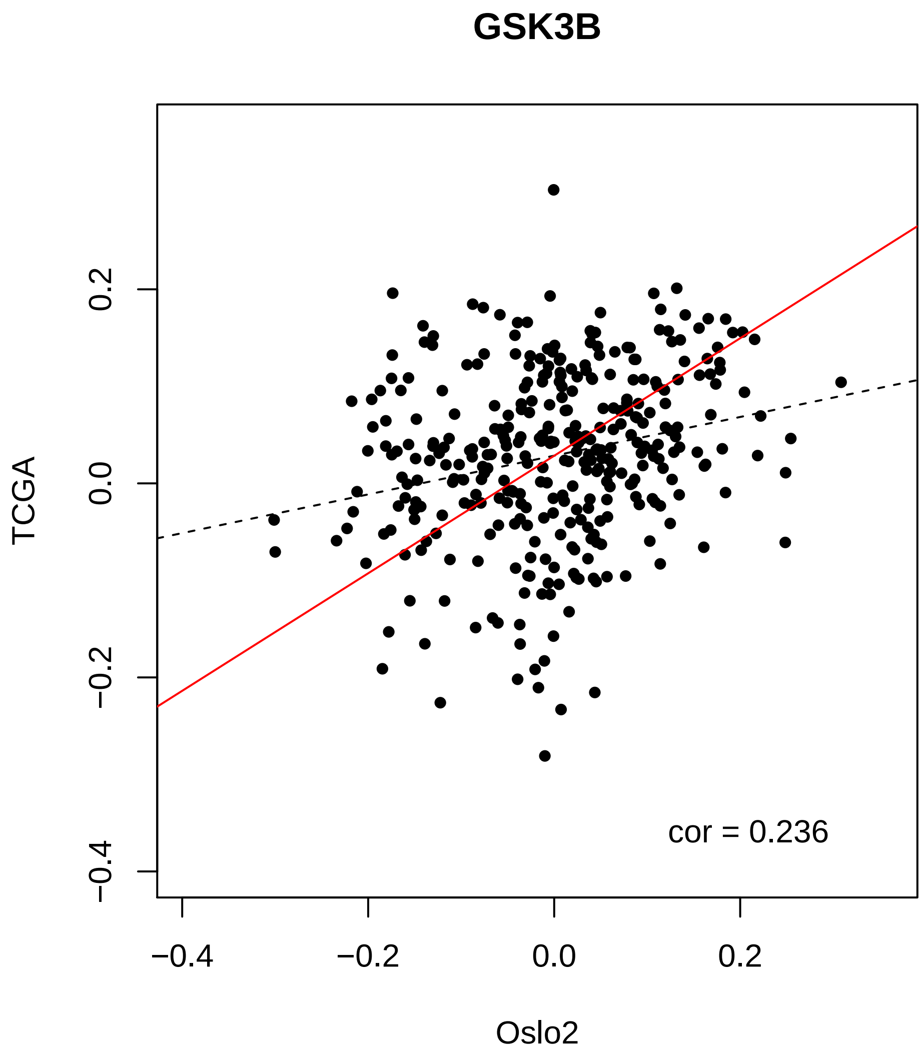
GSK3B
TCGA
Oslo2
cor = 0.236
0.2
0.0
−0.2
−0.4
−0.4
−0.2
0.0
0.2

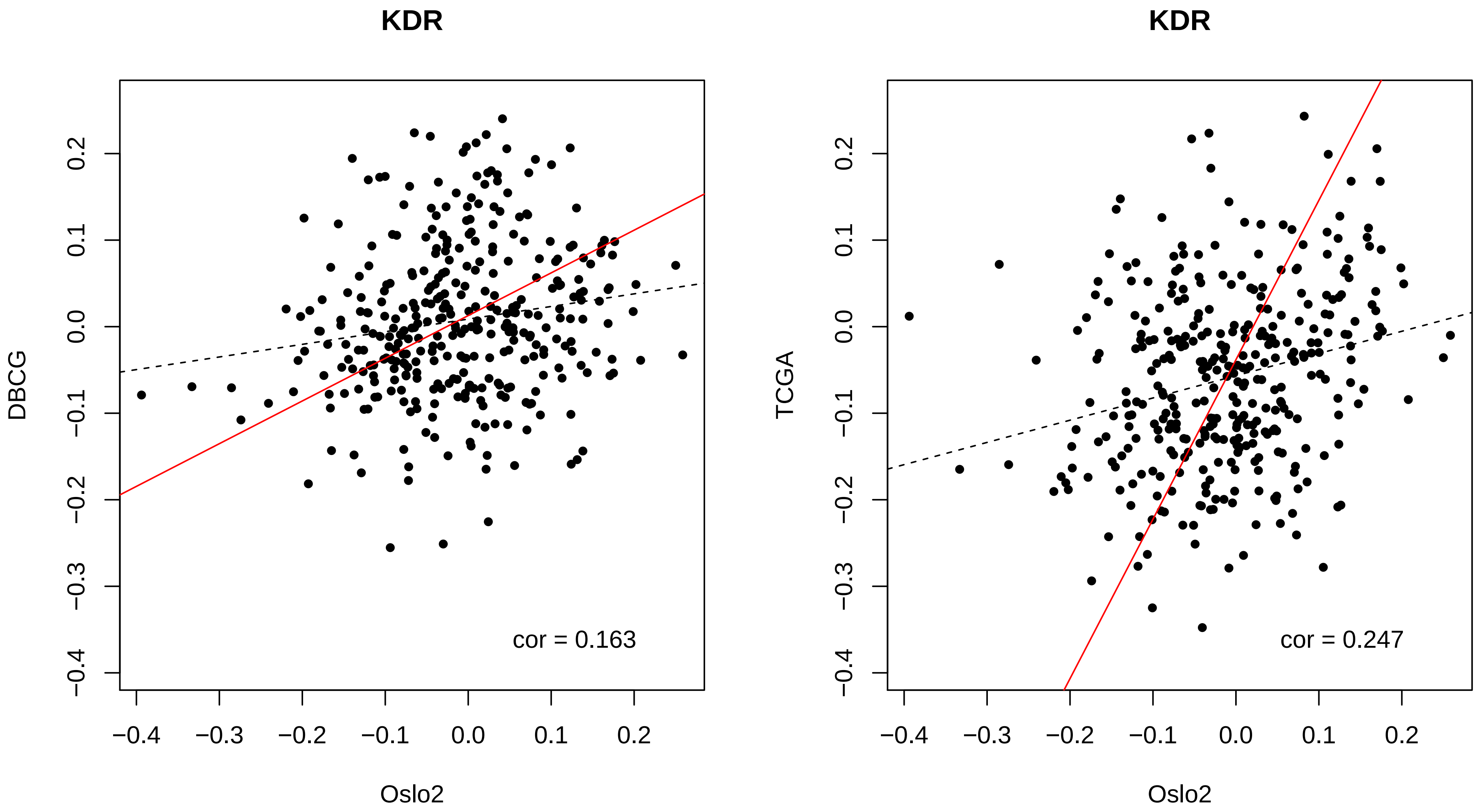
KDR
DBCG
Oslo2
cor = 0.163
KDR
TCGA
Oslo2
cor = 0.247
0.2
0.1
0.0
−0.1
−0.2
−0.3
−0.4
−0.4
−0.3
−0.2
−0.1
0.0
0.1
0.2

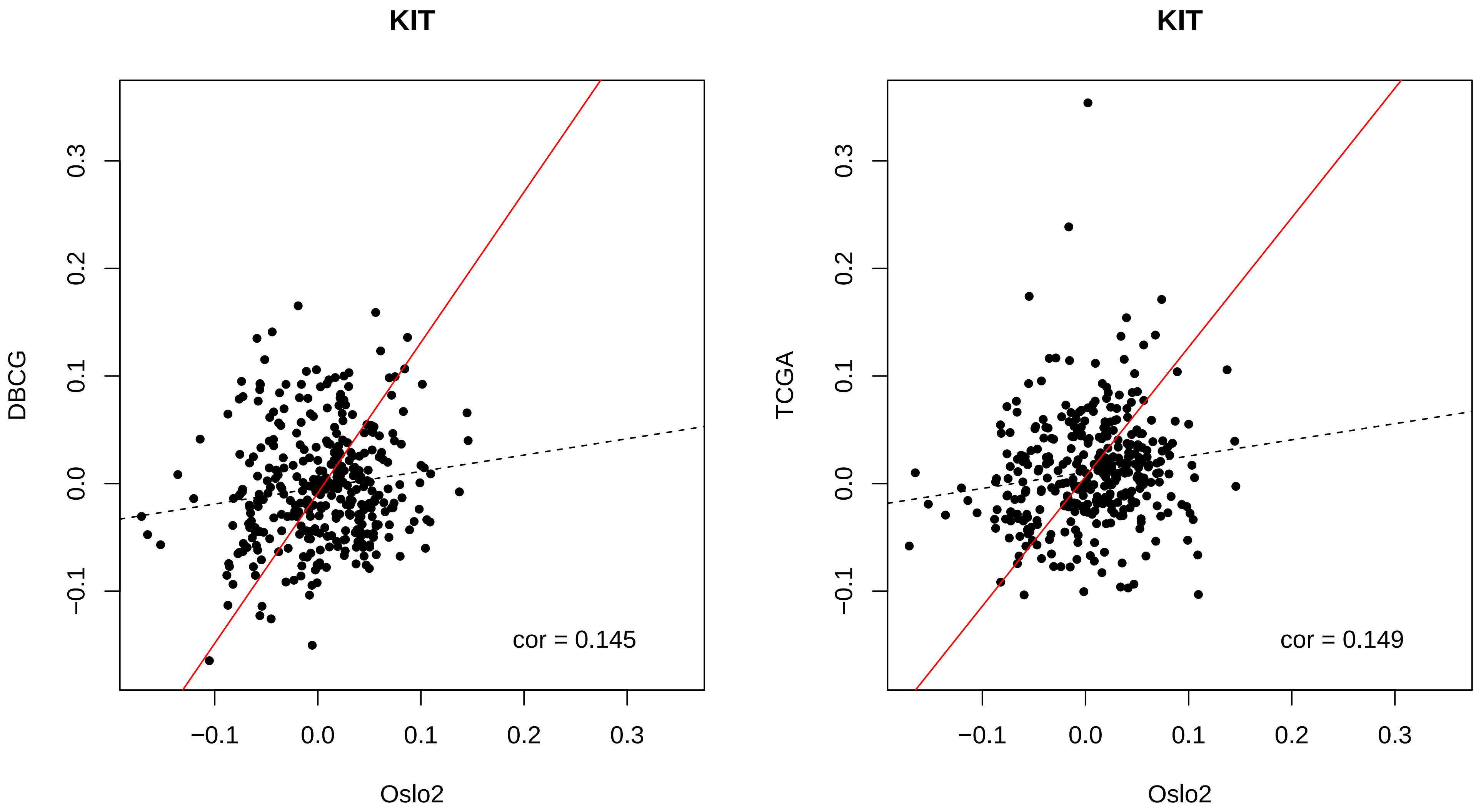
KIT
DBCG
Oslo2
cor = 0.145
−0.1
0.0
0.1
0.2
0.3
KIT
TCGA
Oslo2
cor = 0.149

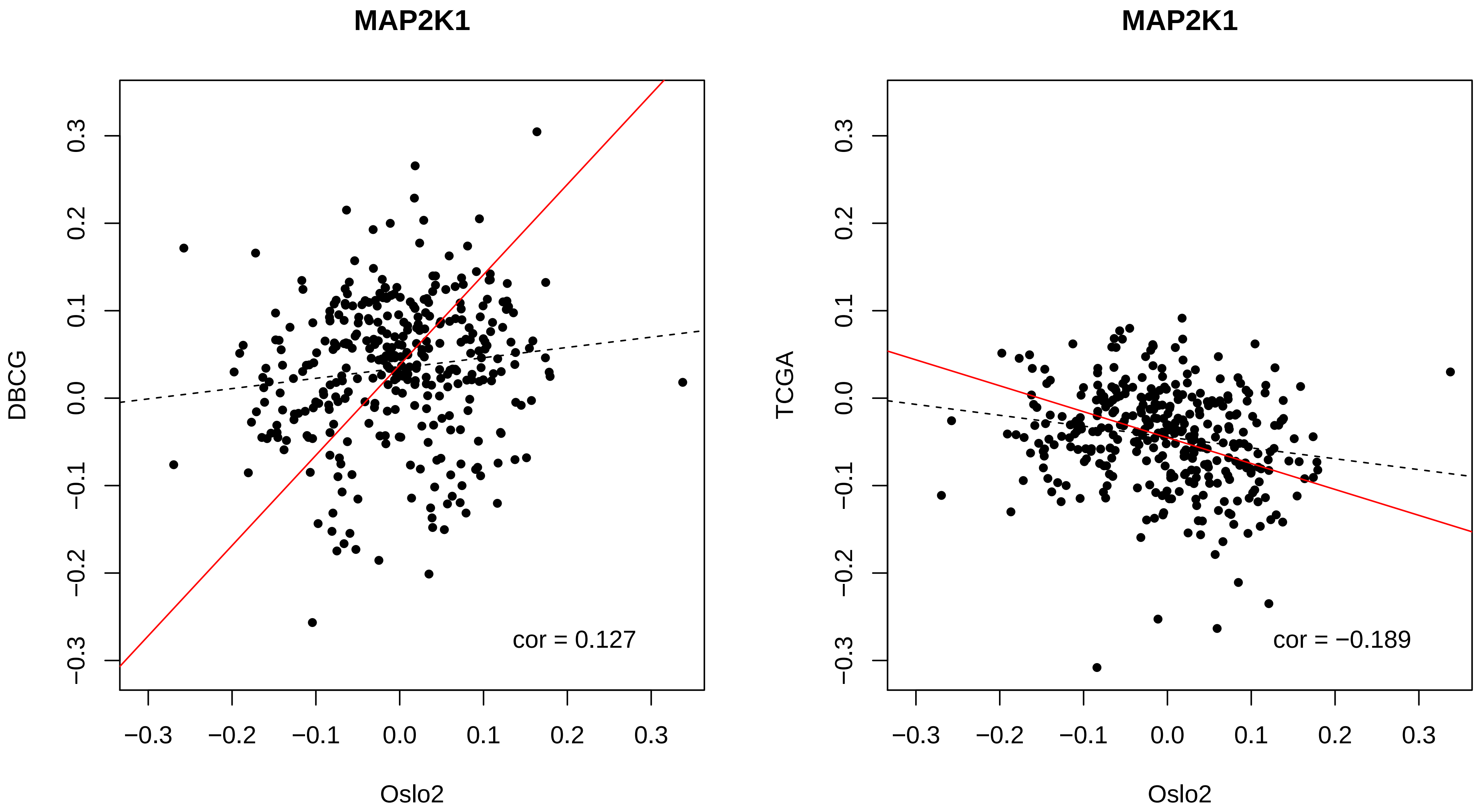

MAP2K1
DBCG
Oslo2
cor = 0.127
−0.3
−0.2
−0.1
0.0
0.1
0.2
0.3
MAP2K1
TCGA
Oslo2
cor = −0.189
−0.3
−0.2
−0.1
0.0
0.1
0.2
0.3

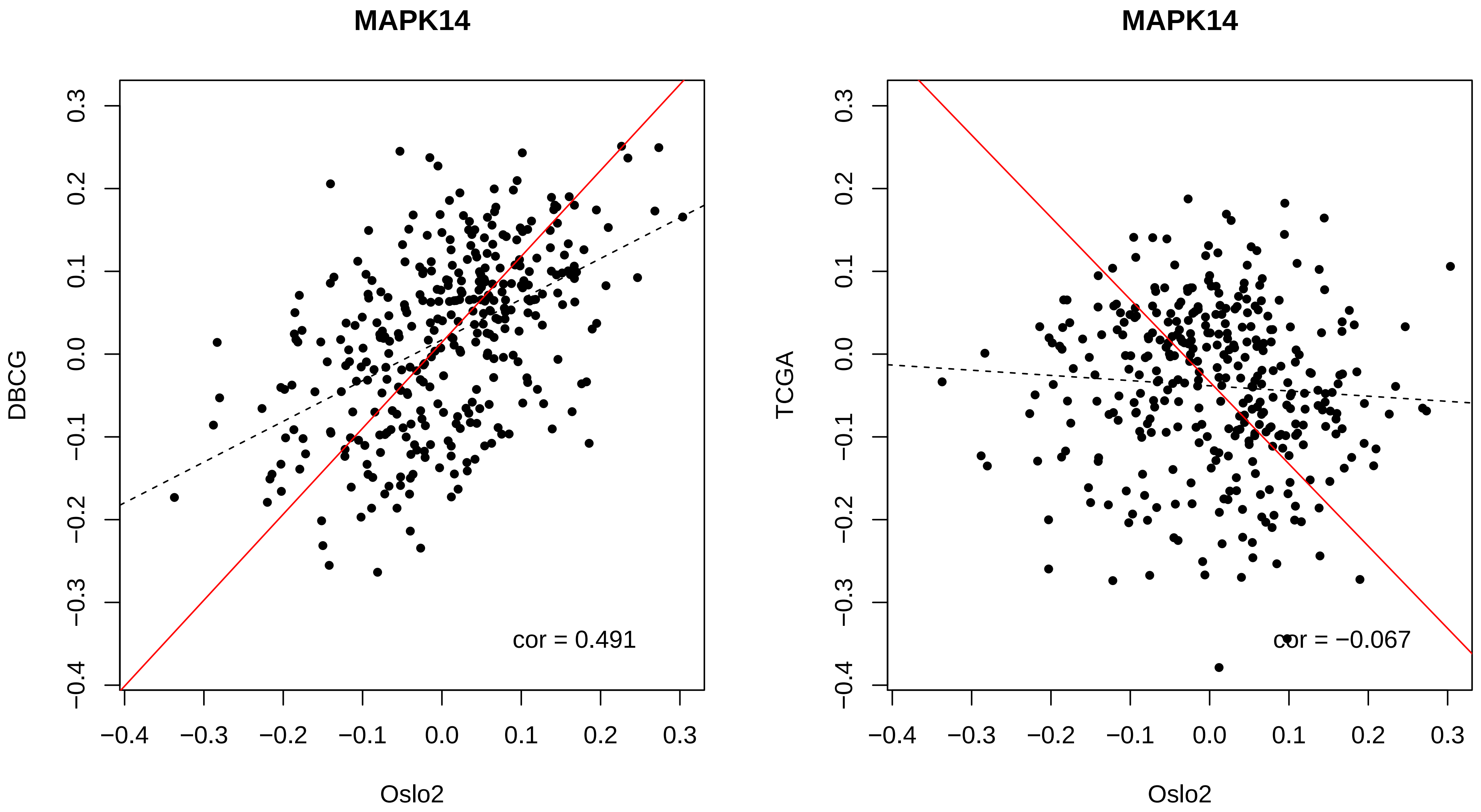
MAPK14
DBCG
Oslo2
cor = 0.491
MAPK14
TCGA
Oslo2
cor = −0.067
0.3
0.2
0.1
0.0
−0.1
−0.2
−0.3
−0.4

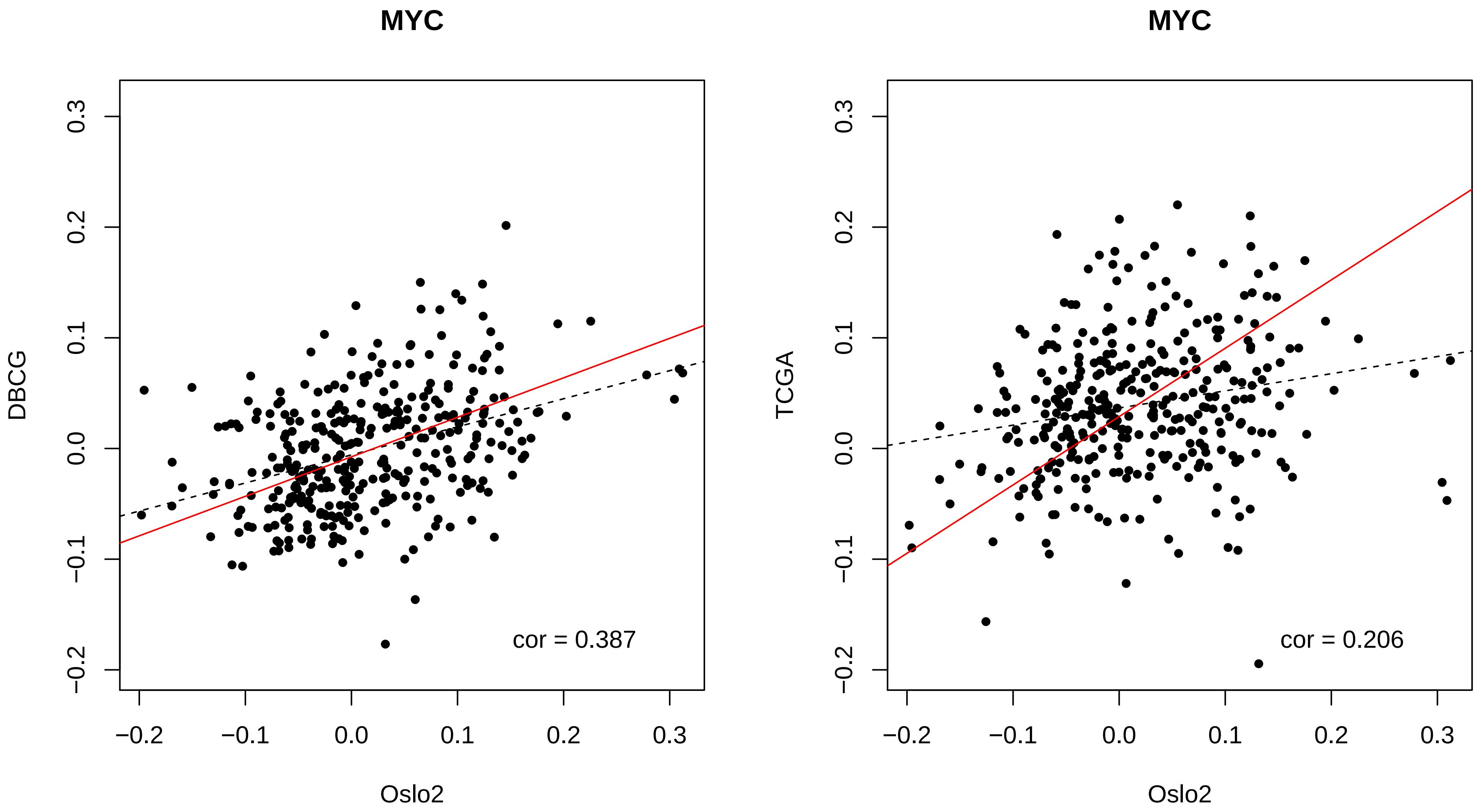
MYC
DBCG
Oslo2
cor = 0.387
MYC
TCGA
Oslo2
cor = 0.206
−0.2
−0.1
0.0
0.1
0.2
0.3

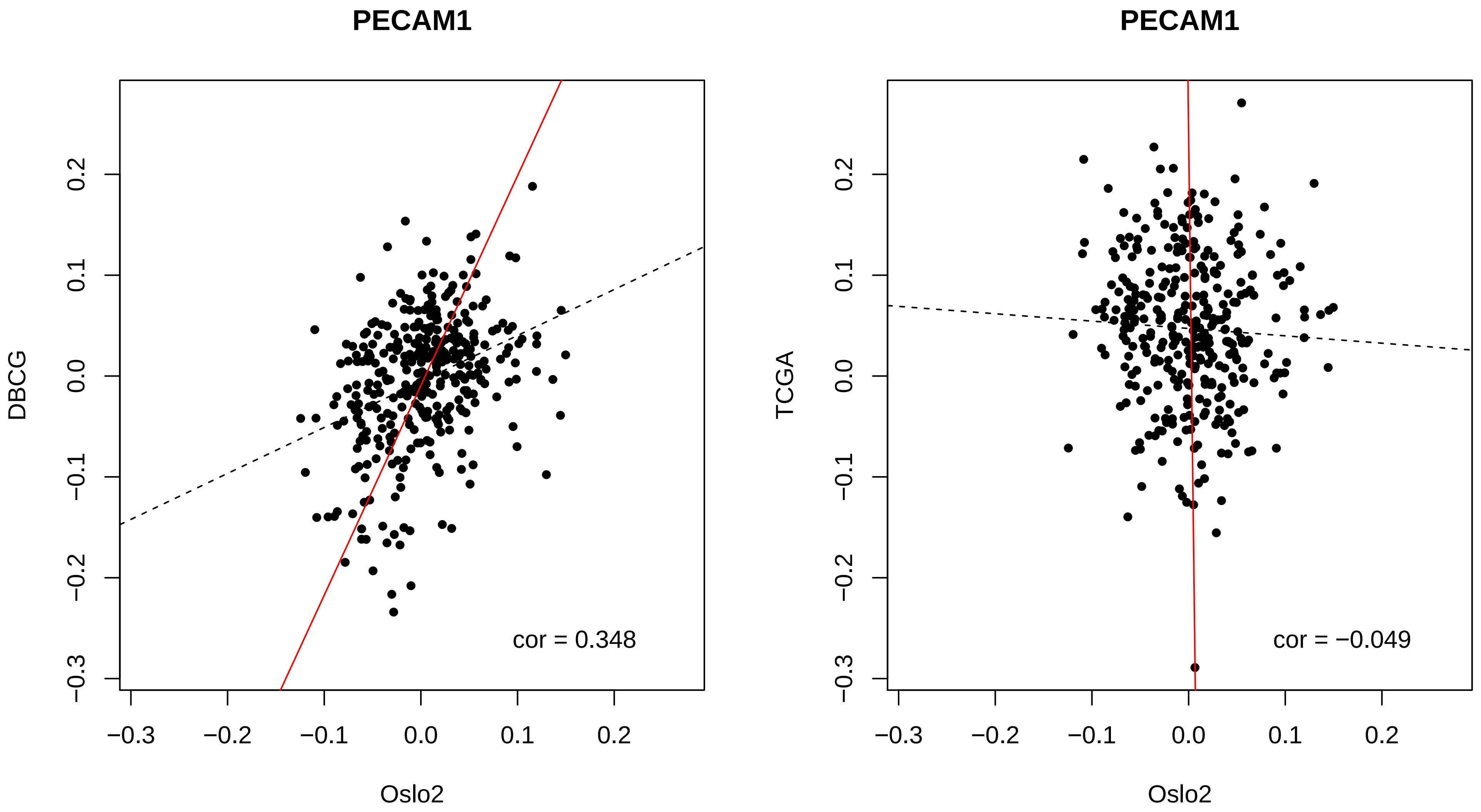
PECAM1
DBCG
Oslo2
cor = 0.348
PECAM1
TCGA
Oslo2
cor = −0.049
−0.3
−0.2
−0.1
0.0
0.1
0.2

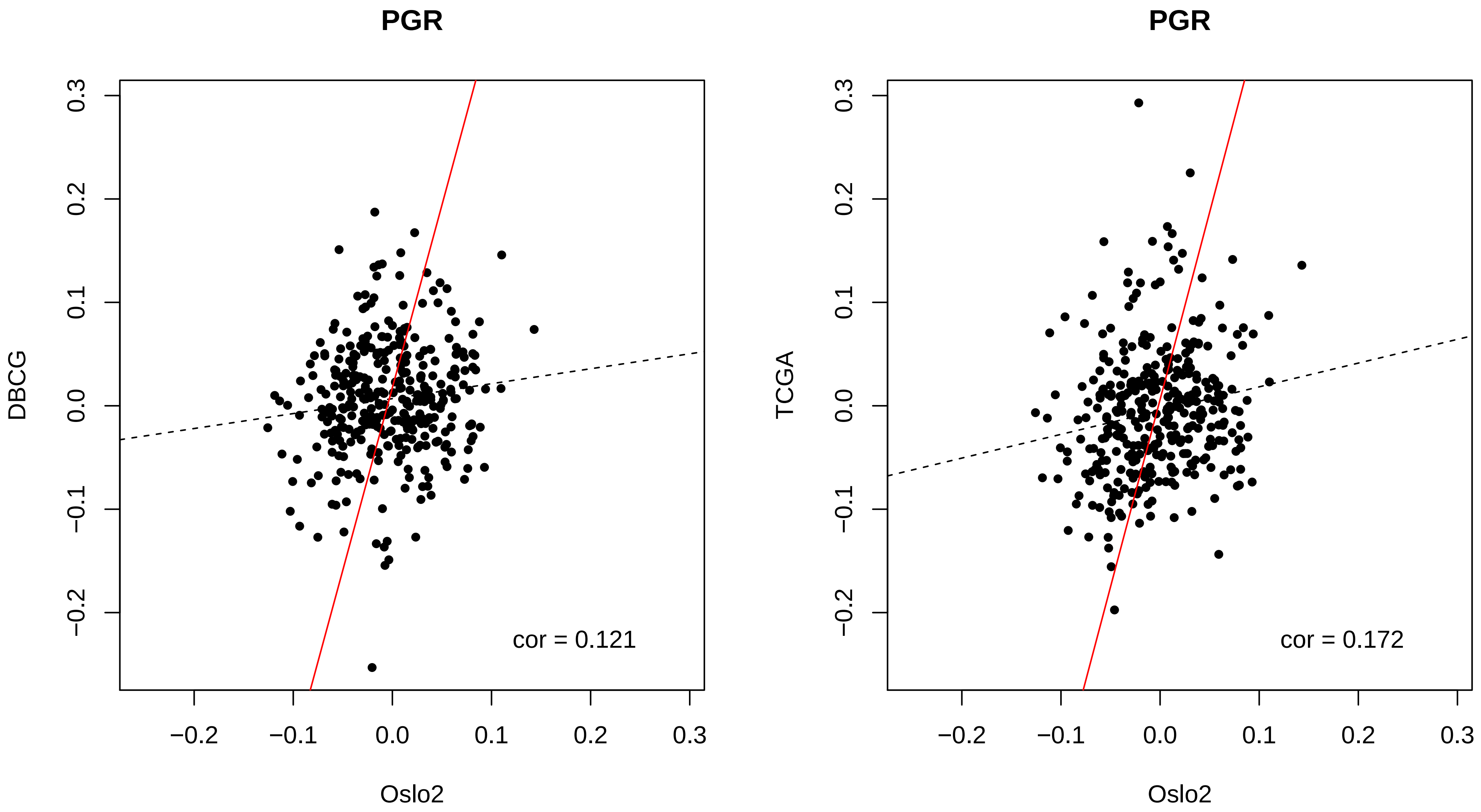
PGR
DBCG
Oslo2
cor = 0.121
PGR
TCGA
Oslo2
cor = 0.172
0.3
0.2
0.1
0.0
−0.1
−0.2
−0.2
−0.1
0.0
0.1
0.2
0.3

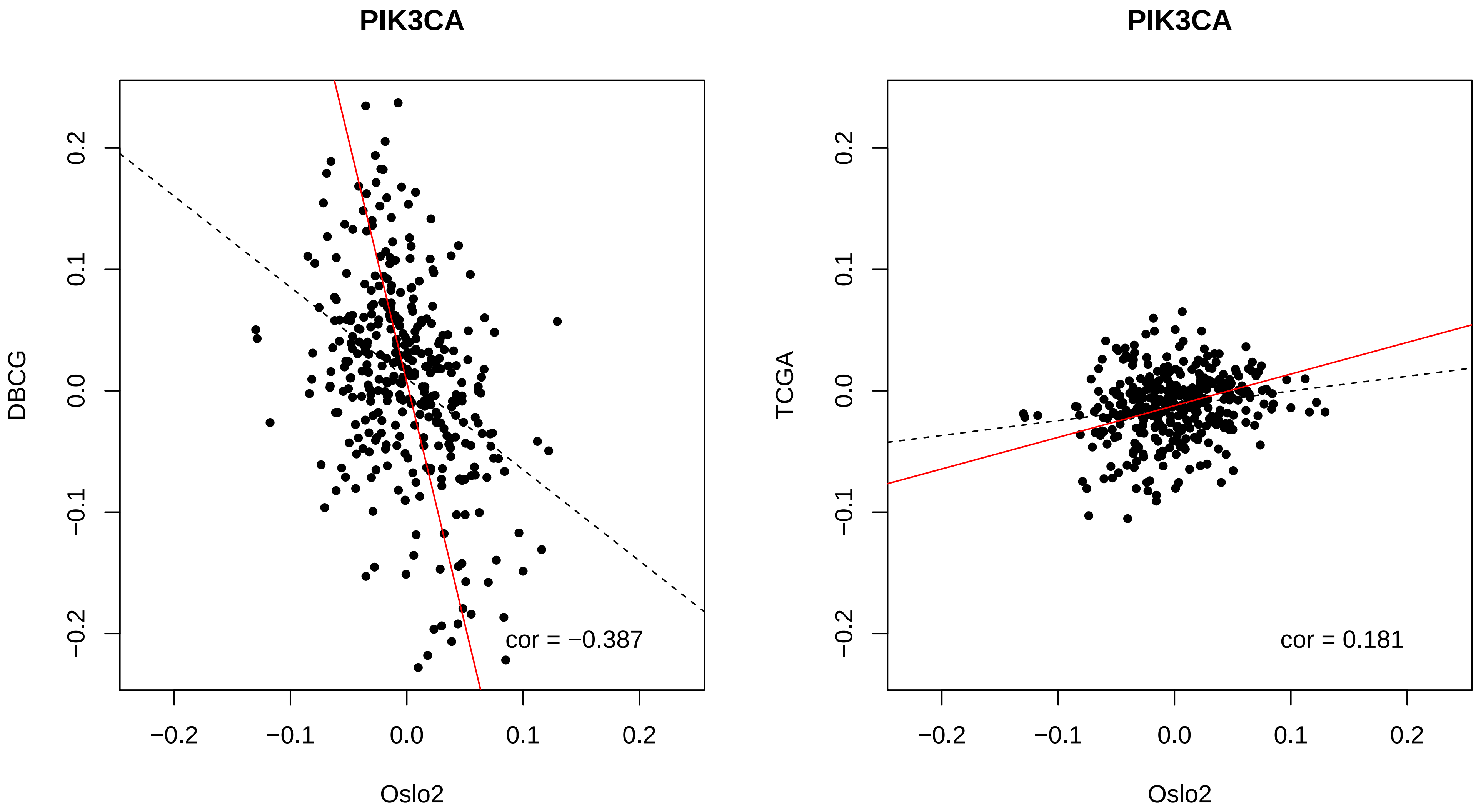
PIK3CA
DBCG
Oslo2
cor = −0.387
−0.2
−0.1
0.0
0.1
0.2
PIK3CA
TCGA
Oslo2
cor = 0.181

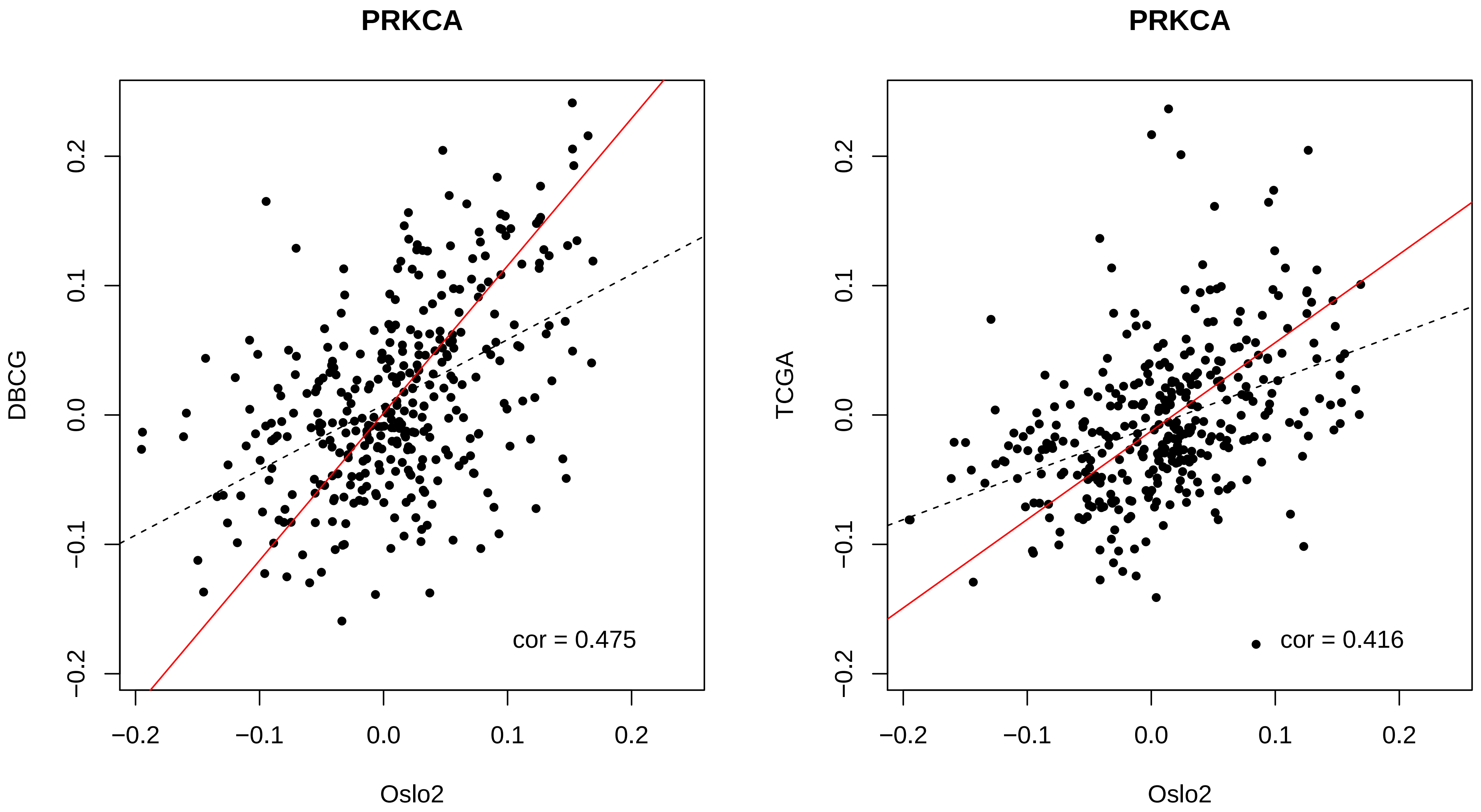
PRKCA
DBCG
Oslo2
cor = 0.475
−0.2
−0.1
0.0
0.1
0.2
PRKCA
TCGA
Oslo2
cor = 0.416

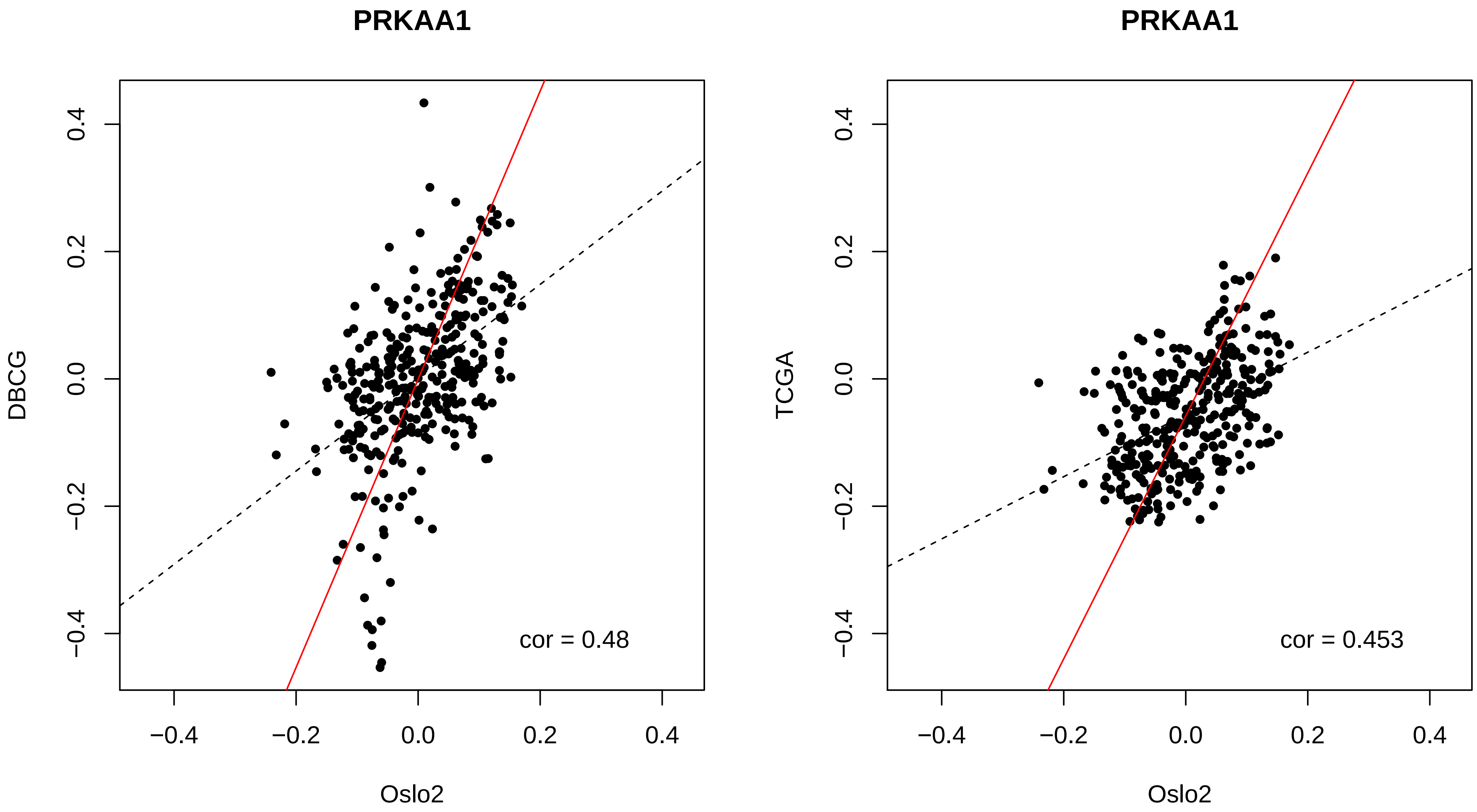

PRKAA1
DBCG
Oslo2
cor = 0.48
0.4
0.2
0.0
−0.2
−0.4
PRKAA1
TCGA
Oslo2
cor = 0.453

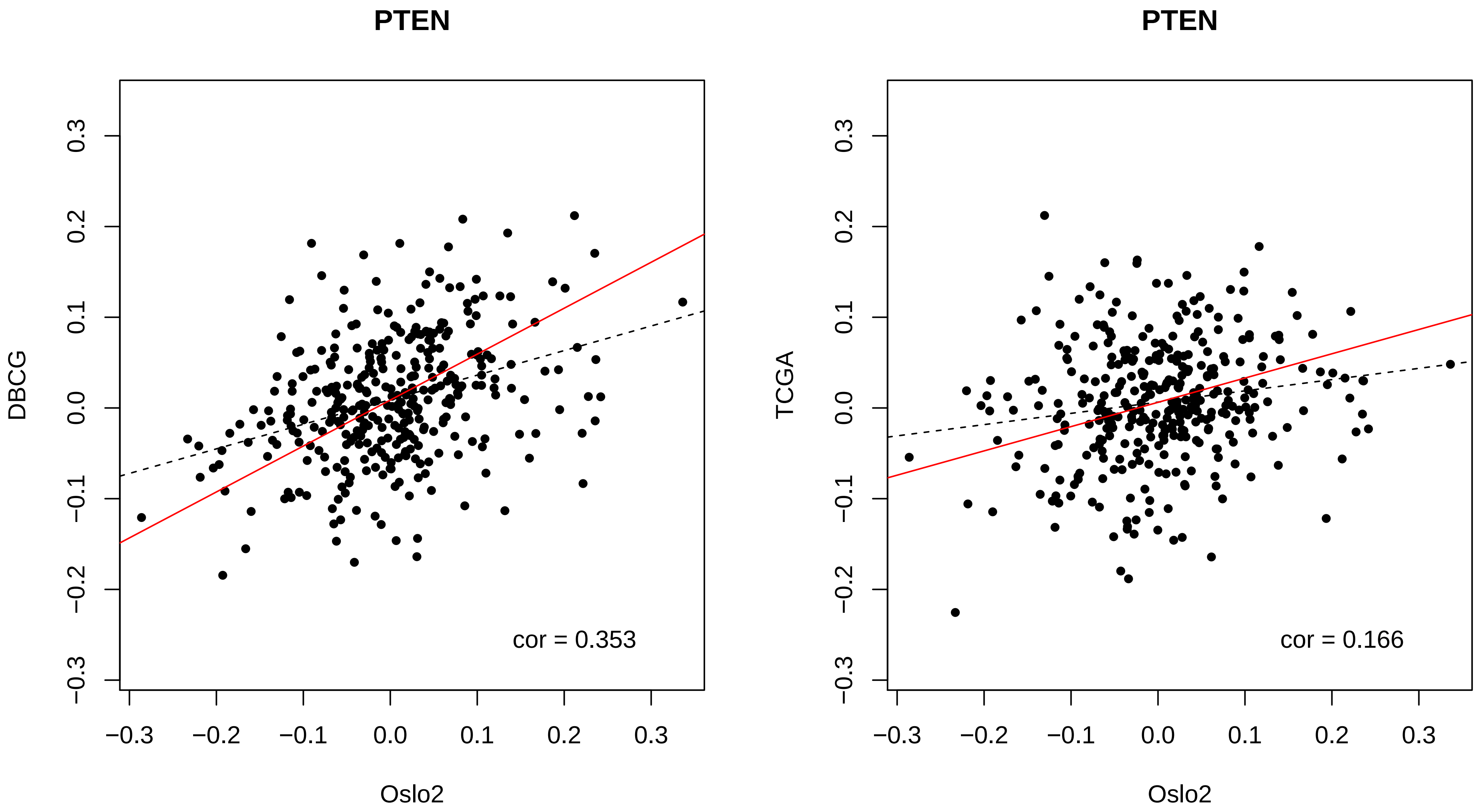
PTEN
DBCG
Oslo2
cor = 0.353
PTEN
TCGA
Oslo2
cor = 0.166
0.3
0.2
0.1
0.0
−0.1
−0.2
−0.3

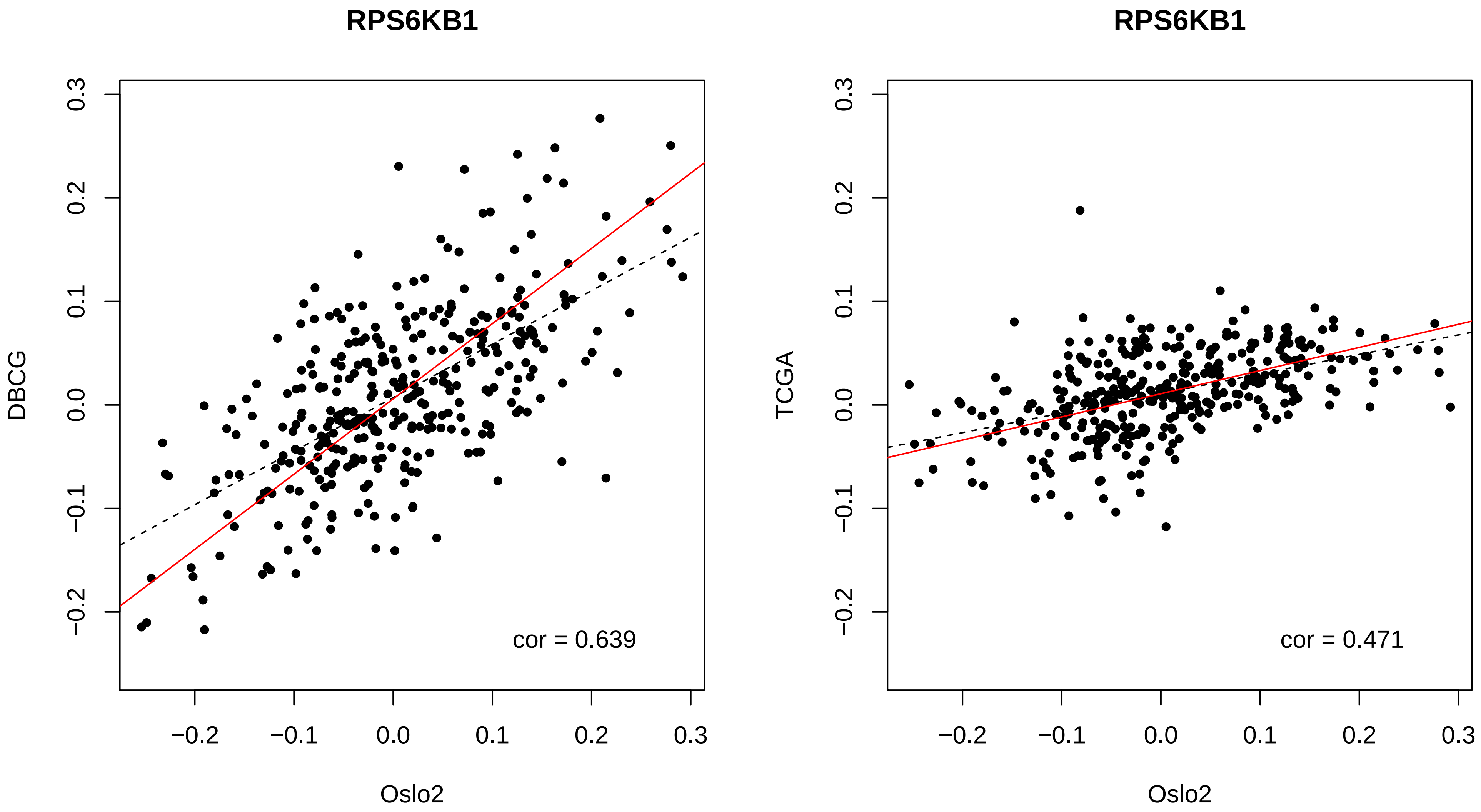

RPS6KB1
DBCG
Oslo2
cor = 0.639
RPS6KB1
TCGA
Oslo2
cor = 0.471
−0.2
−0.1
0.0
0.1
0.2
0.3

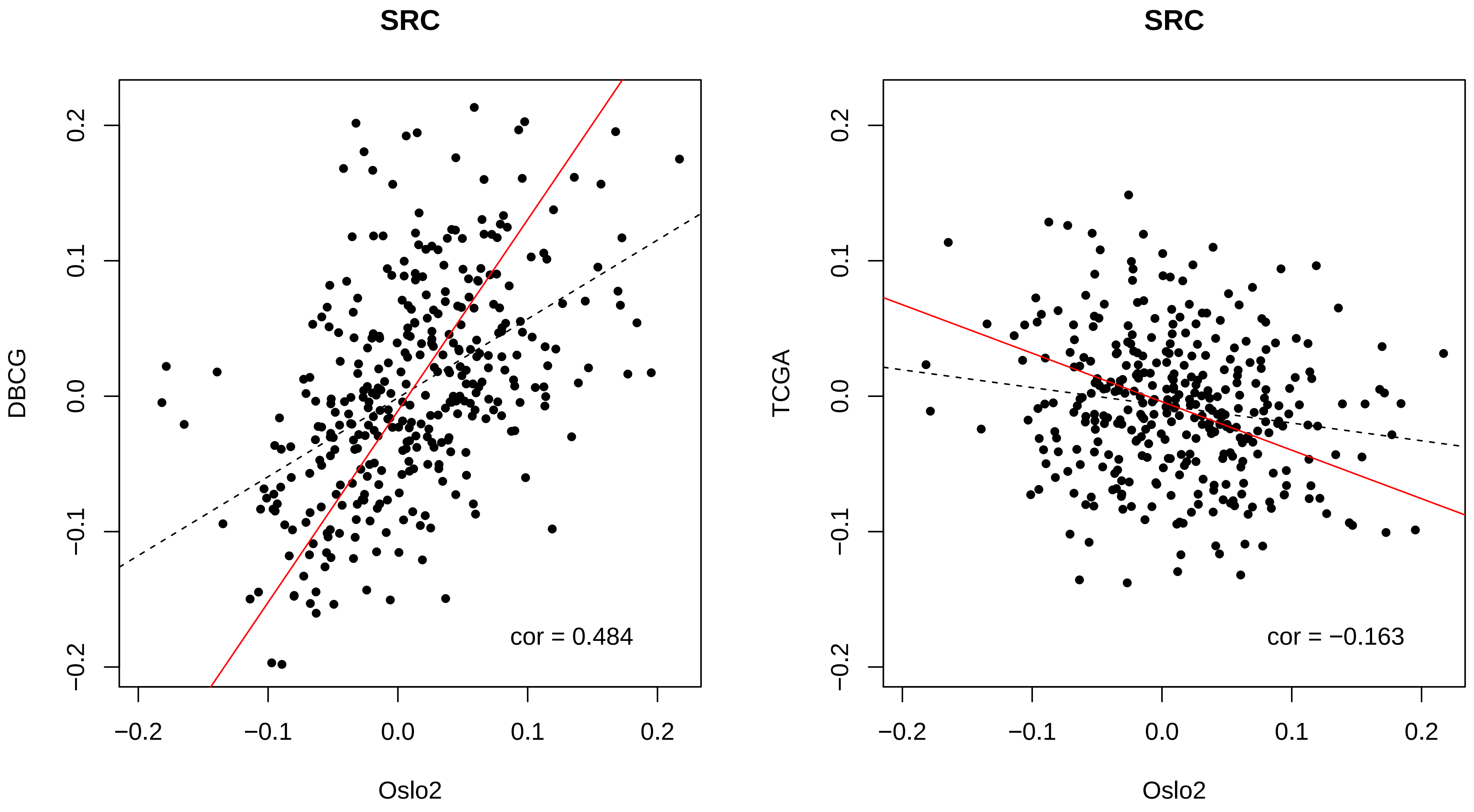
SRC
DBCG
0.2
0.1
0.0
−0.1
−0.2
−0.2
−0.1
0.0
0.1
0.2
Oslo2
cor = 0.484
SRC
TCGA
0.2
0.1
0.0
−0.1
−0.2
−0.2
−0.1
0.0
0.1
0.2
Oslo2
cor = −0.163

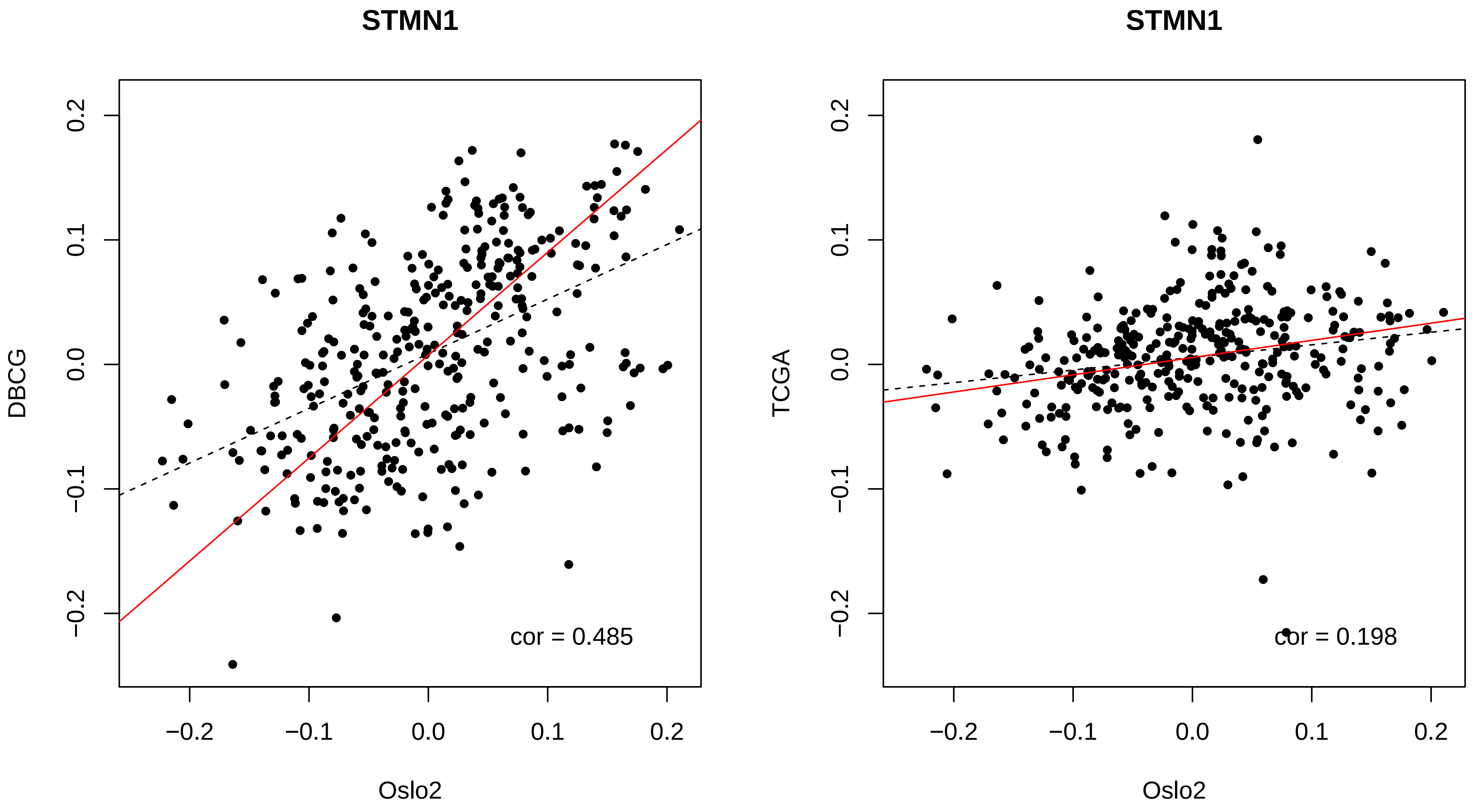
STMN1
DBCG
Oslo2
cor = 0.485
0.2
0.1
0.0
−0.1
−0.2
STMN1
TCGA
Oslo2
cor = 0.198

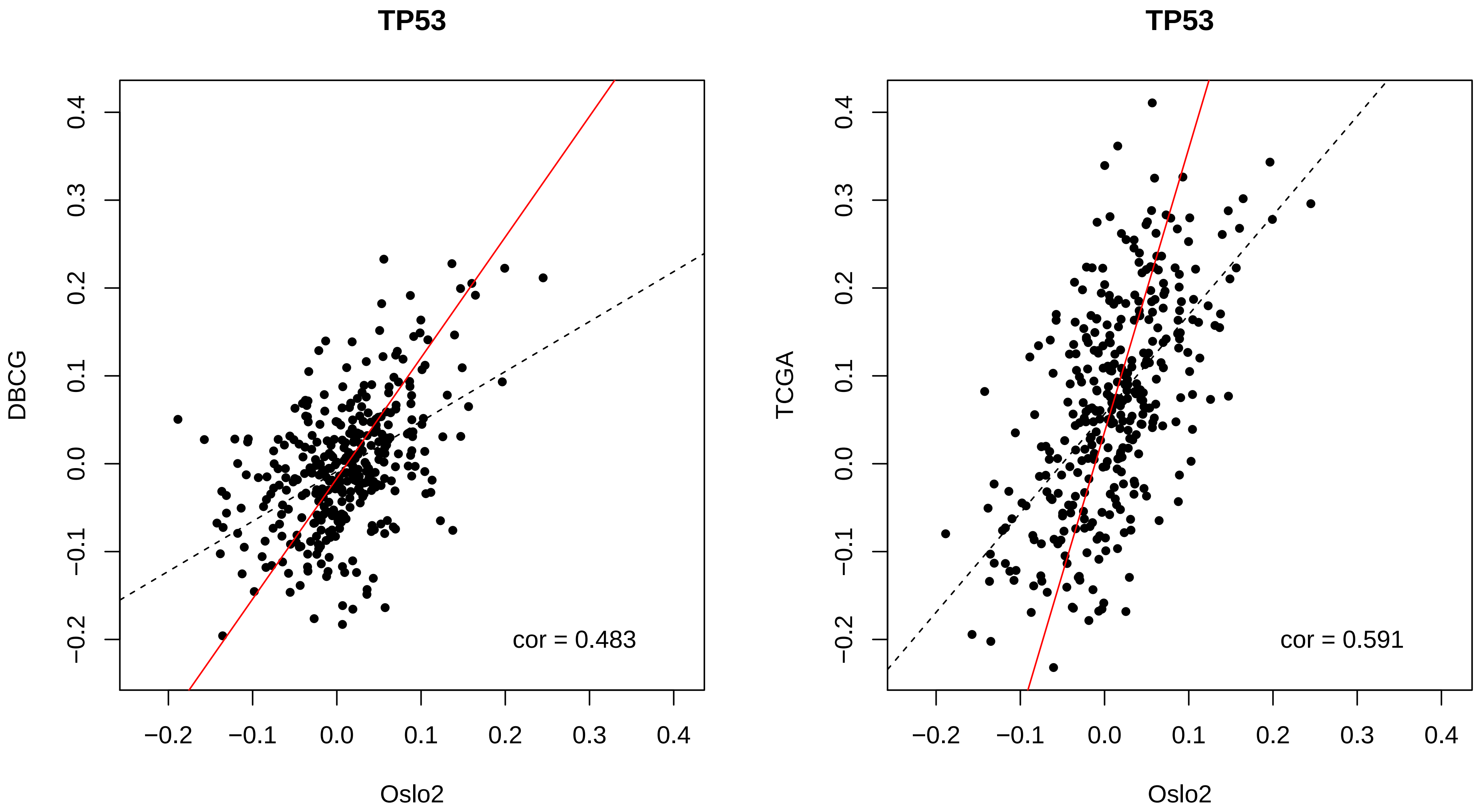
TP53
DBCG
Oslo2
cor = 0.483
TP53
TCGA
Oslo2
cor = 0.591
−0.2
−0.1
0.0
0.1
0.2
0.3
0.4

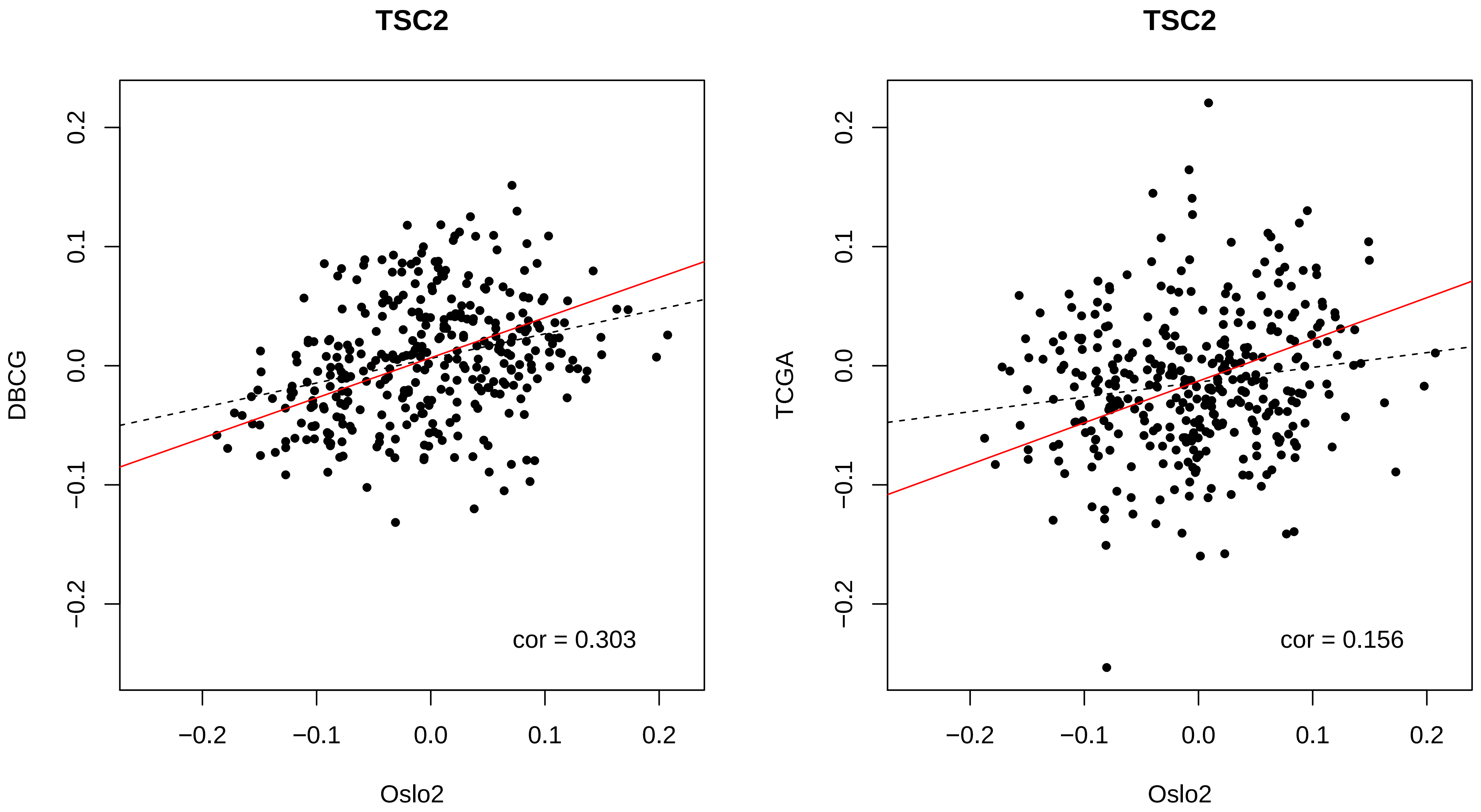
TSC2
DBCG
Oslo2
cor = 0.303
TSC2
TCGA
Oslo2
cor = 0.156
−0.2
−0.1
0.0
0.1
0.2
