# Supplementary material for: Integrated analysis reveals microRNA networks coordinately expressed with key proteins in breast cancer
Source: Genome Med. 2015 Feb 2;7(1):21. doi: 10.1186/s13073-015-0135-5 (PMC4396592; doi:10.1186/s13073-015-0135-5)
Supplement: Additional file 15: — Measured versus predicted protein in the DBCG and TCGA data sets. The multivariate model was fitted to the Oslo2 data set and used to predict protein expression for individual patients in the DBCG and TCGA data sets, using mRNA and miRNA expression values from the respective patients as predictors. The table shows an overview of the correlation between the predicted and the measured protein expression including correlation P-values for the 34 proteins overlapping all three cohorts. The following scatterplots shows the predicted versus the measured protein expression in the DBCG and the TCGA data sets. Solid lines represent a smoothing spline fitted to the data. The Pearson correlation between the predicted versus the measured protein expression is indicated above each plot. [file 13073_2015_135_MOESM15_ESM.pdf]

Gene symbol	Protein	Pearson correlation predicted vs. measured protein in DBCG	Correlation p-value in DBCG	Pearson correlation predicted vs. measured protein in TCGA	Correlation p-value in TCGA
AKT1	Akt1	0.386	0	0.443	0
AKT2	Akt2	0.248	0.005	0.217	0
AKT3	Akt3	0.191	0.031	0.229	0
BCL2	Bcl-2	0.024	0.787	0.811	0
CAV1	Caveolin-1	0.586	0	0.509	0
CCNB1	Cyclin B1	0.792	0	0.752	0
CCND1	Cyclin D1	0.536	0	0.626	0
CCNE1	Cyclin E1	0.605	0	0.822	0
CDH1	E-Cadherin	0.041	0.645	0.572	0
CDKN1B	p27	0.14	0.114	0.685	0
COL6A1	Collagen VI	0.344	0	0.202	0
CTNNB1	beta-Catenin	0.116	0.191	0.22	0
EGFR	EGFR	0.527	0	0.52	0
EIF4EBP1	4EBP1	0.529	0	0.702	0
ERBB2	HER2	0.25	0.004	0.899	0
ESR1	ER-alpha	0.863	0	0.877	0
GSK3A	GSK3-alpha	0.37	0	0.006	0.905
GSK3B	GSK3-beta	0.371	0	-0.038	0.453
KDR	VEGFR2	0.006	0.943	0.342	0
KIT	c-Kit	0.656	0	0.735	0
MAP2K1	MEK1	0.021	0.812	0.078	0.121
MAPK14	p38 MAPK	0.106	0.232	0.347	0
MYC	c-Myc	0.394	0	0.298	0
PECAM1	CD31	0.227	0.01	-0.07	0.162
PGR	PR	0.767	0	0.827	0
PIK3CA	PI3K-p110-alph	-0.006	0.942	0.314	0
PRKCA	PKC-alpha	0.411	0	0.37	0
PRKAA1	AMPK alpha	0.125	0.161	0.415	0
PTEN	PTEN	0.22	0.012	0.248	0
RPS6KB1	p70S6K	0.641	0	0.723	0
SRC	Src	0.039	0.66	0.468	0
STMN1	Stathmin	-0.064	0.475	0.05	0.325
TP53	p53	0.255	0.004	0.43	0
TSC2	Tuberin	0.117	0.188	0.243	0

DBCG predictions

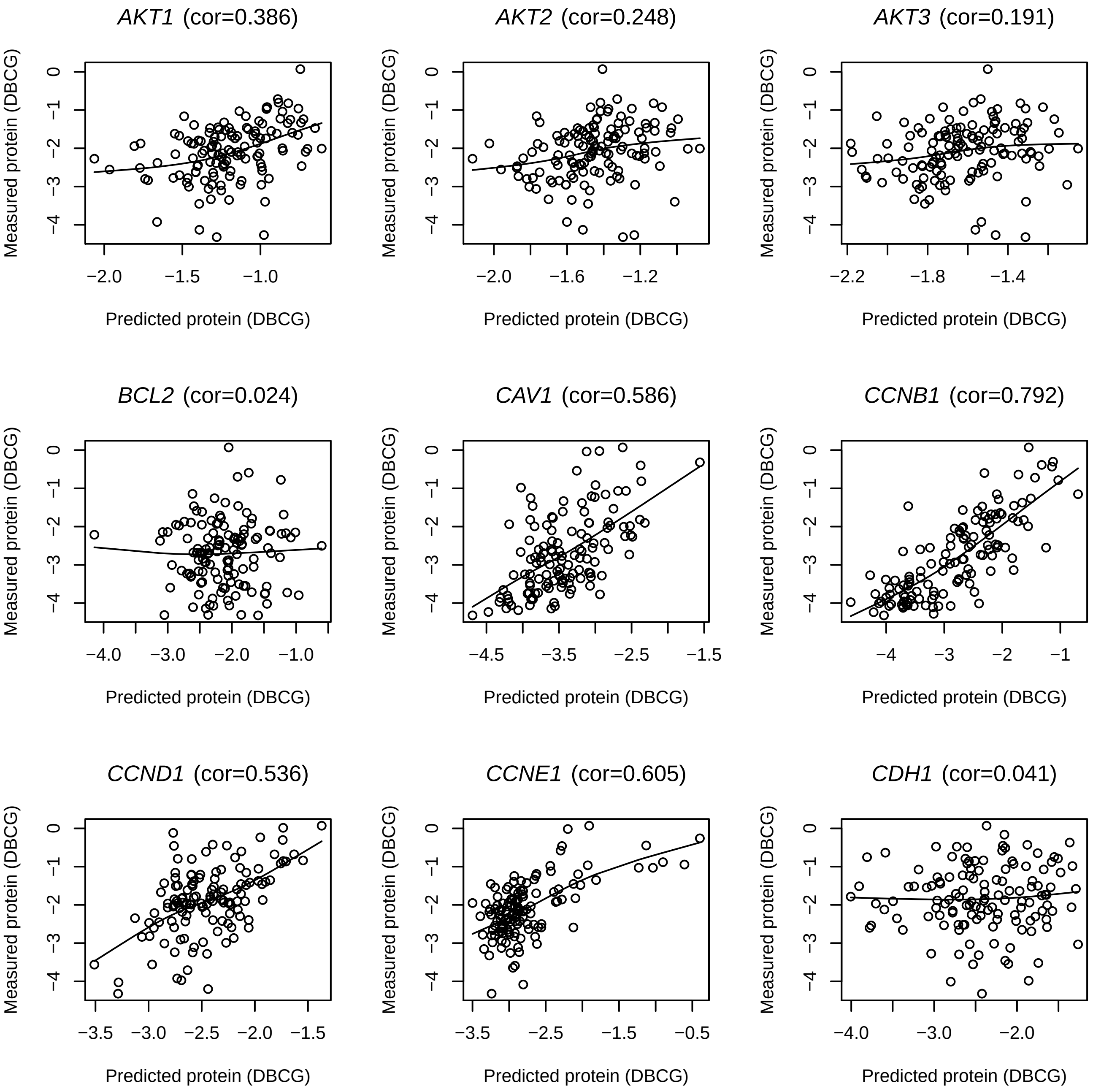
AKT1 (cor=0.386)
AKT2 (cor=0.248)
AKT3 (cor=0.191)
BCL2 (cor=0.024)
CAV1 (cor=0.586)
CCNB1 (cor=0.792)
CCND1 (cor=0.536)
CCNE1 (cor=0.605)
CDH1 (cor=0.041)
Measured protein (DBCG)
Predicted protein (DBCG)

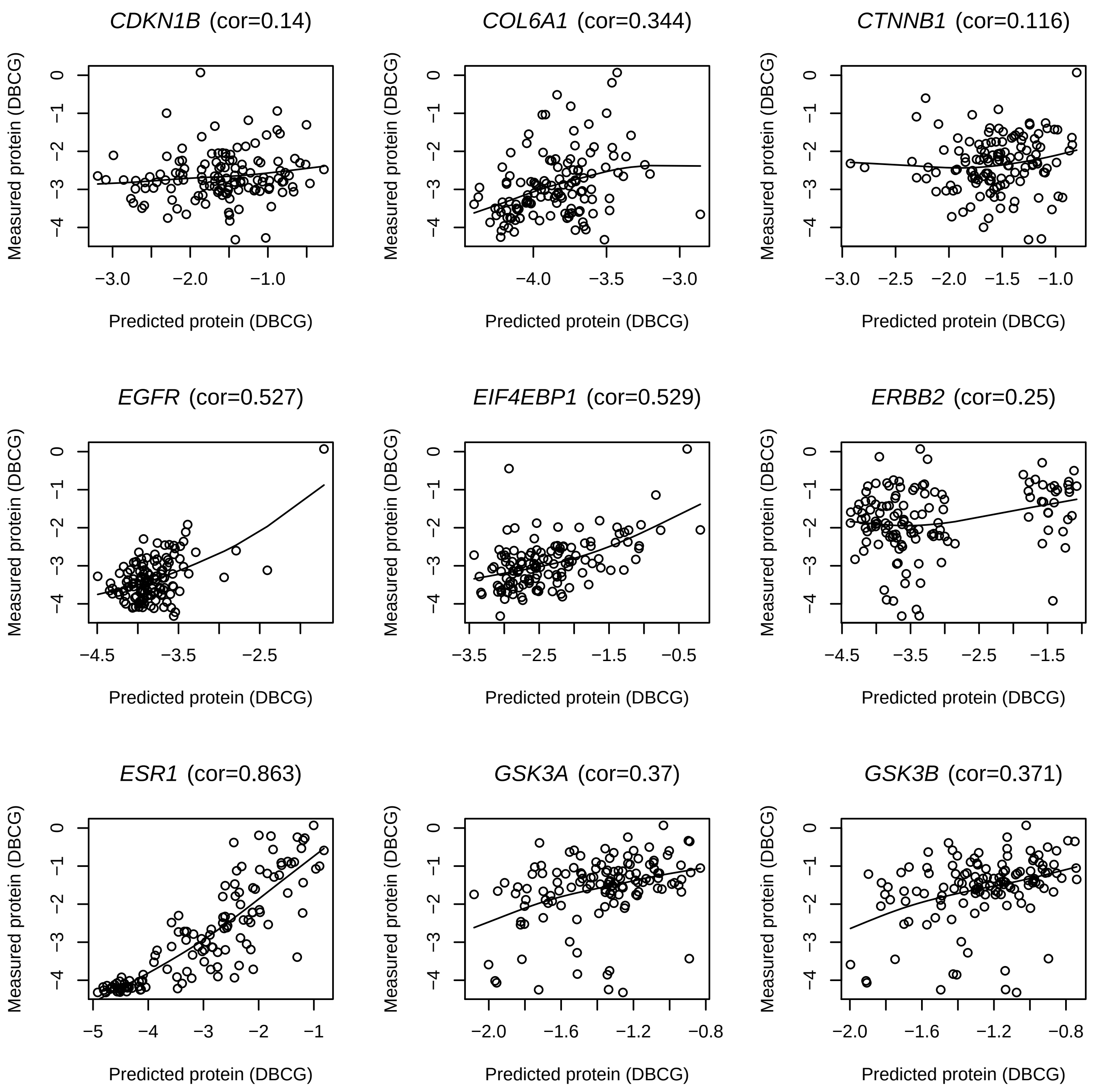

CDKN1B (cor=0.14)
COL6A1 (cor=0.344)
CTNNB1 (cor=0.116)
EGFR (cor=0.527)
EIF4EBP1 (cor=0.529)
ERBB2 (cor=0.25)
ESR1 (cor=0.863)
GSK3A (cor=0.37)
GSK3B (cor=0.371)
Measured protein (DBCG)
Predicted protein (DBCG)

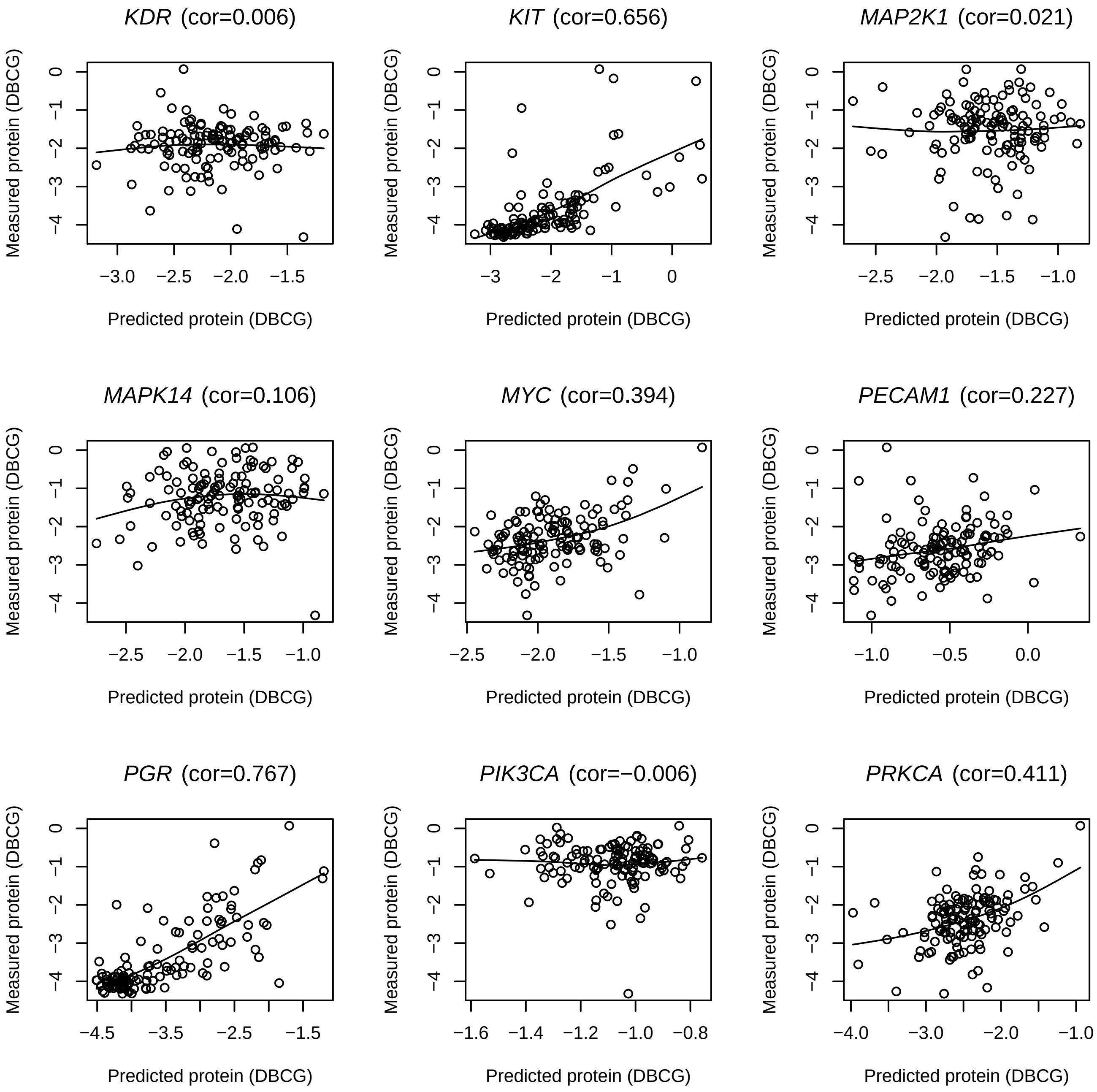
KDR (cor=0.006)
KIT (cor=0.656)
MAP2K1 (cor=0.021)
MAPK14 (cor=0.106)
MYC (cor=0.394)
PECAM1 (cor=0.227)
PGR (cor=0.767)
PIK3CA (cor=−0.006)
PRKCA (cor=0.411)
Measured protein (DBCG)
Predicted protein (DBCG)

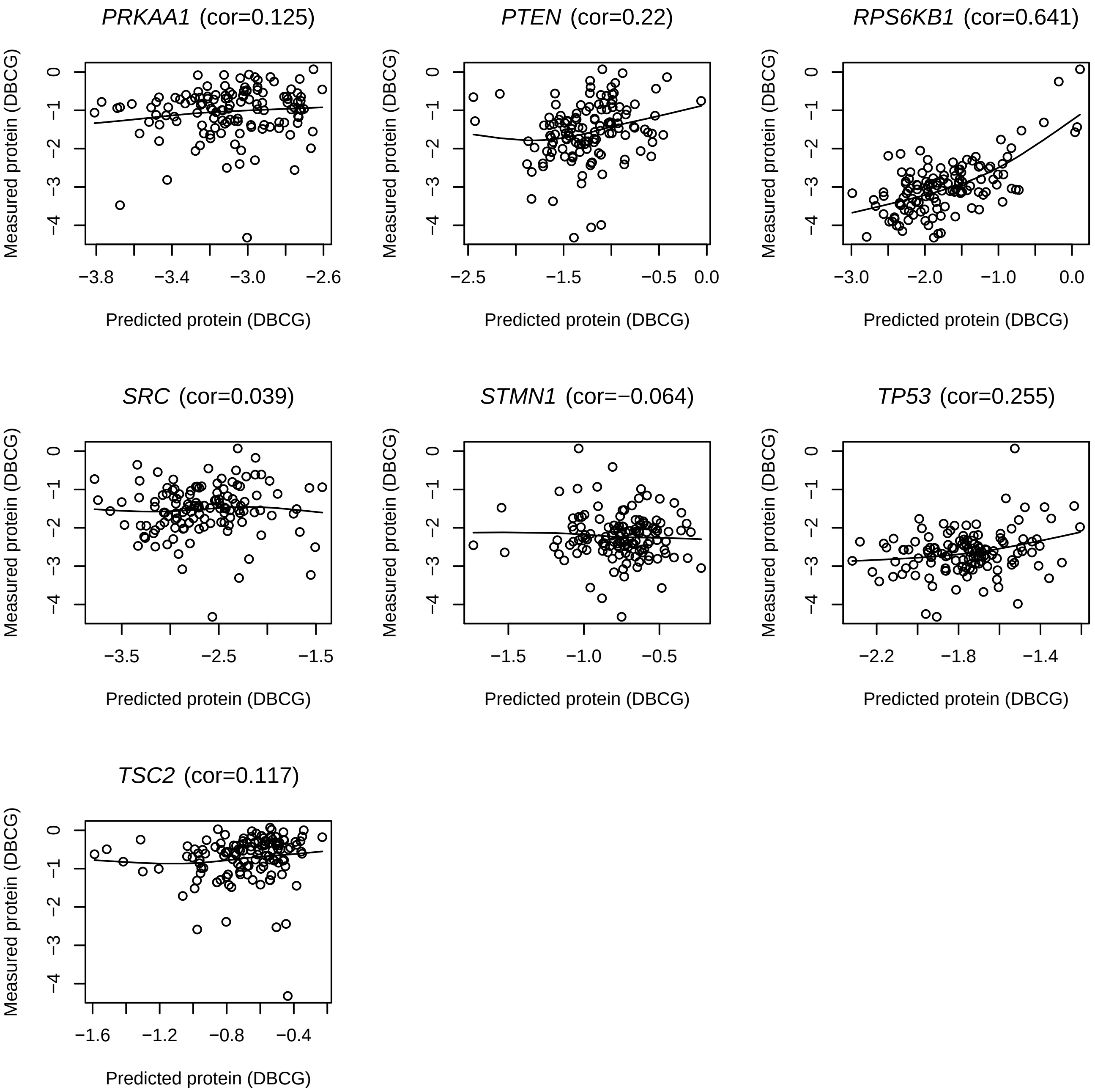
PRKAA1 (cor=0.125)
PTEN (cor=0.22)
RPS6KB1 (cor=0.641)
SRC (cor=0.039)
STMN1 (cor=−0.064)
TP53 (cor=0.255)
TSC2 (cor=0.117)
Measured protein (DBCG)
Predicted protein (DBCG)
0
−1
−2
−3
−4
−3.8
−3.4
−3.0
−2.6
−2.5
−1.5
−0.5
0.0
−3.0
−2.0
−1.0
−3.5
−1.0
−2.2
−1.8
−1.4
−1.6
−1.2
−0.8
−0.4

TCGA predictions

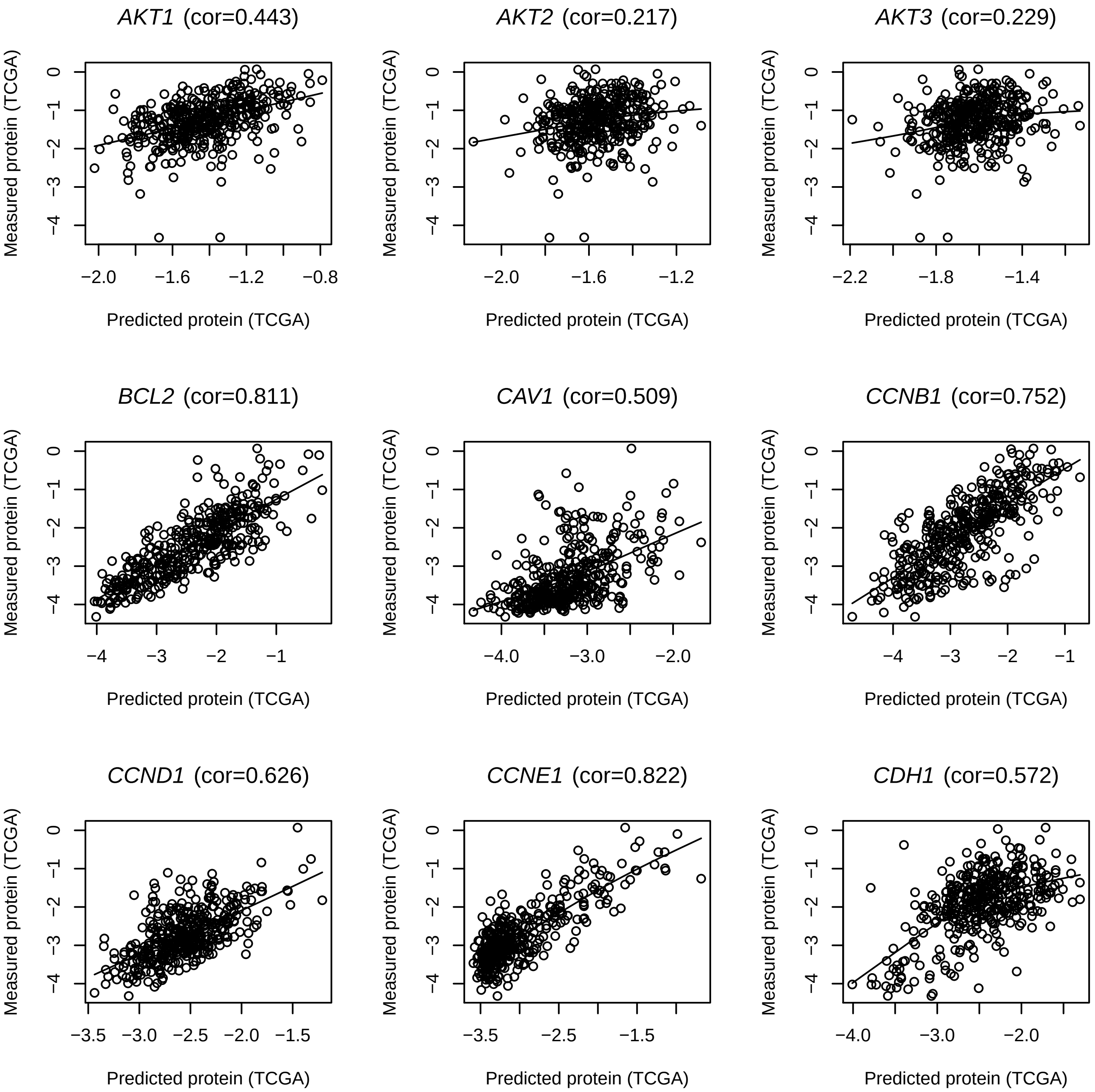
AKT1 (cor=0.443)
AKT2 (cor=0.217)
AKT3 (cor=0.229)
BCL2 (cor=0.811)
CAV1 (cor=0.509)
CCNB1 (cor=0.752)
CCND1 (cor=0.626)
CCNE1 (cor=0.822)
CDH1 (cor=0.572)
Measured protein (TCGA)
Predicted protein (TCGA)

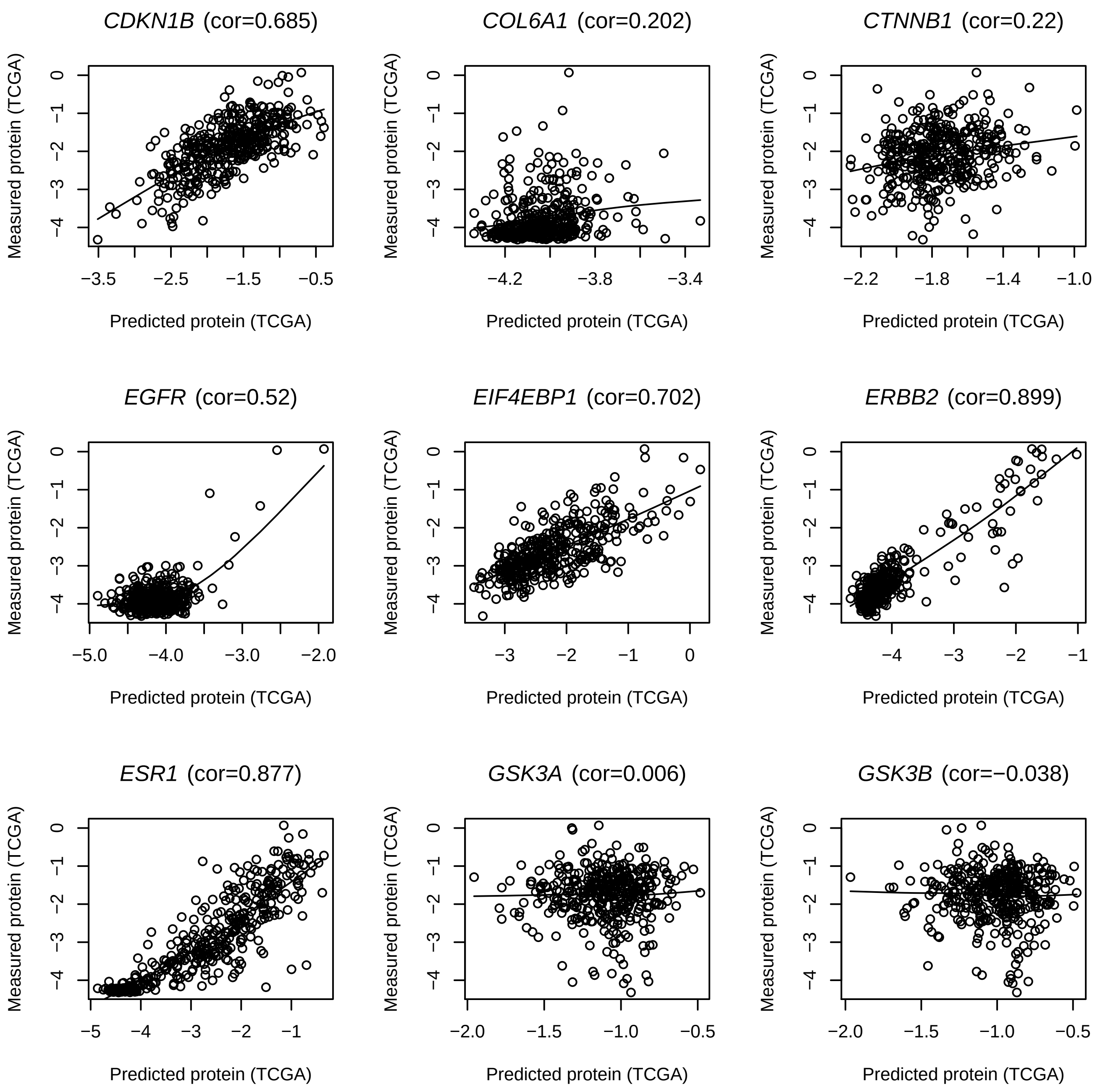
CDKN1B (cor=0.685)
COL6A1 (cor=0.202)
CTNNB1 (cor=0.22)
EGFR (cor=0.52)
EIF4EBP1 (cor=0.702)
ERBB2 (cor=0.899)
ESR1 (cor=0.877)
GSK3A (cor=0.006)
GSK3B (cor=−0.038)
Measured protein (TCGA)
Predicted protein (TCGA)

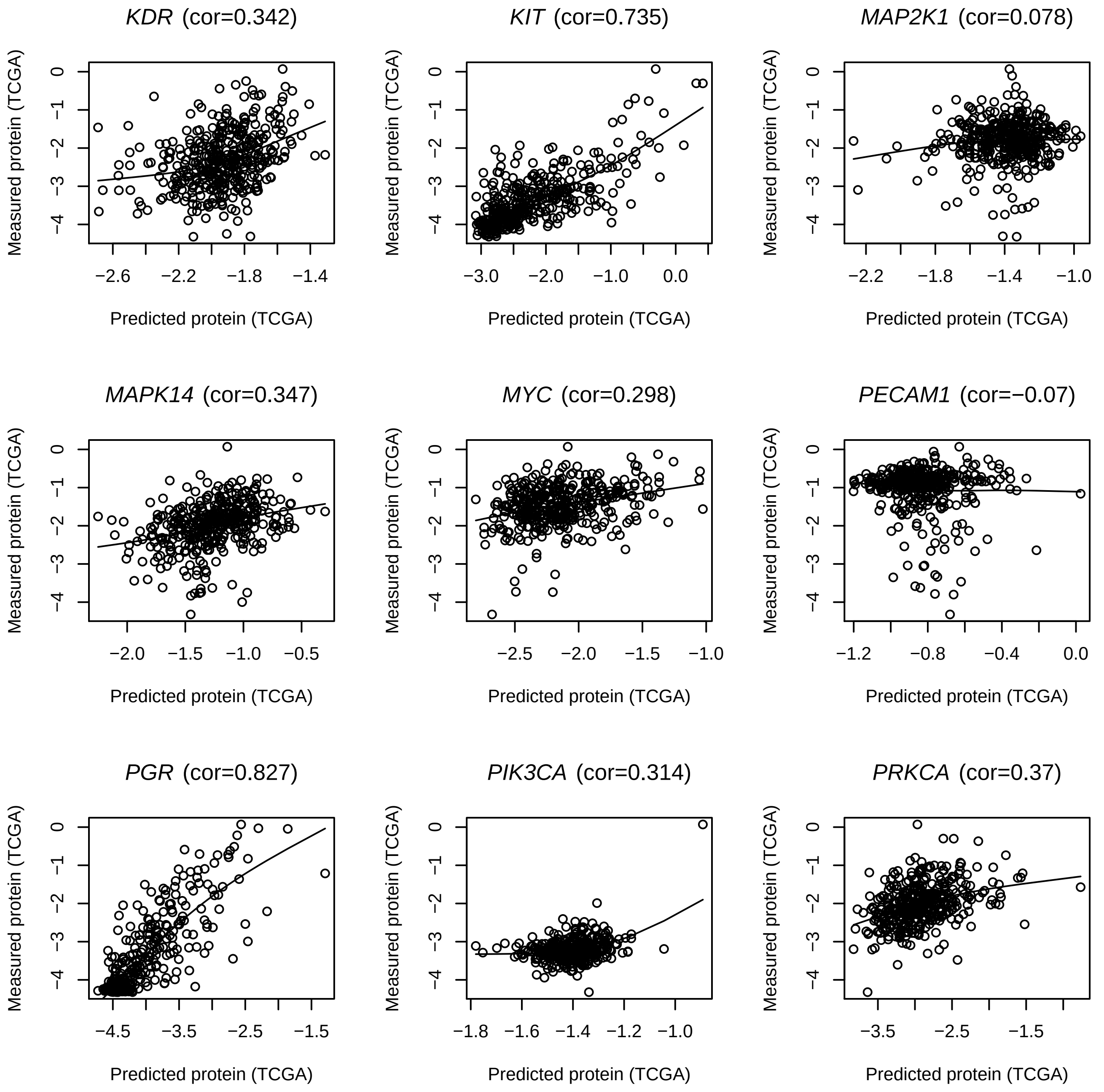
KDR (cor=0.342)
KIT (cor=0.735)
MAP2K1 (cor=0.078)
MAPK14 (cor=0.347)
MYC (cor=0.298)
PECAM1 (cor=−0.07)
PGR (cor=0.827)
PIK3CA (cor=0.314)
PRKCA (cor=0.37)
Measured protein (TCGA)
Predicted protein (TCGA)

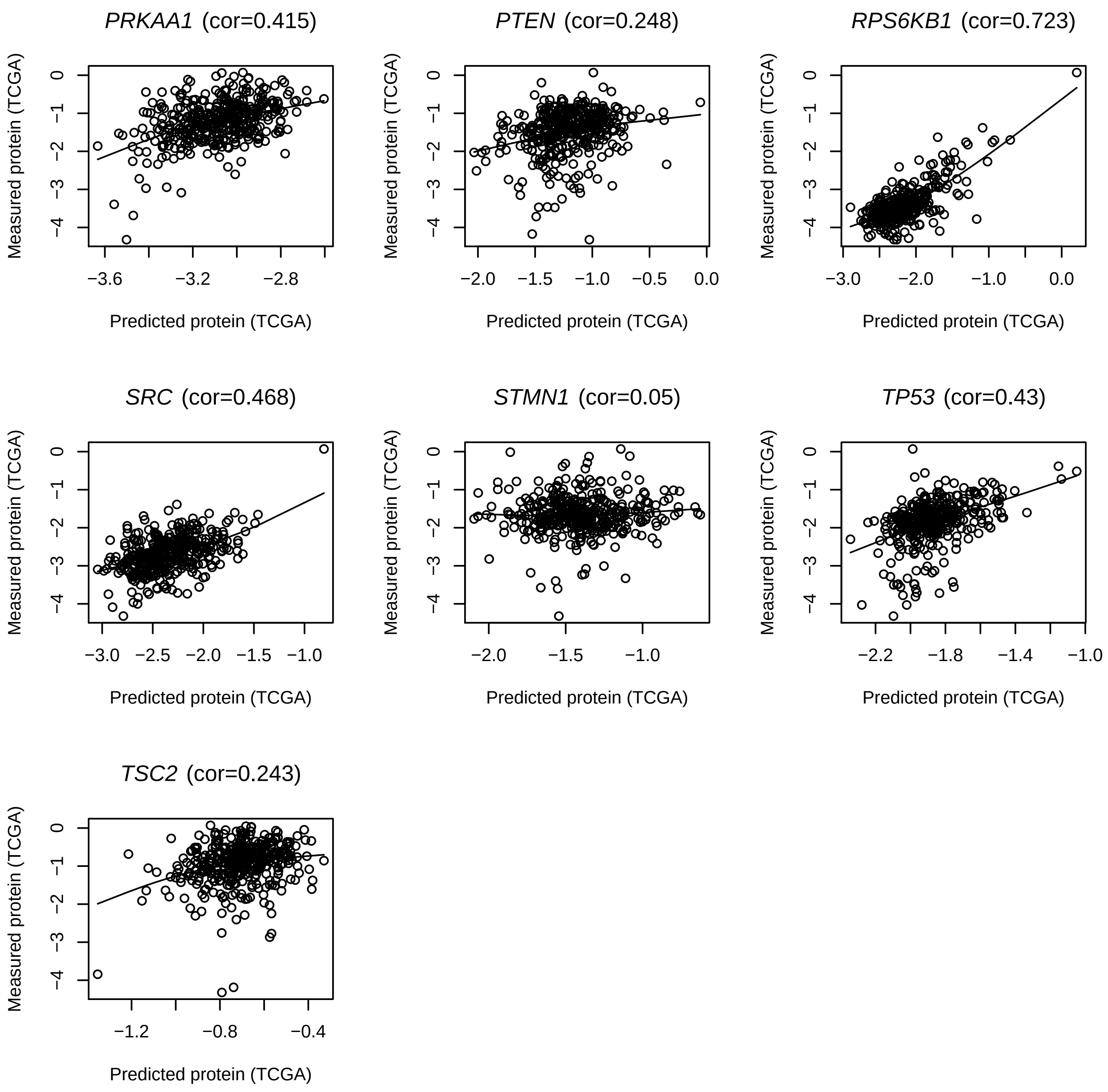

PRKAA1 (cor=0.415)
PTEN (cor=0.248)
RPS6KB1 (cor=0.723)
SRC (cor=0.468)
STMN1 (cor=0.05)
TP53 (cor=0.43)
TSC2 (cor=0.243)
Measured protein (TCGA)
Predicted protein (TCGA)
